# Identification of microRNAs from Amur grape (*vitis amurensis Rupr*.) by deep sequencing and analysis of microRNA variations with bioinformatics

**DOI:** 10.1186/1471-2164-13-122

**Published:** 2012-03-29

**Authors:** Chen Wang, Jian Han, Chonghuai Liu, Korir Nicholas Kibet, Emrul Kayesh, Lingfei Shangguan, Xiaoying Li, Jinggui Fang

**Affiliations:** 1 College of Horticulture, Nanjing Agricultural University, 1 Weigang, Nanjing 210095, China; 2 Zhengzhou Institute of Pomology, Chinese Academy of Agricultural Sciences, 11 Hanghai East Road, Zhengzhou 450009, People's Republic of China

**Keywords:** Amur grape, microRNA, Sequences evolution, Solexa sequencing, miR-RACE, qRT-PCR

## Abstract

**Background:**

MicroRNA (miRNA) is a class of functional non-coding small RNA with 19-25 nucleotides in length while Amur grape (*Vitis amurensis Rupr*.) is an important wild fruit crop with the strongest cold resistance among the *Vitis *species, is used as an excellent breeding parent for grapevine, and has elicited growing interest in wine production. To date, there is a relatively large number of grapevine miRNAs (vv-miRNAs) from cultivated grapevine varieties such as *Vitis vinifera L*. and hybrids of *V. vinifera *and *V. labrusca*, but there is no report on miRNAs from *Vitis amurensis Rupr*, a wild grapevine species.

**Results:**

A small RNA library from Amur grape was constructed and Solexa technology used to perform deep sequencing of the library followed by subsequent bioinformatics analysis to identify new miRNAs. In total, 126 conserved miRNAs belonging to 27 miRNA families were identified, and 34 known but non-conserved miRNAs were also found. Significantly, 72 new potential Amur grape-specific miRNAs were discovered. The sequences of these new potential va-miRNAs were further validated through miR-RACE, and accumulation of 18 new va-miRNAs in seven tissues of grapevines confirmed by real time RT-PCR (qRT-PCR) analysis. The expression levels of va-miRNAs in flowers and berries were found to be basically consistent in identity to those from deep sequenced sRNAs libraries of combined corresponding tissues. We also describe the conservation and variation of va-miRNAs using miR-SNPs and miR-LDs during plant evolution based on comparison of orthologous sequences, and further reveal that the number and sites of miR-SNP in diverse miRNA families exhibit distinct divergence. Finally, 346 target genes for the new miRNAs were predicted and they include a number of Amur grape stress tolerance genes and many genes regulating anthocyanin synthesis and sugar metabolism.

**Conclusions:**

Deep sequencing of short RNAs from Amur grape flowers and berries identified 72 new potential miRNAs and 34 known but non-conserved miRNAs, indicating that specific miRNAs exist in Amur grape. These results show that a number of regulatory miRNAs exist in Amur grape and play an important role in Amur grape growth, development, and response to abiotic or biotic stress.

## Background

Endogenous small RNAs (sRNA) are important regulatory molecules with 19-25 nucleotides (nt) in length [1]. sRNAs can significantly regulate target gene expression levels at transcription or post-transcription levels by guiding mRNA degradation or translational repression [2-5]. MicroRNAs (miRNAs) are one of the main types of non-coding sRNAs and are widespread in organisms, playing important roles in plant growth, development and response to environmental stimuli. Usually, their expression exhibits time-dependent and tissue-specific characteristics across species. An increasing number of studies shows that many miRNAs might be implicated in organ development, cell differentiation and proliferation, cell death and cell apoptosis [6-9].

Currently, hundreds of miRNAs have been identified both by computational and/or experimental approaches in many plants [10-19], with a large number of the identified miRNAs being reported as conserved across various plant species. In addition, quite a number of species-specific miRNAs (*young *miRNAs), that can only be found in one or several plant species and have more evolution in their sequences than conserved (*ancient*) miRNAs have been identified. Usually, it is not easy to identify *young *miRNAs by traditional Sanger sequencing since they accumulate at lower levels in plants and comprise a much smaller fraction in sRNA libraries compared to the other abundant sRNAs. Recent advances in high-throughput sequencing technologies have however contributed significantly to the identification of lowly abundant non-conserved miRNAs in plants, with deep sequencing being successfully employed in the identification of conserved and non-conserved miRNAs in various plant species, including two *Vitis *populations [20-24]. Despite these advances, there is no information available regarding miRNAs in wild grapevine.

China is among the important centers of origin for plants in the *Vitis *genus and has abundant resources of wild grapevine, among which Amur grape has grown to be one of the most important wild fruit crops where it has been cultivated for wine, jam and as a table grapevine. In addition Amur grape is used as a valuable breeding parent for cold tolerance and resistance to devastating grape diseases like downy mildew, powdery mildew and anthracnose. Initiation of molecular biology and genomic studies on Amur grape is therefore of great significance with the identification of miRNAs in Amur grape being an important aspect of research that, to the best of our knowledge, has not been reported. In this study, we first constructed an Amur grape small RNA library and employed high throughput sequencing technology (Solexa) to deep sequence the small RNA library for the identification of new Amur grape miRNAs (va-miRNAs) involved in regulation of cold and/or disease-resistance genes, and berry quality genes. This study also aimed at elucidating evolutionary conservation of these miRNA in other plants and its findings could enrich genomics research on miRNA-based regulatory systems in wild grapevines.

## Results

### Analysis of sequences from small RNA library

To identify new as well as conserved miRNAs in Amur grape, a wild species in the *Vitis *genus, a cDNA library of small RNAs ranging in size from 15 to 30 nt and constructed using pooled RNA isolated from inflorescences, flowers and berries at various stages of development was deeply sequenced by Solexa. A total of 19,692,474 reads were obtained from the sequencing datasets. After removing adaptor sequences, and filtering out low quality reads, 18,902,700 (95.99%) clean reads that included 6,740,185 unique sequences were obtained. Among the clean reads, 8,706,269 (46.06%) matched the grapevine genome, among which 1,030,053 were observed to be similar to known miRNAs, while the other sequences belonged to other types of RNAs, including non-coding RNAs, tRNAs, rRNAs, snRNAs or snoRNAs. The numbers and proportions of these diverse categories of short RNAs are as shown in Table 1. From our library datasets, it was also observed that these small RNAs had uneven distribution in length (Additional file 1), where the class containing sRNAs of 24 nt was the most abundant (36.94%) followed by the 21, 22 and 23 nt sequence classes in that order. The small RNAs with 20-24 nt in size accounted for about 85.00% of the total sequences, which is consistent with the typical sizes of miRNAs from Dicer digestion products [25].

**Table 1:** Distribution of small RNAs in Amur grape

Category	Unique	Percent (%)	Redundant	Percent (%)
exon_antisense	121,519	1.80	301,729	1.60
exon_sense	211,613	3.14	616,910	3.26
intron_antisense	239,906	3.56	447,068	2.37
intron_sense	410,965	6.10	880,478	4.66
miRNA	1,426	0.02	1,030,053	5.45
rRNA	97,064	1.44	1,858,381	9.83
repeat	524,801	7.79	1,094,074	5.79
siRNA	283,866	4.21	2,927,927	15.49
snRNA	5,704	0.08	25,039	0.13
snoRNA	3,790	0.06	12,647	0.07
tRNA	23,935	0.36	842,565	4.46
unann	4,815,596	71.45	8,865,829	46.90

**Total**	**6740185**	**100.00**	**18902700**	**100.00**

### Identification of conserved miRNAs in Amur grape

**Figure 1:**
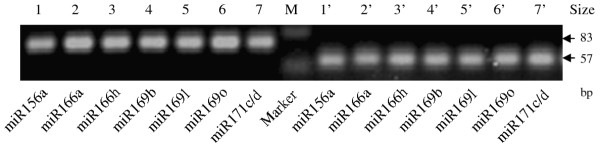
**Comparison of abundance levels of conserved and non-conserved va-miRNAs identified by deep sequencing**.

To identify the known miRNAs in Amur grape, we first compared the sequences from our sRNA library to known miRNAs from other plant species deposited in miRBase 16.0 http://www.mirbase.org/. Among the 18,902,700 sequences screened, 1,030,053 unique sequences representing 126 conserved miRNAs were found to be orthologs of known miRNAs that had been earlier reported, and were conserved in Amur grape (Additional file 2). These identified va-miRNAs belong to 27 miRNA families and have been shown to be conserved in a variety of plant species according to a comparative genomics-based analysis [26]. Since deep sequencing may provide an alternative way to estimate the expression profiles of protein coding genes and/or miRNA genes [27,28], the millions of Amur grape sRNA sequences yielded by Solexa sequencing in this study can also allow us to estimate the abundance of various miRNA families and even that of different members in a given family [18]. This study shows that va-miRNA families display dramatic variations in copy number ranging from 2 to 372,442 (Additional file 2), indicating significant discrepancy of their accumulation in Amur grape. Eighty percent of these va-miRNAs have over 50 copies, with those having > 1,000 copies accounting for about 50%, while only four va-miRNAs (va-miR159a, va-miR159b, va-miR169i, va-miR171h) were sequenced less than 10 times (Figure 1) which is consistent with previous reports [18,27,29] that the most conserved miRNAs are highly expressed in organisms.

To further identify the sequence divergences of orthologous miRNAs from diverse species of vitis plants, we compared conserved orthologous miRNAs between Amur grape and wine grapevine (released in miRbase 16.0) [20,21]. Results show that 38 unique conserved miRNA sequences of exhibited 1-3 nucleotide divergence at both ends of the sequence (Table 2). This phenomenon was also found in the alignment analysis between orthologous miRNAs from Amur grape and table grapevine [23], in which 12 unique orthologous sequences were observed to have 1-3 termini base divergences (Table 3). These sequence variations may reflect some evolution events that happened to the miRNAs in grapevine along with the speciation of the species, consistent with the reports in *Arabidopsis thaliana *and *Arabidopsis lyrata *[13,30]. Reverse transcription, amplification or sequencing could also be a cause of some of the nucleotide divergence observed [31].

**Table 2:** List of miRNAs with nucleotide variations between *Vitis amurensis *Rupr. and *Vitis vinifera*

MiRNA	***Vitis amurensis *Rupr**.	MiRbase 16.0 (*Vitis vinifera*)
MiR156a/i	TTGACAGAAGAGAGGGAGCAC	TGACAGAAGAGAGGGAGCAC
MiR159a/b	CCTTGGAGTGAAGGGAGCT	CTTGGAGTGAAGGGAGCTCTC
MiR166a	TCTCGGACCAGGCTTCATTCC	TCGGACCAGGCTTCATTCCTG
MiR166c/e/h	TCGGACCAGGCTTCATTCCCC	TCGGACCAGGCTTCATTCCCCC
MiR166d/f/g	TCGGACCAGGCTTCATTCCCC	TCGGACCAGGCTTCATTCCCCT
MiR167a	TGAAGCTGCCAGCATGATCT	TGAAGCTGCCAGCATGATCTG
MiR167b/e	TGAAGCTGCCAGCATGATCTAA	TGAAGCTGCCAGCATGATCTA
MiR169b/h	TGAGCCAAGGATGGCTTGCCGT	TGAGCCAAGGATGGCTTGCCG
MiR169e	TAGCCAAGGATGACTTGCCT	TAGCCAAGGATGACTTGCCTG
MiR169i	TGAGCCAAGGATGACTGGCCGT	GAGCCAAGGATGACTGGCCGT
MiR169l	TGAGCCAAGGATGACTTGCCG	GAGCCAAGGATGACTTGCCGT
MiR169m/p	TGAGCCAAGGATGACTTGCCG	GAGCCAAGGATGACTTGCCGG
MiR169n/q	TAGAGCCAAGGATGACTTGCCG	GAGCCAAGGATGACTTGCCGG
MiR169o	TGAGCCAAGGATGACTTGCCG	GAGCCAAGGATGACTTGCCGC
MiR169r	TGAGTCAAGGATGACTTGCCGA	TGAGTCAAGGATGACTTGCCG
MiR169t	CGAGTCAAGGATGACTTGCCGA	CGAGTCAAGGATGACTTGCCG
MiR169x	TAGCCAAGGATGACTTGCCT	TAGCCAAGGATGACTTGCCTA
MiR171a	TGATTGAGCCGTGCCAATATC	TTGAGCCGTGCCAATATCATG
MiR171b	TTGAGCCGCGTCAATATCTCC	TGATTGAGCCGCGTCAATATC
MiR171c/d	TGATTGAGCCGTGCCAATATC	TTGAGCCGTGCCAATATCACG
MiR171e	TTGAGCCGCGCCAATATCACT	TGATTGAGCCGCGCCAATATC
MiR171h	TTGAGCCGCGCCAATATCCCG	TGGTTGAGCCGCGCCAATATC
MiR172d	TGAGAATCTTGATGATGCTGC	AGAATCTTGATGATGCTGCAT
MiR319b	TTGGACTGAAGGGAGCTCCCT	CTTGGACTGAAGGGAGCTCCC
MiR319c/f	TGCTTGGACTGAAGGGAG	CTTGGACTGAAGGGAGCTCCC
MiR319g	TTGGACTGAAGGGAGCTCCC	ATTGGACTGAAGGGAGCTCCC
MiR393a/b	TCCAAAGGGATCGCATTGAT	TCCAAAGGGATCGCATTGATCC
MiR396a	TTCCACAGCTTTCTTGAA	TTCCACAGCTTTCTTGAACTA
MiR396b	TTCCACAGCTTTCTTGAA	TTCCACAGCTTTCTTGAACT
MiR396c/d	TTCCACAGCTTTCTTGAA	TTCCACAGCTTTCTTGAACTG
MiR398a	TTCTCAGGTCACCCCTTTGGG	TGTGTTCTCAGGTCACCCCTT
MiR399a	CAAAGGAGAATTGCCCTGTTA	TGCCAAAGGAGAATTGCCCTG
MiR399d	AAAGGAGATTTGCTCGTGAAT	TGCCAAAGGAGATTTGCTCGT
MiR399h	TGCCAAAGGAGAATTGCC	TGCCAAAGGAGAATTGCCCTG
MiR403a/b/c/d/e/e\f	TTAGATTCACGCACAAACT	TTAGATTCACGCACAAACTCG
MiR482	TCTTTCCTACTCCTCCCATTCC	CCTACTCCTCCCATTCC
MiR535a/b/c/d/e	TGACAACGAGAGAGAGCACGC	TGACAACGAGAGAGAGCACGCT

**Table 3:** List of orthologous conserved miRNAs with nucleotide divergence between Amur grape and 'Summer Black' grape (hybrid of *V. vinifera *and *V. labrusca*)

MiRNA	Amur grape	Summer Black grape
MiR159a/b	CCTTGGAGTGAAGGGAGCT	TTGGAGTGAAGGGAGCTCTC
MiR167a	TGAAGCTGCCAGCATGATCT	TGAAGCTGCCAGCATGATCTG
MiR169e	TAGCCAAGGATGACTTGCCT	TAGCCAAGGATGACTTGCCTGC
MiR169u	TGAGTCAAGGATGACTTGCCG	TGAGTCAAGGATGACTTGCCGT
MiR319b	TTGGACTGAAGGGAGCTCCCT	TTGGACTGAAGGGAGCTCCC
MiR319c/f	TGCTTGGACTGAAGGGAG	TTGGACTGAAGGGAGCTCCC
MiR393a	TCCAAAGGGATCGCATTGAT	TTCCAAAGGGATCGCATTGAT
MiR397a/b	TCATTGAGTGCAGCGTTGATG	CATTGAGTGCAGCGTTGATGA
MiR398a	TTCTCAGGTCACCCCTTTGGG	TGTGTTCTCAGGTCACCCCTT
MiR399a	CAAAGGAGAATTGCCCTGTTA	TGCCAAAGGAGAATTGCC
MiR399b/c	TGCCAAAGGAGAGTTGCCCTG	GCCAAAGGAGAGTTGCCCT
MiR399d	AAAGGAGATTTGCTCGTGAAT	TCTGCCAAAGGAGATTTGCTC
MiR399i	CGCCAAAGGAGAGTTGCCCTG	CGCCAAAGGAGAGTTGCCC

### Identification of non-conserved va-miRNAs

Among critical features that can be used to distinguish miRNAs from other small RNAs is the ability of a miRNA's flanking sequences to fold-back in a hairpin structure [32]. Based on the available grapevine genome sequence, we identified flanking sequences of the candidate va-miRNAs and predicted their possibility of forming characteristic hairpin structures (Additional file 3). Our analysis revealed that among the small RNA sequences matching the grapevine genome in these datasets, there were 3,537,694 sequences without any annotation, and whose flanking regions could be subjected to secondary structure analysis for prediction of new miRNA candidates. Our search for these new potential miRNAs revealed that 128,262 sequences, belonging to 106 unique sequences, were able to meet the new criteria of miRNA annotation set by Meyers *et al. *[32] and were thus considered as potential new va-miRNAs (Table 4). These newly identified va-miRNAs were 20-23 nucleotides long, and the negative folding free energies of their precursors ranged from -20.8 kcal/mol to -124.6 kcal/mol except for va-miR066 and va-miR082 with -19.7 kcal/mol, and the average folding free energies of all these precursors were about -46.45 kcal/mol, which is similar to those of other plant miRNA precursors [33]. Although va-miR066 and va-miR082 have slightly higher free energies over -20 kcal/mol, one of the criteria of miRNAs annotation, our miR-RACE PCR could validate their real identity in Amur grape (Additional file 4). In addition, the precursor sequences for these 106 va-miRNAs can be mapped to the grapevine genome, whereby 71 match perfectly to grape ESTs datasets (Table 4). The precursors of these conserved miRNAs were 65-311 nt long, where 66.1% were 65-150nt long, a finding similar to that observed in *Arabidopsis *and rice [34]. More importantly, out of these 106 va-miRNA sequences, 55 start with a 5' uridine, which is also an important characteristic feature of miRNAs. Considering that anti-sense miRNA (miRNA*) sequences are also an important criterion for identification of miRNAs [27,28,33,35], we also searched for miRNAs* in our datasets where a total of 31 miRNAs* for new va-miRNAs candidates were found, providing evidence supporting them as new miRNAs. The remaining predicted new miRNAs without corresponding supporting miRNA* found can be considered as potential new miRNAs, which can be due to that the level of corresponding miRNAs* were to low to be detected easily. As reported that the average frequency of miRNA* were established to be about 10% of that of mature miRNA [27], since quite a number of potential new miRNAs in our library had fewer than 10 reads observed, their corresponding miRNAs* could also be of a much lower frequency hence not easily to be sequenced. All characteristics of the predicted miRNAs mentioned above could indicate that they were potential new miRNAs in Amur grape. To further confirm whether or not these new miRNAs were Amur grape-specific, we compared them with those identified and reported in other wine and table grapevine cultivars [20-24]. Results show that 72 out of the 106 newly identified miRNAs were discovered for the first time and thus might be Amur grape-specific; while the remaining 34 were found to also exist in the other vitis and a few other non vitis plant species (Table 4).

**Table 4:** List of identified non-conserved miRNAs in Amur grape

MiRNA ID	Sequences (5→3')	Reads	Stem-loop length (nt)	Start in Chr	MFE	EST No. in *Vitis *genus	EST No. in other plants	MiRNAs homologs in other *Vitis *species	miRNA*
va-miR001^a^	TTTTTTTATTGGATCCGTCGGGA	108	84	13:9154663	-21.4	AM473732.1; AM473732.1;	GU363535.1		
va-miR002	TTTTGTTTTTATTGTTGTTTT	14	240	16:6817573	-42.05	AM432096.2; AM436712.2			
va-miR003	TTTTCTTTTCCTGCATTTTCT	21	196	10_random:812676	-28.77	AM454974.2			
va-miR004	TTTTCCTATGATTTCTTGGCA	18	139	17:4715183	-44.23	AM451502.1			
va-miR005	TTTGCATGAGGGGGGATTGTCAT	8	160	14:10210052	-55.9	AM425582.2			
va-miR006^a ^-1	TTTCTTAGCAACCAAACAGAG	10	213	3:3982162	-36.6	AM453475.2	AC210553.1		
va-miR006^a ^-2	TTTCTTAGCAACCAAACAGAG	8	213	3:3995013	-36.6	AM453475.2	AC210553.1		
va-miR006^a ^-3	TTTCTTAGCAACCAAACAGAG	8	255	19:4998280	-74.2	AM453475.2	AC210553.1		
va-miR007 ^b^	TTTCCGACTCGCACTCATGCCGT	812	103	17:5737883	-49	No		Vv-miR076^c^	Y
va-miR008 ^a^	TTTCCACGGCTTTCTTGAACT	16,273	182	1:1942578	-57.1	AM481354.2	BT117687.1	Vv-miR075^c^	Y
va-miR009	TTTCCACATCTTTCTTGAACT	10	113	12:7648535	-49.5	AM480580.2			Y
va-miR010 ^a^	TTTAATTTAAATATTGAAGAT	13	220	8:10844562	-43.6	no	AC216916.1; AC212928.1		
va-miR011^b^	TTGTCGCAGGAGAGACGGCACT	6	92	14:17492727	-57.8	AM479856.1		MirC6^d^	Y
va-miR012	TTGGCACATTATCTAACAACT	14	107	8:2707092	-21.1	No			
va-miR013	TTGCTGAGAGAGTCGTCTGCC	53	81	9:13023036	-38.5	No			Y
va-miR014	TTGCTAAATCTTTGATTCGATC	20	103	13:10216545	-37.52	AM443747.2			
va-miR015	TTGCTAAATCATTGATTCGATC	11	101	13:10213352	-34.2	AM439308.2; AM447569.2			Y
va-miR016	TTCTTGTGATCTTGTTGTTTC	9,441	148	5:20754701	-88.9	AM454776.2			
va-miR017	TTCTTCTTAACGTCTGACTTA	10	135	1:1002143	-57.6	AM448689.1			
va-miR018 ^a^	TTCTCGGACCAGGCTTCATTC	148	161	7:14570131	-64.3	no	BT089825.1	Y	
va-miR019	TTCAAGTCAAAGTCGAACAAG	16	71	1:15194533	-20.2	no			
va-miR020	TTATTAACCATTTAAATTTA	5	212	4:14934239	-21.55	AM458370.2			
va-miR021^b^	TTATGTGAGTGTTCGGCAAATC	16	90	5:20305533	-37.9	no		Vv-miR073^c^	
va-miR022-1	TTAGTTTGAACTAGGAGATGACA	10	113	10:6246751	-20.8	no			
va-miR022-2	TTAGTTTGAACTAGGAGATGACA	10	255	10:6328614	-49.9	no			
va-miR023 ^b^	TTAGATGATCATCAACAAACA	9,128	118	5:23154645	-45.5	AM429664.2		Vv-miR072^c^	Y
va-miR024	TTACTTTTATCTGAATAGAAA	8	108	15:3408926	-26.7	AM465770.2			
va-miR025 ^b^	TTACACAGAGAGATGACGGTGG	633	105	5:6660435	-50.04	AM429841.2		MirC22^d^	Y
va-miR026	TGGTAAATTGGTTTAAATATTC	11	192	18:19596668	-22.4	AM487707.2; AM486523.2; AM442872.2; AM465537.1			
va-miR027 ^a^	TGGATGCATGTAGCTTGTCAA	21	103	18:4024355	-71.9	AM484186.2;	AK329204.1; XM_002892267.1	Y	
va-miR028	TGGATACACTTTTTATTTTTT	6	204	10:2463091	-29.5	AM467500.2; AM425302.2; AM476399.1			
va-miR029^b ^-1	TGGAGAAGGGGAGCACGTGCA	5	118	14:1404606	-59	AM462494.2;		Vv-miR068^c^	
va-miR029^b ^-2	TGGAGAAGGGGAGCACGTGCA	6	119	7:3651397	-45.9	AM462494.2;		Vv-miR068^c^	
va-miR030	CGCCGCTCTCCTGTGACAAGA	6	92	14:17492727	-57.8	AM479856.1			
va-miR031^b^	TGCATTTGCACCTGCACCTTA	104	165	6:22063225	-75.22	No		Vv-miR020*^c^	Y
va-miR032	TGATTGTAGAAAATGTTTTTAAC	5	111	6:21035897	-36.7	AM439539.1			
va-miR033	TGATATTAGCAGCTGAGAACA	10	147	14:11536480	-70	AM449587.2			Y
va-miR034 ^b^	TGAGTAGTGGACTATCGCATG	9	116	17:7362816	-43.9	AM489347.2; AM461823.2;	Vv-miR065^c^		
va-miR035	TGAGGAAGGGTGTTAGAGTAC	13	90	13:5595054	-46.1	No			
va-miR036 ^b^	TGACCGGCTCTTATCTCTCATG	27	189	17:351716	-79.3	AM447150.1		Vv-miR063^c^	Y
va-miR037	TGACACTATATAAATATGAA	6	244	2:8067706	-48.1	AM430446.2; AM466868.2;			
va-miR038	TGAATGGTTGGTAAATTGAG	11	150	12:14954776	-40.6	AM482866.2; AM466422.2;			
va-miR039 ^b^	TGAAATGTAGGCAAGGAAAAG	9	192	16:8071148	-53.6	AM478700.2;		Vv-miR059^c^	
va-miR040	TCTGTTTGGACGCCGAGAAAA	7	217	17:10557309	-62.22	No			
va-miR041^b^	TCTGTCGCAGGAGAGATGATGC	30	103	14:17809215	-53.9	No		MirC23^d^	Y
va-miR042	TCTGTACTTTAAGAATCTGGCTT	15	208	15:6195235	-41.2	AM454626.2			Y
va-miR043	TCTGCATTTGCACCTGCACCT	15	165	8:17999572	-57.1	No			
va-miR044	TCTCTTGATATTAGTAGCTGA	9	138	14:11536485	-71.9	No			
va-miR045	TCGAGTGGGAACGCATTGAGC	6	150	12:13192937	-35.7	AM486659.2			
va-miR046^b^	TCCCAGGAGAGATGGCACCTGC	330	85	17:5984990	-43	AM457000.2		Vv-miR055^c^	
va-miR047 ^a^	TCACAAGTTCATCCAAGCACCA	32,758	136	18_random:5237347	-51.87	No	EZ339498.1	Vv-miR054^c^	Y
va-miR048	TCAATTAGTAGCTTAACATGG	8	132	11:5115765	-58.49	AM471165.2			
va-miR049^b^	TCAATAAGGTACTTTTAGCT	108	126	11:12939237	-34.3	AM487033.2		Vv-miR053^c^	Y
va-miR050	TCAACTTGATTTTTGGATGAG	9	101	8:15743876	-27.8	AM460148.2			
va-miR051	TCAAAAGAGAAAATGTGGATG	46	100	6:4462385	-35.9	AM428913.2			
va-miR052	TATTAAGTTCAAGTGAAAATT	5	206	10_random:1196749	-64.5	AM473600.2			
va-miR053	TAGTGATTTTAGGAAGCGTTT	7	271	16:432019	-45.26	AM455590.2			
va-miR054 ^b^	TAGGAAACGTTTCTACTCTTT	20	311	scaffold_1047:19353	-59.7	No		Vv-miR051^c^	
va-miR055 ^b^	TAATCTGCATCCTGAGGTCTA	48	107	8:14329491	-44.9	AM426746.2		Vv-miR048^c^	Y
va-miR056-1	TAAAATCACTATCAAACGAA	15	133	10:1636145	-21	AM454228.2			
va-miR056-2	TAAAATCACTATCAAACGAA	13	134	11:1222860	-21.2	AM454228.2			Y
va-miR056-3	TAAAATCACTATCAAACGAA	12	132	14:9946665	-25.85	AM454228.2			
va-miR056-4	TAAAATCACTATCAAACGAA	13	255	16_random:3940513	-39.5	AM454228.2			
va-miR056-5	TAAAATCACTATCAAACGAA	15	136	8:11389633	-33.7	AM454228.2			
va-miR057^b ^-1	GTTGGAAGTCGGTGGGGGACC	7,884	91	19:13455035	-47.9	No		Vv-miR047^c^	Y
va-miR057^b ^-2	GTTGGAAGTCGGTGGGGGACC	7,884	92	19_random:737237	-42.6	No		Vv-miR047^c^	Y
va-miR058	GTAGCATCATCAAGATTCACA	14	108	6:21821956	-46.5	AM439514.2			
va-miR059 ^a^	GGAATCTTGATGATGCTGCAT	18	152	6:5961963	-69.27	No	GQ905588.1; GQ419177.2	Vv-miR038*^c^	Y
va-miR060	GAACATCCAGATCATAGGTATGA	9	276	13:6835040	-49.2	AM470549.2			
va-miR061	CTTCAGTGAGTGTGGCGTCAACG	8	155	1_random:4350301	-56.26	AM424902.2			
va-miR062	CTATGTTATAGGATCTTGGAT	315	99	10:1456140	-51.8	No			Y
va-miR063^b^	CTAGGAAGCGTTTTTAATATT	6	99	8:910651	-38.93	AM439566.1		Vv-miR035^c^	
va-miR064	CTAAAATCATTGTCAAACGAG	12	303	scaffold_352:50742	-54.8	AM449536.2			
va-miR065-1	CTAAAATCACTACCAAACGG	10	99	1:15345941	-23	AM432849.2			
va-miR065-2	CTAAAATCACTACCAAACGG	9	129	7:12853717	-24.2	AM432849.2			
va-miR066	CGTCTTTTAAATTTTTTTATC	6	121	7:11538839	-19.7	No			
va-miR067	CGGGAGATGACTACTGGAAG	8	106	17:2382956	-60.1	No			
va-miR068 ^a^	CGCTATCCATCCTGAGTTTCA	375	122	8:8483671	-54.4	No	AC235430.1; FM163968.1; FM163967.1	Y	
va-miR069	CGCCGCTCTCCTGTGACAAGA	6	92	14:17492727	-57.8	AM479856.1			
va-miR070	CCAAGAGGGTGGAGTTCAGA	10	111	14:13763190	-54.9	No			
va-miR071	CATGTGCCCCTCTTCCCCATC	5	168	8:8981156	-60.21	AM459087.2			
va-miR072	CATGGGCGGTTTGGTAAGAGG	26,555	117	1:3849422	-46.2	No			Y
va-miR073	CATGACAAAAGATACTTCATT	19	95	6:9332049	-64.1	No			
va-miR074	CAGGTTTATTGTTTTTTAATT	7	95	1_random:3034469+	-22	No			
va-miR075	CAGGAAAGTCAGGGAAGTGTA	9	93	13:6656017	-41.93	No			Y
va-miR076	CAAGTGTGGGATTTTGGGTGGCT	14	187	4:15148566	-59.2	AM475853.2			
va-miR077	ATTTCATCATTATATAAAACG	5	260	2:10655532	-124.6	No			
va-miR078	ATTGACATCGTCTAACATAA	27	96	13:908030	-37.2	No			
va-miR079	ATTATTTTTTTTCATGGGAA	7	183	6:4945482	-40.4	AM435008.2			
va-miR080	ATTATATGTGTGAGCCGTTCA	39	97	19:13648253	-23.2	No			
va-miR081	ATCATGTAGATATTGTCCGCT	21	216	18:11023560	-77.4	No			
va-miR082 ^b^	ATATTATTAGGGAATTAAGT	4	82	18:15863697	-19.7	AM482601.2		Vv-miR024^c^	
va-miR083	ATACCATGTGGAAAAGAGGAATC	89	199	16:2126243	-38.7	AM430285.2			
va-miR084	ATAAAACAGGGCATAATTAG	90	162	8:3276798	-27.3	AM455775.2		Vv-miR022^c^	
va-miR085-1	AGGGAAGGTCCCGTTGAGTT	8	87	3:4651805	-34.8	AM432941.2			
va-miR085-2	AGGGAAGGTCCCGTTGAGTT	11	80	scaffold_389:33261	-31	AM432941.2			
va-miR086	AGAGCCAAATATACTCCGCGG	19	83	9:402800	-52.1	AM471736.2			
va-miR087^b ^-1	AGAATGTGTTTGATAGTAATT	7	220	13:10609418	-32.71	AM450684.2; AM470125.2; AM466462.2	Vv-miR013^c^		
va-miR087^b ^-2	AGAATGTGTTTGATAGTAATT	7	152	3:9026582	-32.9	AM450684.2; AM470125.2; AM466462.2	Vv-miR013^c^		
va-miR087^b ^-3	AGAATGTGTTTGATAGTAATT	7	134	5:8615567	-39.8	AM450684.2; AM470125.2; AM466462.2	Vv-miR013^c^		
va-miR087^b ^-4	AGAATGTGTTTGATAGTAATT	7	99	7:6125455	-19.4	AM450684.2; AM470125.2; AM466462.2	Vv-miR013^c^		
va-miR088^a^	AGAAGAGAGAGAGTACAGCTA	27	259	5:17354630	-65.6	No	AL606589.3; AY946858.1	Vv-miR012^c^	
va-miR089	AGAAGAACAAGTAGACTGAGC	11	95	12:17274522	-35.6	AM457953.2			
va-miR090	AGAAAACTGTTTTTAAAAACT	25	209	3:3268552	-33.5	AM428594.2			
va-miR091	AAGTGGTGCTGTCTAGGGTAT	8	153	16_random:2298331	-74.9	AM461957.1			
va-miR092	AAGGTTACGAAGAATGTGAG	7	65	6:20373849	-28.4	AM449474.1			Y
va-miR093 ^b^	AAGGGTTTCTCACAGAGTTTA	40	135	18:1385784	-66.9	AM428484.2		Vv-miR079^c^	
va-miR094 ^b^	AACTAACATAACTAAAGTGAA	21	78	18:7824675	-25	AM444284.2		Vv-miR004^c^	
va-miR095	AACAAATGCTTGATTAAATG	7	185	16:3892094	-46.34	AM456738.2			
va-miR096	AAATTTGATTTTATGGTATT	14	277	1:4778703	-52.3	AM479127.2			
va-miR097	AAATTGGCTCTGTAAATTTC	14	137	18:3503665	-77.6	AM454446.1			Y
va-miR098	AAATTGACCTATTTAATAACT	6	225	13:6487398	-33.8	No			
va-miR099-1	AAATAGAACAAAACACAGCAA	8	101	4:15872785	-57.2	AM424238.2			
va-miR099-2	AAATAGAACAAAACACAGCAA	8	101	scaffold_477:40535	-63.2	AM424238.2			
va-miR100	AAAGTGCATTTGATAGTGATTC	5	142	19_random:164412	-29.22	AM481492.2			Y
va-miR101	AAAAATGAAAATGGGAGTCGGT	11	243	5:12357599	-42.41	AM466362.2			
va-miR102 ^b^	AAAAAGTTTGTCAAATATTG	15	97	19:13648253	-23.2	AM435471.2		Vv-miR001^c^	
va-miR103	AAAAAATTCAAAGGGAAATC	8	162	2:2995078	-26.63	AM473477.2			
va-miR104 ^b^	GGAATGGATGGTTAGGAGAG	2,136		17:5578270	-54.4	AM458290.1		MirC13^d^	Y
va-miR105	TAAAAGATAGGGATCGGATGGAG	6	192	14:15112983	-59.2	AM463372.2			
va-miR106	GTTGGAAGTCGGTGGGGGACC	4,330	91	19:13455035	-47.9	AM481325.2			

Notes: miRNAs with ^a ^and ^b ^represent non-conserved miRNAs, whereby those with a were miRNAs specific to a few plants species, while ones with ^b ^were specific to plants in the vitis genus; ^c ^denote miRNAs validated by Wang *et al. *(2011); ^d ^denote miRNAs validated by Pantaleo *et al. *(2010).

**Figure 2:**
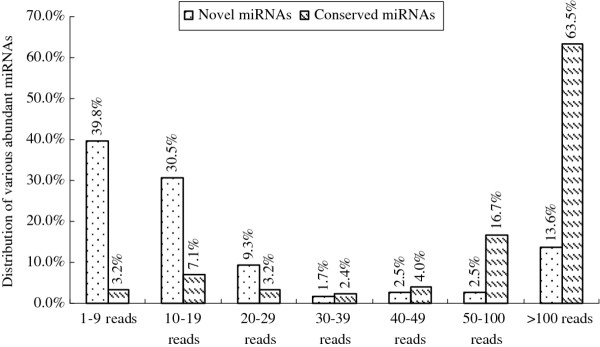
**3'RACE and 5' RACE PCR products of seven amplified va-miR-SNPs shown in an ethidium bromide-stained agarose gel**. Sizes of the molecular weight markers of the bottom and the top bands are 50 bp and 100 bp, respectively. Lanes 1-7, 1'-7' are 3'RACE (up) and 5'RACE (down) products of va-miR156a-SNP, va-miR166a-SNP, va-miR166h-SNP, va-miR169b-SNP, va-miR169l-SNP, va-miR169o-SNP and va-miR171c/d-SNP, respectively. The sizes of 3'RACE products are about 83 bp while the size of 5'RACE products are about 57 bp.

In addition, it was revealed that the read number for a majority of these new va-miRNAs were much lower than those for the conserved miRNAs (Figure 2), consistent with previous conclusions [10,13-16], indicating that new non-conserved miRNAs are usually expressed at lower levels and have some species-specific regulatory roles during growth and development of Amur grape.

### Validation of Amur grape miRNAs

The abundance of different miRNAs in an organism varies with the levels of expression, which could influence their read numbers during sequencing. Theoretically, the higher the expression of miRNAs in organisms, the more the reads that can be sequenced and the higher the degree of accuracy in acquiring sequences during high throughput sequencing. This suggests the need for validation of the sequencing quality of new miRNAs having low expression levels. The obvious differences in the copies of miRNAs sequenced and the large number of low copy miRNAs in this study suggest the need for validation. This validation can be readily done using a specific PCR and PCR product sequencing, for which miR-RACE can be prefered method due to its effectiveness in the validation of precise sequences, especially both ends of miRNAs [19,36]. In this study, we performed validation of precise sequences of 20 low abundance va-miRNAs (< 10 reads) and 20 high abundance (> 100 reads) va-miRNAs selected randomly using miR-RACE. The 20 low abundance va-miRNAs included 17 new and 3 conserved ones (e.g. va-miR398b, va-mir399c and va-miR828a), while the high abundance group included 10 conserved and 10 new va-miRNAs. miR-RACE results demonstrate that all these 40 potential va-miRNA sequences could be clearly amplified in the 3'- miR-RACE and 5'-miR-RACE analysis (Additional file 4). The miR-RACE products were cloned and sequenced for confirmation where identical results were generated by both high throughput sequencing and miR-RACE, thus further confirming the power of Solexa technology in sequencing sRNA libraries for miRNA discovery as well as ascertaining the existence of these miRNAs.

### Evolutionary conservation of miRNAs in Amur grape

To study the conservation and evolutionary roles of these identified va-miRNAs, we conducted two aspects of comparative analysis for both conserved and new va-miRNAs. On one hand, extensive comparison between the identified va-miRNAs and the known miRNAs in 28 other plant species (including *Vitis vinifera*) from miRbase 16.0 were performed to investigate evolution of the identified conserved va-miRNAs (Table 5). On the other hand, all 106 va-miRNAs identified as potentialy new were used as query sequences to perform Blastn searches against all nucleotide sequences in NCBI databases for conservation analysis of these new miRNAs in a wide range of plant species.

**Table 5:** Analysis of orthologs of conserved miRNAs in 28 plant species

Plant name	miR156	miR159	miR160	miR162	miR164	miR166	miR167	miR168	miR169	miR171	miR172	miR319	miR390	miR393	miR394	miR395	miR396	miR397	miR398	miR399	miR403	miR408	miR477	miR479	miR482	miR535	miR828	miR845	miR 2111
P01	+	+	+	+	+	+	+	+	+	+	+	+	+	+	+	+	+	+	+	+	+	+	+	+	+	+	+	-	-
P02	+	+	+	+	+	+	+	+	+	+	+	+	+	+	+	+	+	+	+	+	+	+	+	+	+	+	+	+	+
P03	+	+	+	+	+	+	+	+	+	+	+	+	+	+	+	+	+	+	+	+	+	+	-	-	-	-	+	-	+
P04	+	+	+	-	+	+	+	+	+	+	+	+	+	+	+	+	+	+	+	+	-	+	-	-	-	+	-	-	-
P05	+	+	+	-	+	+	+	+	+	+	+	+	+	+	+	+	+	-	-	+	-	+	-	-	+	-	-	-	-
P06	+	+	+	-	+	+	+	+	+	+	+	+	+	+	+	+	+	+	-	+	-	+	-	-	-	-	-	-	-
P07	+	+	+	+	+	+	+	+	+	+	+	+	+	+	-	+	+	-	+	+	-	-	-	-	-	-	-	-	+
P08	+	+	-	-	-	-	-	+	-	-	-	-	-	-	-	-	+	-	-	-	-	+	-	-	-	-	-	-	-
P09	+	+	+	+	+	+	+	+	+	+	+	+	+	+	-	-	+	-	+	-	-	-	-	-	+	-	-	-	-
P10	+	+	+	+	+	+	+	+	+	+	+	+	+	+	+	+	+	+	+	+	+	+	+	+	+	-	-	-	-
P11	+	-	+	-	-	+	-	-	-	+	-	+	+	-	-	-	-	-	-	-	-	-	+	-	-	+	-	-	-
P12	+	-	-	+	+	+	+	-	-	-	+	-	+	+	+	-	+	-	+	+	-	-	-	+	+	-	-	-	-
P13	-	-	-	-	-	-	-	-	-	-	-	-	-	-	-	-	-	-	-	-	-	-	-	-	-	-	-	-	-
P14	+	+	+	-	+	+	+	+	+	+	+	-	+	+	-	-	+	+	+	+	-	-	-	-	-	-	-	-	+
P15	+	+	-	-	-	+	-	-	-	-	-	+	+	-	-	-	+	-	+	-	-	-	-	-	+	-	-	-	-
P16	+	+	+	-	-	+	-	-	-	+	-	+	-	-	-	-	+	-	-	-	-	-	-	-	-	-	-	-	-
P17	+	+	+	-	+	-	+	-	-	+	-	+	-	-	-	-	-	-	-	+	-	+	-	-	-	-	-	-	-
P18	-	-	-	+	-	-	-	-	-	-	-	-	-	-	-	-	-	-	-	-	-	-	-	-	-	-	-	-	-
P19	+	+	+	+	-	+	+	-	+	+	+	+	-	-	-	+	-	+	-	-	-	-	-	-	+	-	-	-	-
P20	-	-	-	-	-	-	+	-	-	-	-	-	-	-	-	-	+	-	-	-	-	-	-	-	-	-	-	-	+
P21	-	-	-	-	-	-	-	-	-	-	-	-	-	-	-	-	-	-	-	-	-	-	-	-	-	-	-	-	-
P22	-	-	-	-	-	-	-	-	-	-	-	-	-	-	-	-	-	-	-	-	-	-	-	-	+	-	-	-	-
P23	-	+	-	-	-	+	-	-	-	-	-	+	-	-	-	-	-	-	-	+	-	-	-	-	+	-	-	-	-
P24	+	-	-	-	-	+	+	-	+	+	-	+	-	-	-	-	-	+	-	+	-	-	-	-	-	-	-	-	-
P25	+	+	+	-	-	+	-	-	+	+	+	+	-	-	-	+	+	-	+	+	-	-	+	-	+	+	-	-	-
P26	+	+	+	+	+	+	+	+	-	+	+	+	+	-	-	-	+	-	+	-	-	-	-	+	+	-	-	-	-
P27	+	+	+	+	+	+	+	+	+	+	+	+	+	+	-	+	+	+	+	+	+	+	-	-	-	+	-	-	-
P28	+	+	+	-	-	-	+	-	-	-	-	-	-	-	-	-	-	-	+	-	-	+	-	-	-	-	-	-	-

Notes: P01, *Vitis amurensis Rupr*.; P02, *Vitis vinifera*; P03, *Arabidopsis thaliana*; P04, *Oryza sativa*; P05, *Zea mays*; P06, *Sorghum bicolor*; P07, *Medicago truncatula*; P08, *Saccharum spp*.; P10, *Glycine max*; P10, *Populus trichocarpa*; P11, *Physcomitrella patens*; P12, *Gossypium hirsutum*; P13, *Chlamydomonas reinhardtii*; P14, *Brassica napus*; P15, *Pinus taeda*; P16, *Selaginella moellendorffii*; P17, *Triticum aestivum*; P18, *Carica papaya*; P19, *Solanum lycopersicum*; P20, *Lotus japonicus*; P21, *Vigna unguiculata*; P22, *Malus domestica*; P23, *Phaseolus vulgaris*; P24, *Brachypodium distachyon*; P25, *Aquilegia caerulea*; P26, *Citrus sinensis*; P27, *Ricinus communis*; P28, *Arachis hypogaea*; + with orthologs, - without orthologs.

Among the conserved miRNA sequences obtained from Amur grape, four miRNA families (va-miR403, va-miR477, va-miR479 and va-miR828) showed conservation in no more than five of the plant species investigated, with miR828 being found in only the *Vitis *and *Arabidopsis *genera, indicating that the four miRNA families have specificity in a narrow range of plants. As expected, Amur grape and *Vitis vinifera *shared 27 conserved miRNAs families, exhibiting higher conservation of miRNA families in these two grapevine species, which is also consistent with their closer genetic relationship. Nevertheless, vv-miR845 and vv-miR2111 in *Vitis vinifera *have no orthologs in Amur grape with vv-miR845 being unique to *Vitis Vinifera*, confirming the need for studies on Amur grape miRNAs for a comprehensive understanding of functions of miRNAs in vitis plants. We also discovered that apart from *Vitis vinifera*, va-miRNAs were much more conserved with their orthologs in poplar than those in the other species investigated, with a total of 25 miRNA families being conserved between Amur grape and poplar; while there were no orthologous miRNA families in Amur grape, *Chlamydomonas reinhardtii *and *Vigna unguiculata*. This phenomenon indicates that speciation of plants was accompanied with the specialization of miRNAs playing species-specific roles.

To further analyze the sequence evolutionary levels of 106 potential new va-miRNAs, these miRNAs were used as queries to search against the nucleotide sequence database in NCBI. The analysis results in Table 4 reveal that 96 of these va-miRNAs could not be found in any other plant species other than in *Vitis vinifera*, while only ten va-miRNAs (va-miR001, va-miR006, va-miR008, va-miR010, va-miR018, va-miR027, va-miR047, va-miR059, va-miR068 and va-miR088) have homologs in other plant species. Further comparison of the 96 va-miRNAs with vv-miRNAs identified from other wine and table grape cultivars showed that 72 va-miRNAs were exclusive to Amur grape, an indication that they could be Amur grape-specific and might possess some specific regulatory functions during Amur grape growth and development. The other 24 could be considered as vitis-specific due to their existence only in plants from the vitis genus.

### Analysis of va-miRNA SNP data from deep sequencing

Since miRNAs are small functional units, single nucleotide shifts in the precursor elements as well as the mature miRNA sequences may drive the evolution of new miRNAs by altering their biological functions [37]. Furthermore, sequence variation around the processing sites, and sequence variations in the mature miRNA, particularly the seed sequence, may have profound affects on miRNA biogenesis and functions [37]. Uncovering and characterization of the single base substitution and length differences between wild type and variant sequences of conserved miRNAs can be quite helpful in the investigation of miRNA evolution within a plant species and/or across species. We reveal that out of the 126 conserved va-miRNAs identified in this study, 70 were only sequenced in their wild type sequences and their read numbers varied widely from 2 to 2337, while the remaining 56 conserved miRNAs were detected in both the variant as well as wild type sequences, with copies of most of the former being much less than copies of the later. Similarly, we also identified a number of single base variants of miRNAs in the *Fragaria vesca *small RNA library (data not shown), indicating that this phenomenon may be not an occasional event derived from sequencing errors, miRNA SNPs might do exist in small RNA library. As reported in human liver miRNAs [38], these variants also included single nucleotide polymorphism (SNP) and/or length difference. To elucidate the characteristics of these miRNA variant phenomena, SNPs occurring in mature miRNA sequences were considered as miR-SNPs [37], while length differences of miRNAs were described as miR-LDs. In total, 43 kinds of miR-LDs and 117 types of miR-SNPs were identified from 43 and 56 conserved va-miRNAs, respectively (Additional file 5 and Additional file 6). Even though the number of miR-SNP types is greater than that of miR-LDs, the read number of miR-SNP is lower than that of miR-LDs, and the read numbers of both are far lower than in the corresponding wild types. In order to further validate the existence of these variant phenomena, some sequences with variant bases were randomly selected and subjected to experimental verification. Here, miR-RACE was utilized to verify existence of ten miR-LDs, among which seven were successfully validated (Figure 1). These results to some extent confirm the reality and existence of variant phenomena (miR-SNPs, miR-LDs etc.) in sequences during miRNAs evolution.

**Figure 3:**
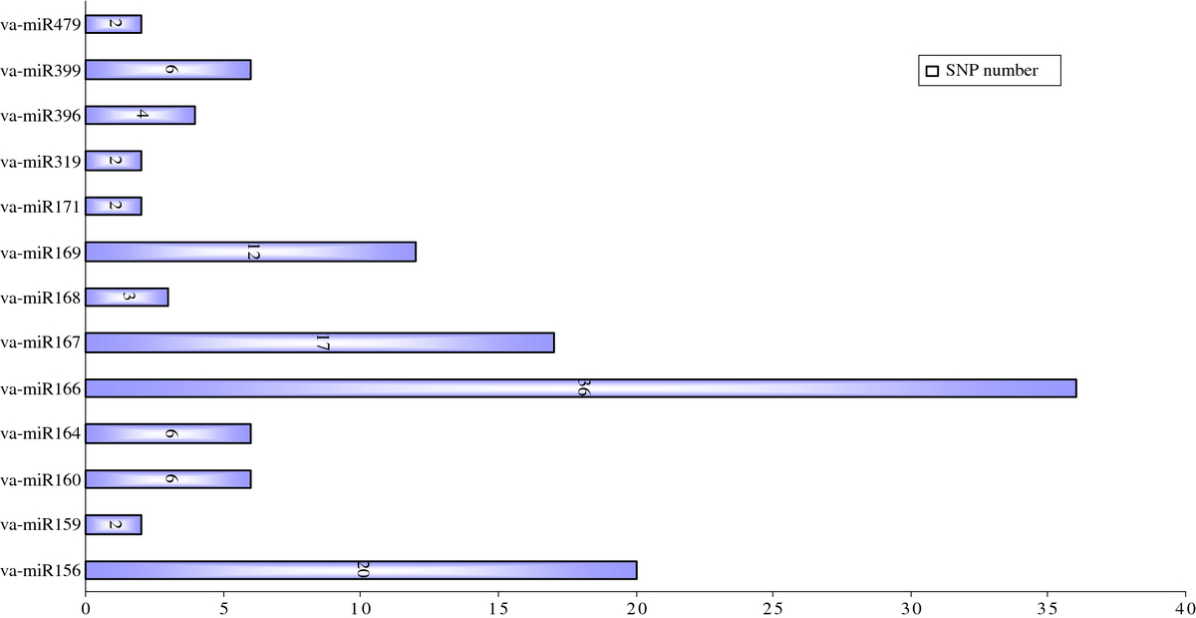
**Number distribution of miR-SNP of diverse conserved miRNAs familes in Amur grape**.

**Figure 4:**
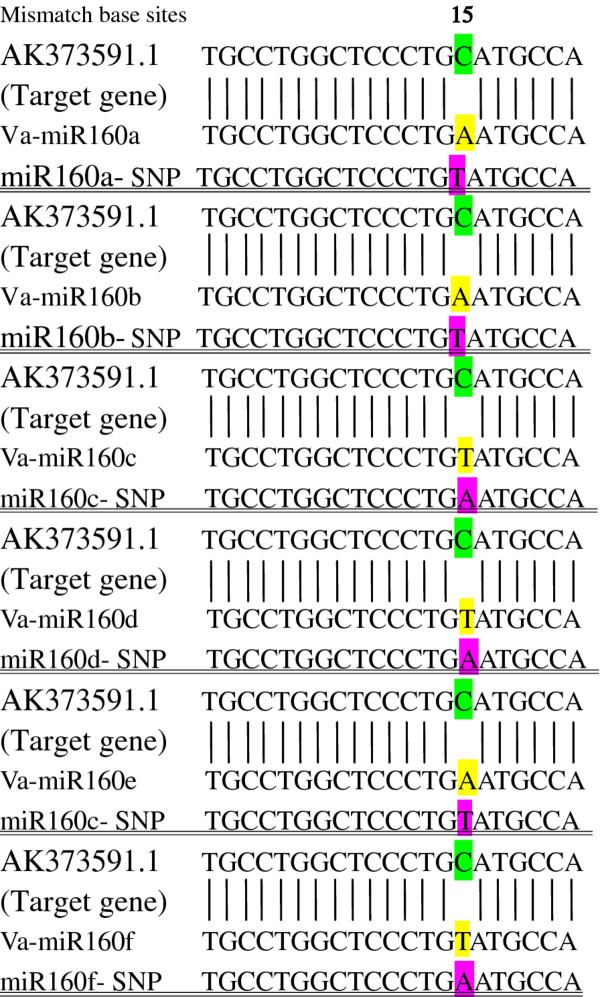
**Comparison of polymorphisms of va-miR160a, b, c, d, e and AK373591.1 binding sites, respectively**. Va-miR160a, va-miR160b, va-miR160c, va-miR160d, va-miR160e respectively showed wild type sequences; miR160a-SNP, miR160b-SNP, miR160c-SNP, miR160d-SNP, miR160e-SNP exhibited SNP types of these miRNAs.

It should be noted that only one miR-LD could be detected in each conserved va-miRNA, while one to five types of miR-SNPs were found in the same va-miRNA, suggesting that sequence variations in seed sequences of miRNAs may be among important factors driving the evolution of new miRNAs, even though their abundance is much lower than that of wild types. The numbers of miR-SNPs located in different members of the same miRNA family and those in diverse miRNA families were all distinctly different. Although the va-miR169 family had the highest number of miRNA members with SNPs, the va-miR166 family topped in the list of total number of SNPs, followed by va-miR156, va-miR167 and va-miR169 families (Figure 3). The number of SNPs in each member of the other miRNA families was lower than six. These situations suggest potential divergence in conservation during evolution of va-miRNAs. In addition, SNP sites in different members of some va-miRNA families were identical or quite similar, which can be attributed to all members of each group targeting identical or similar genes, making diverse members of the same miRNA family exhibit functional conservation. The best example of this is the va-miR160 family, where nucleotide variants of all its six members (a, b, c, d, e, f) not only exist in the middle of their mature sequences, but all have an identical miRNA SNP site. Since this site was located at a miRNA-mRNA mismatch position as well as at a discrepancy site in diverse members (Figure 4), this miR-SNP in the va-miR160 family is unlikely to change the target genes of these miRNAs. The SNP sites of different members in other va-miRNA families were clearly divergent, with most of the SNPs located in mature va-miRNAs sequences seeming to increase the mismatch positions of miRNA-mRNA binding which could result in changes in their targeting patterns or losing target roles, and also become a potential cause for generation of new miRNAs. The best example of this phenomenon is the location of SNPs in the va-miR156 family, where 15 of the 20 SNPs created new mismatch sites from the match position upon which miRNAs bind their target genes, while the variant bases of the other five SNPs occurred in the original mismatch positions of miRNAs and the binding regions in their target genes. This suggests that nucleotide variation within these miRNA families is likely to be one of the important driving powers in the evolution of new miRNAs, consistent with that reported in miRNAs of *Arabidopsis *and humans [37-39]. Compared to *Arabidopsis *and humans, these miR-SNPs were less abundant in Amur grape. The various characteristics of SNP sites from the two miRNA families (va-miR160 and va-miR156) above suggest that significant divergence exists in miRNA conservation and evolution among different miRNA families.

### Analysis of the expression profiles of va-miRNAs

**Figure 5:**
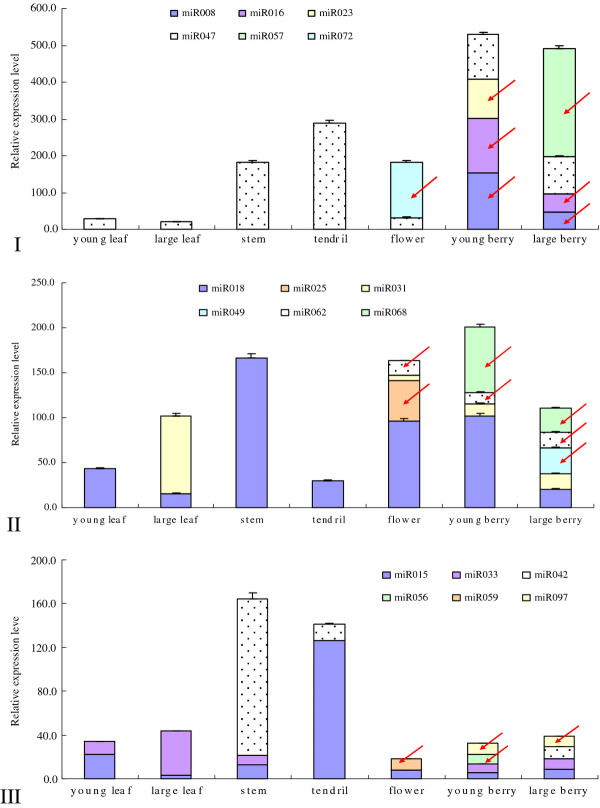
**Real-time RT-PCR expression profiles of va-miRNAs with three frequencies from deep sequencing sRNA library in seven tissues of Amur grape**. I, II, III denote va-miRNAs with high, moderate and low frequency in the deep sequenced sRNA library, respectively; the red arrows denote the flower- and/or berry-specific miRNAs. Lanes: young leaf (4 cm in diameter), large leaf (8 cm in diameter), stem (0.2 cm in diameter), tendril (0.1 cm in diameter), inflorescence (0.2 cm in diameter per grain), flower (0.25 cm in diameter per grain), young berry (0.8 cm in diameter) and large berry (1.5 cm in diameter). Each reaction was repeated three times and the template amount was corrected by 5.8 s rRNAs.

High throughput sequencing can provide an efficient method for the detection of expression profiles of miRNAs as well as the identification of miRNA sequences. Usually, highly expressed miRNAs can be sequenced at high rates and a large number of their reads can be generated. Results from Solexa sequencing exhibit drastically different expression levels of members of different miRNAs families [12,14,18,25,28]. Abundance comparison of similar or different miRNAs in various tissues could present valuable information for better understanding of the roles of miRNAs. Based on the diverse abundance of miRNAs in our Amur grape sRNAs library, three groups of new va-miRNAs were randomly selected and aptly named Groups I, II and III. Group I comprised highly abundant va-miRNAs with over 4,000 reads, Group II had moderately abundant va-miRNAs with 100-4,000 reads while Group III had lowly abundant va-miRNAs with < 100 reads. To study spatiotemporal expression of the new va-miRNAs having diverse copies and obtained by deep sequencing of the mixed sRNA library of different organs and tissues, six va-miRNAs per group (Figure 5) were randomly selected and their expression profiles in young leaves, large leaves, stems, tendrils, flowers, young berries and large berries analyzed with real-time PCR (qRT-PCR). The qRT-PCR expression results (Figure 5) show that even though the qRT-PCR result of va-miRNAs in flowers and berries at different developing stages could not be compared well with those data from deep sequencing the combined sRNA libraries, totally, qRT-PCR expression levels of va-miRNAs from flowers and berries at various developing stages were relatively consistent with those shown as read numbers from deep sequencing sRNAs libraries of combined corresponding tissues, confirming that deep sequencing can accurately detect accumulation levels of miRNAs in organisms. As shown in Figure 5, the qRT-PCR expression profiles of va-miRNAs in young and large leaves, stems, tendrils, inflorescences, flowers and berries have significant differences and can thus demonstrate the existence of spatiotemporal specificity in miRNA expression levels, an observation that could provide important insight to functions of va-miRNAs. Further analysis on the expression modes of 18 va-miRNAs in the three groups reveals that 13 va-miRNAs were expressed in flower and/or berry tissues of Amur grape. Eight va-miRNAs were only expressed in single tissues investigated, but their accumulation levels exhibited clear differences as exemplified by va-miR025, va-miR059 and va-miR072 in flowers, va-miR023 and va-miR056 in young berries, va-miR056, va-miR057 and va-miR069 in large berries, and va-miR008, va-miR016, va-miR068 and va-miR097 in both young and large berries. One va-miRNA (va-miR031) was expressed only in reproductive organs (flowers and berries) (Figure 5). These observations indicate that expression characteristics of most new va-miRNAs in this study were flower- and/or berry-specific, an aspect attributed to our small RNA library coming from a mixture of flower and berry tissues. It was also observed that va-miR015, va-miR018 and va-miR047 were ubiquitously expressed but at varying levels in all organs studied. These phenomena indicate that the extent/strength of miRNA regulatory roles during various development stages of Amur grape might have significant divergence. The other two va-miRNAs (va-miR033 and va-miR042) showed another kind of expression pattern where they were expressed in most but not all of the tissues investigated. All these findings point to typical spatiotemporal specificity of these new va-miRNAs. Generally, qRT-PCR expression profiles not only confirmed the existence of these new miRNAs in Amur grape, but also suggest that these va-miRNAs possess tissue- or developmental stages-specificity, which can in turn lay a solid foundation for studies on detailed functions of va-miRNAs.

### Prediction of va-miRNA target genes

The prediction of miRNA target genes is important in comprehensive studies of miRNA gene-regulation functions. Currently, bioinformatics methods based on the high homology between miRNAs and target genes are considered one of the most efficient approaches used to verify target genes, and has been confirmed in quite a number of studies [40-43]. To better understand the functions of va-miRNAs, their putative targets were predicted using a BLASTN algorithm based on the homology between miRNAs and their target genes in grapevines following the rules of target prediction put forward by Allen *et al. *[44].

All conserved va-miRNAs (denoted as Category-I va-miRNAs) identified in Amur grape had their homologs in 'Summer Black', a hybrid of *V. vinifera *and *V. labrusca*, deposited and assigned code No. GEO: GSE24531 [23], with the target genes of these homologous miRNAs have been already reported [27]. We further investigated target genes for only the 34 non-conserved and 72 new miRNAs (denoted as Category-II miRNAs). Typically, most non-conserved miRNAs are species-specific and they are often used to give an insight into their orthologous functions in a few plants, thus making prediction of target genes for non-conserved miRNAs a key preliminary work. In this study, a total of 346 targets for 54 out of all 106 Category-II miRNAs were predicted, with a majority (257) being implicated in a broad range of physiological processes, except for 89 whose functions were unknown (Additional file 7). The number of predicted targets per va-miRNA in Category-II varied drastically from 1 to 103 (Additional file 7). Thirty-four of these va-miRNAs had multiple potential target sites, for instance, va-miR006 which has 103 target sites tops the list, followed by va-miR081 and va-miR003 with 59 and 33 target sites respectively. The other 21 va-miRNAs were predicted to have one target gene each, an indication that they might possess unique functions in regulation of growth and development or response to environmental stress by Amur grape. Besides this, we also observed a trend where multiple members of Category-II va-miRNAs targeted the same single gene and thus had similar functions, but it was not clear whether they co-regulated or independently targeted their target gene spatiotemporally, hence necessitating further investigation. No targets could be predicted for the remaining 52 Category- II miRNAs (Additional file 7), which could be attributed to the limited number of grape EST sequences available in the databases and the difference in grape species used in this work and those used in ESTs sequencing.

To elucidate the functions of Category-II va-miRNAs, target genes with functions annotated for 72 new va-miRNAs were first analyzed and classified into four groups. The first group was involved in stress resistance, for example, disease resistance (NB-ARC, NBS-LRR, TIR-NBS-LRR, BZIP, EFR (EF-TU RECEPTOR), RPA1, EDM2, WRKY, GRAM, CBS, IBS1 and TGA1) and abiotic i.e. cold, drought, salt and metal iron stress resistance (HVA22 I, TPR repeat-containing protein, ABRH23, protein RER1B, lactoylglutathione lyase, SPX (SYG1/Pho81/XPR1) domain-containing protein, and aluminum-activated malate transporter). This gives an indication that roles for these potential new miRNAs are comprehensively in the regulation of biotic and abiotic stress responses especially disease, cold and drought resistance, and explains why Amur grape is one of the most important wild grape germplasm with relatively strong stress tolerance. The second group was implicated in several hormone signalling pathways (AUX/IAA, gibberellin, cytokinin, ethylene), calcium, apoptosis, G-protein and membrane protein signals and include the Aux/IAA like protein, two-component response regulator ARR8, AIL6, scarecrow gene regulator, ankyrin repeat family protein, Copine III, TOM (Target of myb1), GTPase, GRAM domain containing protein and integral membrane protein, thus indicating that these new va-miRNAs might participate in important biological pathways. The third group was related to synthesis metabolisms like anthocyanin synthesis (WD40, WD repeat protein, Cytochrome b5 DIF-F and TTG1), sugar (UDP-glucose: glycoprotein, glucose) synthesis (vacuolar invertase 1 (GIN1) (hexose), and rhamnose biosynthetic enzyme 1 (rhamnose)), showing that target genes for this group of new va-miRNAs may be involved in berry coloration and flavor quality formation. The last group of target genes could control flower development, and include FCA gamma, Isoform 2 of AP2-like ethylene-responsive transcription factor AIL6, vernalization independence 4 and scarecrow gene. Interestingly, all the above target genes (LIM, WD, AIL6, GIF1, TGA1.1, WRKY, GRAM, MYB127, BZIP, AUX/IAA and PHD) were also important transcript factors or regulator like (Additional file 7). Therefore, these new Amur-specific miRNAs might play far much more significant roles in regulation of growth, development and response to stress of Amur grape.

Among the 34 non-conserved miRNAs, 14 had no target genes, while another 20 were predicted to target 45 genes, out of which 10 had unknown functions. The functions of the other 35 target genes include regulation of metabolic enzymes (Type IIB calcium ATPase, E3 ubiquitin-protein ligase SINAT2, carboxylesterase, oxidoreductase), growth development process (Cellulose synthase D4), stress responses (LRRNT_2 domain containing protein and TPR repeat-containing protein), signal transduction (Type IIB calcium ATPase and WD-repeat membrane protein) and as important transcript factors related to flower development (Transcription factor AP2D23, PHAP2A protein, PHAP2B protein and Squamosa promoter binding protein-homologue 5). These findings are consistent with those of previous reports on different vitis species [20-24] and other plant species [11,14-16,34].

### Verification of potential va-miRNA target genes using 5'-RLM-RACE

**Figure 6:**
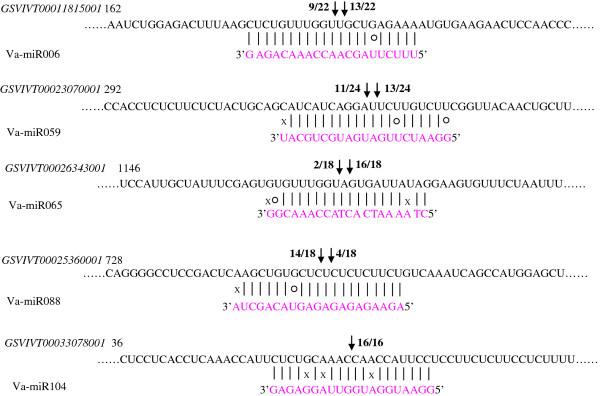
**Mapping of mRNA cleavage sites by RNA ligase-mediated 5' RACE**. Each top strand (black) depicts a miRNA-complementary site in the target mRNA, and each bottom strand (pink) depicts the miRNA. Watson-Crick pairing (vertical dashes), G:U wobble pairing (circles) and mismatched bases pairing (X) are indicated. Arrows indicate the 5' termini of mRNA fragments isolated from Amur grape, as identified by cloned 5'RACE products, with the frequency of clones shown. Only cloned sequences that matched the correct gene and had 5' ends within a 100 nt window centered on the miRNA complementary site are counted. Partial mRNA sequences from target genes were aligned with the miRNAs. Numbers indicate the fraction of cloned PCR products terminating at different positions.

To verify the nature of potential miRNA targets and to study how the va-miRNAs regulate their target genes, a modified RLM-RACE experiment was set up. The RLM-RACE procedure was successfully used to map the cleavage sites in five predicted va-miRNA target genes. *GSVIVT00011815001*, *GSVIVT00023070001*, *GSVIVT00026343001*, *GSVIVT00025360001 *and *GSVIVT00033078001 *were confirmed as the real targets of va-miR006, va-miR059, va-miR065, va-miR088, and va-miR104 respectively since all the 5'-ends in the mRNA fragments mapped to the nucleotide that pairs to the tenth or ninth nucleotide of each miRNA with higher frequencies than depicted for each pairing oligo (Figure 6). All five predicted targets were found to have specific cleavage sites corresponding to the miRNA complementary sequences (Figure 6) and might be regulated by the five va-miRNAs. *GSVIVT00011815001*, *GSVIVT00023070001*, *GSVIVT00026343001*, *GSVIVT00025360001*, *GSVIVT00033078001 *are similar to Isoform 2 of AP2-like ethylene-responsive transcription factor AIL6, PHAP2B protein transcription factor, NAD-dependent epimerase/dehydratase, Squamosa promoter binding and Betv I domain containing protein (Additional file 7).

## Discussion

Although several studies on grape miRNAs have recently been reported [20-24], the grape materials used in all the studies reported belong to the cultivated grape *Vitis vinifera *or hybrids of *V. vinifera *and *V. labrusca*. Amur grape (*Vitis amurensis Rupr*.) is one of the most important wild species in the grape family. Currently, only a few genes involved in resistance to downy mildew have been identified in Amur grape [45] and there are no known reports on Amur grape miRNAs. The identification of entire sets of miRNAs and their targets will lay a foundation for elucidation of the complex miRNA-mediated regulatory systems which control development and other physiological processes [4,46]. Systematic studies on miRNAs in Amur grape will contribute to gaining insight into the mechanisms controlling growth and development in this grape species. The advent of deep sequencing technologies has greatly enhanced the capacity of sRNA exploration, and in turn provided a rapid way to identify non-conserved, low accumulation, species-specific as well as conserved miRNAs on a large scale. In this study, using Solexa sequencing, we provide evidence supporting the existence of 106 new and potential, as well as 126 conserved miRNAs in Amur grape. Use of deep sequencing technology, has led to identification of many new grape miRNAs in *Vitis vinifera *[20,21] or hybrids of *V. vinifera *and *V. labrusca *[23,24], and ITS employment in our study led to the identification of 72 additional miRNAs that are specific to the Amur grape species. Future large scale experimental approaches in more plants are likely to identify additional species-specific miRNAs.

The aim of this work was to identify miRNAs present in Amur grape including miRNAs evolutionary conserved in other plants. Searches for conserved miRNAs revealed that many va-miRNAs have orthologs in other plants like *Arabidopsis *[28], rice [14], poplar [15], *Medicago *[47], *Solanum lycopersicum *Mill [12], peanuts [17], citrus [43], wheat [33] etc. In addition, deep sequencing of the small RNA library allowed for identification of the expression levels of each member of a miRNA family. Sequence analysis reveals that the relative abundance of certain members within the miRNA families varied drastically, from 2 to 372,442 copies. Amongst the conserved miRNAs, va-miR166h topped the list in terms of copy numbers, but the annotation of its function is still not available, consistent with the observation in Summer Black grapevine which is a hybrid of *V. vinifera *and *V. labrusca *[23,24]. Conversely, va-miR171h, va-miR169i and va-miR159a/b appeared to have only less than 10 copies each (Figure 2). We also discovered that these conserved miRNAs have their orthologs in nearly 30 other plant species/varieties (Table 5), as indicated in previous miRNA reports [48-52]. Some miRNAs are conserved in several plant species, an aspect which will provide an opportunity for assessment of evolution of these families across diverse plants. Seventy two new and potential va-miRNAs were specific to Amur grape and were not detected in other vitis species; while another twenty-four were only conserved in *Vitis amurensis *Rupr. and *Vitis vinifera *L. These open the possibility that these miRNA families could have descended from a common ancestor and diverged or were lost during evolution of vitis plants. It is also possible that regulatory interactions directed by these vitis-specific miRNAs are involved in the adaptation to the diverse ecological environments.

Since the roles of miRNAs in development and biotic or abiotic stress regulation are executed through the cleavage or translation repression of target genes, miRNA target prediction is critical for gaining insight to regulatory functions of miRNAs. Before this report, target genes for conserved miRNAs in grape had been systematically investigated in other vitis species [20,21,23,24,53]. Conserved miRNAs have high sequence conservation in closely related plant species and thus their target genes also possess some conservation of functions. For non-conserved and new or potential va-miRNAs, we reveal that a number of disease resistance, stress resistance, anthocyanin synthesis regulatory genes as well as some other genes related to secondary metabolism and sugar metabolism might be targeted by va-miRNAs based on previous reports on functional annotation of orthologous genes in other plants [17,41,54-56]. Outstandingly, we found 11 important transcript factors and regulators for va-miRNAs, and these might play a wide range of vital roles during development or response to stress in Amur grape. Other target genes were associated with signal transduction, metabolism, transport, growth and development processes, which is similar to studies by Wu *et al. *[45] in the Amur grape DNA library. All these observations indicate that both the new and non-conserved va-miRNAs identified in this study might play extensive regulatory roles, not only in development, but also in stress response and diverse physiological processes.

The availability of a full grape genome and plenty of grape ESTs helped us to identify 72 new, 34 non-conserved va-miRNAs and 346 target genes. It has been reported that target prediction for new miRNAs may yield some false positives [57]. However, blast search using miRNA sequences against EST databases can also be a good way of identifying potential targets of the new miRNAs, followed by necessary experimental verification. In this work, we employed the 5'-RLM-RACE method to detect miRNA-guided cleavage of target mRNAs of five Va-miRNAs, where the results showed that five potential target genes for the five Va-miRNAs had specific cleavage sites corresponding to their miRNA complementary sequences. This confirms the actual existence of these potential va-miRNA target genes. For a thorough investigation of the real existence of all the potential target genes, systematic research should be planned in the next phase of this work. In addition, there is no current information available regarding the functions of more than 1/4 of the target genes identified, which makes it difficult to determine whether these miRNA targets have functional bias. Furthermore, it was also observed that consistent with previous reports [55-57], most va-miRNA targets have a miRNA-complementary site located in their coding regions and occasionally in their 3' or 5'UTRs. Previous reports indicate that plants usually have a lower number of genes targeted by a single miRNA than in animals. For instance, each drosophila miRNA has on average over 50 predicted targets [58], while most *Arabidopsis *miRNAs have six targets or fewer [4]. In contrast, some va-miRNAs in this study have more target genes than those reported for drosophila and even for *Arabidopsis*, for example va-miR006 which has 103 target genes, a finding that might imply that Amur grape-specific miRNAs have more extensive functions. This study pioneers the first large scale cloning and characterization of Amur grape miRNAs and their predicted targets thus laying a foundation for future functional studies.

## Conclusion

Deep sequencing of short RNAs from Amur grape flowers and fruits identified 72 new potential miRNAs and 34 known but non-conserved miRNAs, indicating that specific miRNAs exist in Amur grape. These results show that a number of regulatory miRNAs exist in Amur grape and they play an important role in Amur grape growth, development, and response to abiotic or biotic stresses.

## Methods

### Plant material

Leaves, stem (0.3 cm in diameter), tendrils, inflorescences, flowers, and developing fruits (15, 30 and 45 days after full blooming) were collected in 2010 from Amur grape (*Vitis amurensis *Rupr.) trees grown under general site conditions at the Fruit Germplasm Resources Garden of the Zhengzhou Institute of Pomology, Chinese Academy of Agricultural Sciences, Zhengzhou, China. After collection, all the samples were immediately frozen in liquid nitrogen and stored at -80°C until use.

### Low molecular RNA extraction

Total RNA was isolated from 200 mg of the selected plant tissues using the CTAB method [59], then 10 M LiCl was used to separate the low and larger molecular weight RNA following the procedures reported earlier [17]. The small RNA fraction was then dissolved in 30 μl of RNase free water and the concentration of RNA measured by a UV-1800 spectrophotometer (Shimadzu, Japan) and visually ascertained in a 2.5% agarose gel. The larger molecular weight RNA samples were used to study the expression patterns of the target genes of va-miRNAs.

### Construction and screening of cDNA libraries of small RNAs

We generated the miRNA-enriched library that has been popularly used to clone miRNAs and to measure the expression of miRNAs via RT-PCR, in which 5'- and 3'-end adaptors were linked to the miRNA molecules [36], which were further reverse transcribed using Superscript III reverse transcriptase (Invitrogen) in the presence of random nonamers (Sigma), according to the protocols provided by the manufacturers. After the preparation of miRNA libraries from various organs and tissues, we pooled similar quantities of these library samples for further PCR amplification reactions.

### Small RNA sequencing and sequence processing

The mixed small RNA samples were enriched by poly-ethylene glycol precipitation, separated on 15% denaturing PAGE, and visualized by SYBR-gold staining. Small RNAs of 15-30 nt were gel-purified, and then were ligated to a 5'-adaptor and a 3'- adaptor sequentially, which were reversely transcribed to cDNA with the RT primer (CAAGCAGAAGACGGCATACGA) using Superscript II reverse transcriptase (Invitrogen) [23] for Solexa sequencing and sequence processing by Beijing Genomics Institute (BGI) (Shenzhen, Guangdong, China).

### Bioinformatics analysis and identification of va-miRNA and miRNA SNPs

To identify conserved and potential va-miRNAs in grapevine, the raw sequences were processed as described by Sunkar *et al. *[55]. The vector sequences of all sRNAs sequences from 18nt to 30nt were removed, and then the modified sequences were mapped to the grapevine genome, whereby sequences that matched the genome were further subjected to removal of rRNA, tRNA, snRNA, snoRNA and all those containing the polyA tails. The remaining sequences were then compared against known plant miRNAs existing in the miRBase 16.0. Only matching (0-3mismatches) sequences were considered as conserved va-miRNAs, while other sequences that are similar to conserved va-miRNAs, but have one base variation with the conserved va-miRNAs can both be considered one miRNA SNP.

To study potential va-miRNAs precursor sequences, all sRNAs from grapevine were aligned against the grapevine genome and then the miRNA candidates were processed by miRCat http://srna-tools.cmp.uea.ac.uk/[60], using default parameters, to generate the secondary structures.

### Analyses of miRNA by 5'miR-RACE and 3'miR-RACE

The cDNA was amplified with the mirRacer 5' primer (5'- GGACACTGACATGGACTGAAGGAGTA-3') and the mirRacer 3' primer (5'-ATTCTAGAGGCCGAGGCGGCCGACATG-3') to generate a pool of non-gene-specific product. 5' miR-RACE reactions were performed with the mirRacer 5' primer and miRNA-gene-specific forward primers, and 3' miR-RACE reactions were carried out with the mirRacer 3' primer and miRNA-gene-specific reverse primers (Additional file 8 and Additional file 9), as described by Song *et al. *[36], with minor modifications. The 5' RACE and 3' RACE clones with PCR products of about 56 bp and 87 bp were sequenced (Invitrogen), respectively.

### Prediction of potential target mRNAs for va-miRNAs

Target predictions were performed based on methods described by Allen *et al. *[44]. Putative va-miRNAs were first blasted against the grapevine unigene database on the Genoscope http://www.genoscope.cns.fr/. BLASTn hits possessing less than four mismatches were chosen as the candidate targets, and then BLASTx was used to obtain their putative functions.

### Data access

The sRNA sequence data from this study have been submitted to Gene Expression Omnibus (GEO) under accession No. GSE34169 at website: http://www.ncbi.nlm.nih.gov/geo/query/acc.cgi?acc=GSE34169.

## Competing interests

The authors declare that they have no competing interests.

## Authors' contributions

WC carried out the laboratory work and wrote this paper. WC and SG performed bioinformatics analyses. HJ and LC participated in coordination of the study. EK and LX constructed the sRNA library. KKN revised this paper. FJ conceived, designed the study and revised this paper. All authors read and approved the final manuscript.

## Supplementary Material

Additional file 1 **Size distribution of unique small RNA sequences from Amur grape**. Click here for file

Additional file 2 **Identified conserved miRNAs from Amur grape**. Click here for file

Additional file 3 **Predicted secondary structure of new potential miRNAs from Amur grape**. Click here for file

Additional file 4 **3'RACE and 5' RACE products of Vv-miRNAs amplified by PCR shown in an ethidium bromide-stained agarose gel**. Sizes of the molecular weight markers of the bottom and the second bottom bands are 50 bp and 100 bp on A, B, C and D, respectively. Lanes 1-20 are 3'RACE (A) and 5'RACE (B) products of 20 lower abundance va-miRNAs (va-miR398b, va-miR399c, va-miR828a, va-miR005, va-miR011, va-miR020, va-miR028, va-miR029, va-miR032, va-miR037, va-miR045, va-miR048, va-miR052, va-miR063, va-miR066, va-miR074, va-miR077, va-miR082, va-miR095 and va-miR098, respectively). Lanes 1'-20' are 3'RACE (C) and 5'RACE (D) products of 20 higher abundance va-miRNAs (va-miR156e, va-miR160c, va-miR162, va-miR164c, va-miR166c, va-miR169m, va-miR171c, va-miR172c, va-miR408, va-miR535a, va-miR001, va-miR007, va-miR016, va-miR018, va-miR023, va-miR046, va-miR047, va-miR049, va-miR057 and va-miR062, respectively). The sizes of 3'RACE products are about 83 bp while the size of 5'RACE products are about 57 bp. Click here for file

Additional file 5 **List of SNPs of conserved miRNAs in Amur grape**. Click here for file

Additional file 6 **List of miR-LDs of conserved miRNAs in Amur grape**. Click here for file

Additional file 7 **List of predicted target genes of non-conserved miRNAs identified in Amur grape**. Click here for file

Additional file 8 **List of primers of miRNAs used for miR-5'RACE and miR-3'RACE**. Click here for file

Additional file 9 **List of primers of va-miR-LDs used for miR-RACE**. Click here for file

## References

[B1] Phillips J Dalmay T Bartels D The role of small RNAs in abiotic stress FEBS Letters 2007 581 3592 3597 10.1016/j.febslet.2007.04.007 17451688

[B2] Li Y Zheng Y Addo-Quaye C Zhang L Saini A Jagadeeswaran G Axtell MJ Zhang W Sunkar R Transcriptome-wide identification of microRNA targets in rice Plant J 2010 62 742 759 10.1111/j.1365-313X.2010.04187.x 20202174

[B3] Brodersen P Sakvarelidze-Achard L Bruun-Rasmussen M Dunoyer P Yamamoto YY Sieburth L Voinnet O Widespread translational inhibition by plant miRNAs and siRNAs Science 2008 320 1185 1190 10.1126/science.1159151 18483398

[B4] Jones-Rhoades MW Bartel DP Bartel B MicroRNAs and their regulatory roles in plants Annual Review of Plant Biology 2006 57 19 53 10.1146/annurev.arplant.57.032905.105218 16669754

[B5] Lauter N Kampani A Carlson S Goebel M Moose SP microRNA172 downregulates glossy15 to promote vegetative phase change in maize Proc Natl Acad Sci USA 2005 102 9412 9417 10.1073/pnas.0503927102 15958531 PMC1166634

[B6] Schwarz S Grande AV Bujdoso N Saedler H Huijser P The microRNA regulated SBP-box genes *SPL9 *and *SPL15 *control shoot maturation in *Arabidopsis* Plant Mol Biol 2008 67 183 1895 10.1007/s11103-008-9310-z 18278578 PMC2295252

[B7] Jung JH Park CM *MIR166/165 *genes exhibit dynamic expression patterns in regulating shoot apical meristem and floral development in *Arabidopsis* Planta 2007 225 1327 1338 10.1007/s00425-006-0439-1 17109148

[B8] Ochando I Jover-Gil S Ripoll JJ Candela H Vera A Ponce MR Martínez-Laborda A Micol JL Mutations in the microRNA complementarity site of the *INCURVATA4 *gene perturb meristem function and adaxialize lateral organs in *Arabidopsis* Plant Physiol 2006 141 607 619 10.1104/pp.106.077149 16617092 PMC1475466

[B9] Borsani O Zhu J Verslues PE Sunkar R Zhu JK Endogenous siRNAs derived from a pair of natural cis-antisense transcripts regulate salt tolerance in *Arabidopsis* Cell 2005 123 1279 1291 10.1016/j.cell.2005.11.035 16377568 PMC3137516

[B10] Lu S Sun YH Shi R Clark C Li L Chiang VL New and mechanical stress-responsive microRNAs in Populus trichocarpa that are absent from *Arabidopsis* Plant Cell 2005 17 2186 2203 10.1105/tpc.105.033456 15994906 PMC1182482

[B11] Zhang BH Pan X Cannon CH Cobb GP Anderson TA Conservation and divergence of plant microRNA genes Plant J 2006 46 243 259 10.1111/j.1365-313X.2006.02697.x 16623887

[B12] Zhao CZ Xia H Frazier TP Yao YY Bi YP Li YP Li AQ Li MJ Zhang BH Wang XJ Deep sequencing identifies new and conserved microRNAs in peanuts (*Arachis hypogaea *L.) BMC Plant Biol 2010 10 3 10.1186/1471-2229-10-3 20047695 PMC2826338

[B13] Felippes FF Schneeberger K Dezulain T Huson DH Weigel D Evolution of *Arabidopsis thaliana *microRNAs from random sequences RNA 2008 14 2455 2459 10.1261/rna.1149408 18952822 PMC2590950

[B14] Sunkar R Zhou X Zheng Y Zhang W Zhu JK Identification of new and candidate Vv-miRNAs in rice by high throughput sequencing BMC Plant Biol 2008 8 25 10.1186/1471-2229-8-25 18312648 PMC2292181

[B15] Szittya G Moxon S Santos DM Jing R Fevereiro MP Moulton V Dalmay T High-throughput sequencing of *Medicago truncatula *short RNAs identifies eight new Vv-miRNA families BMC Genomics 2008 9 593 10.1186/1471-2164-9-593 19068109 PMC2621214

[B16] Zhang BH Pan XP Stellwag EJ Identification of soybean microRNAs and their targets Planta 2008 229 161 182 10.1007/s00425-008-0818-x 18815805

[B17] Song CN Fang JG Li XY Liu H Chao TC Identification and characterization of 27 conserved microRNAs in citrus Planta 2009 230 671 685 10.1007/s00425-009-0971-x 19585144 PMC2729984

[B18] Song CN Wang C Zhang CQ Nicholas KK Yu HP Ma ZQ Fang JG Deep sequencing discovery of new and conserved microRNAs in trifoliate orange (Citrus rifoliate) BMC Genomics 2010 11 431 10.1186/1471-2164-11-431 20626894 PMC2996959

[B19] Yu HP Song CN Jia QD Wang C Li F Nicholas KK Zhang XY Fang JG Computational identification of microRNAs in apple expressed sequence tags and validation of their precise sequences by miR-RACE Physiologia Plantarum 2011 141 56 70 10.1111/j.1399-3054.2010.01411.x 20875055

[B20] Pantaleo V Szittya G Moxon S Miozzi L Moulton V Dalmay T Burgyan J Identification of grapevine microRNAs and their targets using high-throughput sequencing and degradome analysis The Plant Journal 2010 62 960 976 20230504 10.1111/j.0960-7412.2010.04208.x

[B21] Mica E Piccolo V Delledonne M Ferrarini A Pezzotti M Casati C Fabbro CD Valle G Policriti A Morgante M Pesole G Horner MpèDS Correction: high throughput approaches reveal splicing of primary microRNA transcripts and tissue specific expression of mature microRNAs in Vitis vinifera BMC Genomics 2010 11 109 10.1186/1471-2164-11-109 20152027 PMC2831844

[B22] Carra A Mica E Gambino G Pindo M Moser C Pè ME Schubert A Cloning and characterization of small non-coding RNAs from grapevine Plant J 2009 59 750 763 10.1111/j.1365-313X.2009.03906.x 19453456

[B23] Wang C Wang X Nicholas KK Song C Zhang C Li X Han J Fang J Deep sequencing of grapevine flower and berry short RNA library for discovery of new microRNAs and validation of precise sequences of grapevine microRNAs deposited in miRBase Physiologia Plantarum 2011 143 64 81 10.1111/j.1399-3054.2011.01481.x 21496033

[B24] Wang C Shangguan LF Nicholas KK Wang XC Han J Song CN Fang JG Characterization of microRNAs identified in a table grapevine cultivar with validation of computationally predicted grapevine miRNAs by miR-RACE PLoSone 2011 6 e21259 10.1371/journal.pone.0021259 PMC3145640 21829435

[B25] Liang CW Zhang XW Zou J Xu D Su F Ye NH Identification of miRNA from *Porphyra yezoensis *by High-Throughput Sequencing and Bioinformatics Analysis PLoSone 2010 5 e10698 10.1371/journal.pone.0010698 PMC2873431 20502668

[B26] Rajagopalan R Vaucheret H Trejo J Bartel DP A diverse and evolutionarily fluid set of microRNAs in *Arabidopsis thaliana* Genes Development 2006 20 3407 3425 10.1101/gad.1476406 17182867 PMC1698448

[B27] Dezulian T Palatnik J Huson D Weigel D Conservation and divergence of microRNA families in plants Genome Biology 2005 6 13

[B28] Fahlgren N Howell MD Kasschau KD Chapman EJ Sullivan CM Cumbie JS Givan SA Law TF Grant SR Dangl JL Carringtonet JC High-throughput sequencing of *Arabidopsis *micro-RNAs: evidence for frequent birth and death of MiRNA genes PLoSone 2007 2 e219 10.1371/journal.pone.0000219 PMC1790633 17299599

[B29] Barakat A Wall PK Diloreto S Depamphilis CW Carlson JE Conservation and divergence of microRNAs in populus BMC Genomic 2007 8 481 10.1186/1471-2164-8-481 PMC2270843 18166134

[B30] Fahlgren N Jogdeo S Kasschau KD Sullivan CM Chapman EJ Laubinger S Smith LM Dasenko M Givan SA Weigel D Carrington JC MicroRNA Gene Evolution in *Arabidopsis lyrata *and *Arabidopsis thaliana* The Plant Cell 2010 22 1074 1089 10.1105/tpc.110.073999 20407027 PMC2879733

[B31] Dohm JC Lottaz C Borodina T Himmelbauer H Substantial biases in ultra-short read data sets from high-throughput DNA sequencing Nucleic Acids Research 2008 36 e105 10.1093/nar/gkn425 18660515 PMC2532726

[B32] Meyers BC Axtell MJ Bartel B Bartel DP Baulcombe D Bowman JL Cao XF Carrington JC Chen XM Green PJ Griffiths-Jones S Jacobsen SE Mallory AC Martienssen RA Poethig RS Qi YJ Vaucheret H Voinnet O Watanabe Y Weigel D Zhu JK Criteria for annotation of plant MicroRNAs Plant Cell 2008 20 3186 3190 10.1105/tpc.108.064311 19074682 PMC2630443

[B33] Yao YY Guo GG Ni ZF Sunkar R Zu JK Sun QX Cloning and characterization of microRNAs from wheat(Triticumaestivum L.) Genome Biology 2007 8 R96 10.1186/gb-2007-8-6-r96 17543110 PMC2394755

[B34] Zhang BH Pan XP Anderson TA Identification of 188 conserved maize microRNAs and their targets FEBS Lett 2006 580 3753 3762 10.1016/j.febslet.2006.05.063 16780841

[B35] Lu C Kulkarni K Souret FF Muthuvalliappan R Tej SS Poethig RS Henderson IR Jacobsen SE Wang WZ Green PJ Meyers BC MicroRNAs and other small RNAs enriched in the *Arabidopsis *RNA-dependent RNA polymerase-2 mutant Genome Res 2006 16 1276 1288 10.1101/gr.5530106 16954541 PMC1581437

[B36] Song CN Fang JG Wang C Guo L Nicholas KK Ma ZQ MiR-RACE, a new efficient approach to determine the precise sequences of computationally identified trifoliate orange (Poncirus rifoliate) MicroRNAs PLoSone 2010 5 e10861 10.1371/journal.pone.0010861 PMC2881865 20539756

[B37] Sun GH Yan J Noltner K Feng J Li HT Sardis DA Sommer SS Rossi JJ SNPs in human miRNA genes affect biogenesis and function RNA 2009 15 1640 1651 10.1261/rna.1560209 19617315 PMC2743066

[B38] Liu D Fan J Mei MX Ingvarsson S Chen HP Identification of miRNAs in a liver of a human fetus by a modified method PLoSone 2009 4 e7594 10.1371/journal.pone.0007594 PMC2762743 19855840

[B39] Ehrenreich IM Purugganan MD Sequence variation of MicroRNAs and their binding sites in *Arabidopsis* Plant Physiology 2008 146 1974 1982 10.1104/pp.108.116582 18305205 PMC2287364

[B40] Lai EC Tomancak P Williams RW Rubin GM Computational identification of Drosophila microRNA genes Genome Biol 2003 4 R42 10.1186/gb-2003-4-7-r42 12844358 PMC193629

[B41] Bonnet E Wuyts J Rouze P Peer VY Detection of 91 potential in plant conserved plant microRNAs in *Arabidopsis thaliana *and *Oryza sativa *identifies important target genes Proc Natl Acad Sci USA 2004 101 11511 11516 10.1073/pnas.0404025101 15272084 PMC509231

[B42] Rhoades MW Reinhart BJ Lim LP Burge CB Bartel B Bartel DP Prediction of plant microRNA targets Cell 2002 110 513 520 10.1016/S0092-8674(02)00863-2 12202040

[B43] Song CN Jia QD Fang JG Li F Wang C Zhang Z Computational identification of citrus microRNAs and target analysis in citrus expressed sequence tags Plant Biology 2009 12 927 934 21040308 10.1111/j.1438-8677.2009.00300.x

[B44] Allen E Xie Z Gustafson AM Carrington JC MicroRNA-directed phasing during trans-acting siRNA biogenesis in plants Cell 2005 12 207 221 15851028 10.1016/j.cell.2005.04.004

[B45] Wu J Zhang Yl Zhang HQ Huang H Folta KM Lu J Whole genome wide expression profiles of Vitis amurensis grape responding to downy mildew by using Solexa sequencing technology BMC Plant Biology 2010 10 234 10.1186/1471-2229-10-234 21029438 PMC3017854

[B46] Zhan BH Wang QL Pan XP MicroRNAs and their regulatory roles in animals and plants Journal of Cellular Physiology 2007 210 279 289 10.1002/jcp.20869 17096367

[B47] Zhang J Zeng R Chen J Liu X Liao Q Identification of conserved microRNAs and their targets from Solanum lycopersicum Mill Gene 2008 423 1 7 10.1016/j.gene.2008.05.023 18602455

[B48] Floyd SK Bowman JL Gene regulation: ancient micro-RNA target sequences in plants Nature 2004 428 485 486 10.1038/428485a 15057819

[B49] Floyd SK Zalewski CS Bowman JL Evolution of class III homeodomain leucine zipper genes in streptophytes Genetics 2006 173 373 388 10.1534/genetics.105.054239 16489224 PMC1461458

[B50] Arazi T Talmor-Neiman M Stav R Riese M Huijser P Baulcombe DC Cloning and characterization of micro-RNAs from moss Plant Journal 2005 43 837 848 10.1111/j.1365-313X.2005.02499.x 16146523

[B51] Axtell MJ Bartel DP Antiquity of microRNAs and their targets in land plants Plant Cell 2005 17 1658 1673 10.1105/tpc.105.032185 15849273 PMC1143068

[B52] Fattash I Voss B Reski R Hess WR Frank W Evidence for the rapid expansion of microRNA-mediated regulation in early land plant evolution BMC Plant Biology 2007 7 13 10.1186/1471-2229-7-13 17359535 PMC1838911

[B53] Jaillon O Aury JM Noel B Policriti A Clepet C Casagrande A Choisne N Aubourg S Vitulo N Jubin C Vezzi A Legeai F Hugueney P Dasilva C Horner D Mica E Jublot D Poulain J Bruyère C Billault A Segurens B Gouyvenoux M Ugarte E Cattonaro F Anthouard V Vico V Fabbro CD Alaux M Gaspero GD Dumas V Felice N Paillard S Juman I Moroldo M Scalabrin S Canaguier A Clainche IL Malacrida G Durand E Pesole G Laucou V Chatelet P Merdinoglu D Delledonne M Pezzotti M Lecharny A Scarpelli C Artiguenave PèG Valle F ME Morgante M Caboche M Adam-Blondon AF Weissenbach J Quétier F Wincker P French-Italian Public Consortium for Grapevine Genome Characterization: The grapevine genome sequence suggests ancestral hexaploidization in major angiosperm phyla Nature 2007 449 463 467 10.1038/nature06148 17721507

[B54] Jones-Rhoades MW Bartel DP Computational identification of plant microRNAs and their targets, including a stress induced miRNA Molecular Cell 2004 14 787 799 10.1016/j.molcel.2004.05.027 15200956

[B55] Sunkar R Girke T Jain PK Zhu JK Cloning and characterization of microRNAs from rice Plant Cell 2005 17 1397 1411 10.1105/tpc.105.031682 15805478 PMC1091763

[B56] Sunkar R Girke T Zhu JK Identification and characterization of endogenous small interfering RNAs from Rice Nucleic Acids Research 2005 33 4443 4454 10.1093/nar/gki758 16077027 PMC1182700

[B57] Moxon S Jing RC Szittya G Schwach F Rusholme Pilcher RL Moulton V Dalmay T Deep sequencing of tomato short RNAs identifies microRNAs targeting genes involved in fruit ripening Genome Research 2008 18 1602 1609 10.1101/gr.080127.108 18653800 PMC2556272

[B58] Grun D Wang YL Langenberger D Gunsalus KC Rajewsky N microRNA target predictions across seven Drosophila species and comparison to mammalian targets PloS Comput Biol 2005 1 e13 10.1371/journal.pcbi.0010013 16103902 PMC1183519

[B59] Chang SJ Jepf P Johnc A Simple and efficient method for isolating RNA from pine trees Plant Mol Biol Reporter 1993 11 113 116 10.1007/BF02670468

[B60] Moxon S Schwach F Maclean D Dalmay T Studholme DJ Moulton V A tool kit for analyzing large-scale plant small RNA datasets Bioinformatics 2008 24 2252 2253 10.1093/bioinformatics/btn428 18713789

