# Supplementary material for: Identification of microRNAs from Amur grape (vitis amurensis Rupr.) by deep sequencing and analysis of microRNA variations with bioinformatics
Source: BMC Genomics. 2012 Mar 29;13:122. doi: 10.1186/1471-2164-13-122 (PMC3353164; doi:10.1186/1471-2164-13-122)
Supplement: Additional file 2 — Identified conserved miRNAs from Amur grape. [file 1471-2164-13-122-S2.DOC]

**Supplementary materials for online publication only**

| Table S1 | | |  |  |  |
| --- | --- | --- | --- | --- | --- |
| MiRNA family | miRNA ID | sequences | Length | unique reads | redundant reads |
| miR156 | va-miR156a | TTGACAGAAGAGAGGGAGCAC | 21 | 2447 | 2549 |
|  | va-miR156b | TGACAGAAGAGAGTGAGCAC | 20 | 33346 | 40071 |
|  | va-miR156c | TGACAGAAGAGAGTGAGCAC | 20 | 33346 | 38182 |
|  | va-miR156d | TGACAGAAGAGAGTGAGCAC | 20 | 33346 | 40759 |
|  | va-miR156e | TGACAGAGGAGAGTGAGCAC | 20 | 731 | 737 |
|  | va-miR156f | TTGACAGAAGATAGAGAGCAC | 21 | 24514 | 25012 |
|  | va-miR156g | TTGACAGAAGATAGAGAGCAC | 21 | 24514 | 24947 |
|  | va-miR156i | TTGACAGAAGATAGAGAGCAC | 21 | 24514 | 24947 |
| miR159 | va-miR159a | CCTTGGAGTGAAGGGAGCT | 19 | 5 | 9 |
|  | va-miR159b | CCTTGGAGTGAAGGGAGCT | 19 | 5 | 9 |
|  | va-miR159c | TTTGGATTGAAGGGAGCTCTA | 21 | 2459 | 3458 |
| miR160 | va-miR160a | TGCCTGGCTCCCTGAATGCCA | 21 | 26 | 29 |
|  | va-miR160b | TGCCTGGCTCCCTGAATGCCA | 21 | 26 | 29 |
|  | va-miR160c | TGCCTGGCTCCCTGTATGCCA | 21 | 280 | 283 |
|  | va-miR160d | TGCCTGGCTCCCTGTATGCCA | 21 | 280 | 290 |
|  | va-miR160e | TGCCTGGCTCCCTGAATGCCA | 21 | 26 | 29 |
|  | va-miR160f | TGCCTGGCTCCCTGTATGCCA | 21 | 280 | 290 |
| miR162 | va-miR162 | TCGATAAACCTCTGCATCCAG | 21 | 3494 | 3539 |
| miR164 | va-miR164a | TGGAGAAGCAGGGCACGTGCA | 21 | 6861 | 6907 |
|  | va-miR164c | TGGAGAAGCAGGGCACGTGCA | 21 | 6861 | 6947 |
|  | va-miR164d | TGGAGAAGCAGGGCACGTGCA | 21 | 6861 | 6907 |
| miR166 | va-miR166a | TCTCGGACCAGGCTTCATTCC | 21 | 21867 | 39371 |
|  | va-miR166b | TCGGACCAGGCTTCATTCCTC | 21 | 194294 | 212224 |
|  | va-miR166c | TCGGACCAGGCTTCATTCCCC | 21 | 326941 | 349795 |
|  | va-miR166d | TCGGACCAGGCTTCATTCCCC | 21 | 326941 | 350478 |
|  | va-miR166e | TCGGACCAGGCTTCATTCCCC | 21 | 326941 | 349795 |
|  | va-miR166f | TCGGACCAGGCTTCATTCCCC | 21 | 326941 | 355814 |
|  | va-miR166g | TCGGACCAGGCTTCATTCCCC | 21 | 326941 | 357020 |
|  | va-miR166h | TCGGACCAGGCTTCATTCCCC | 21 | 326941 | 372442 |
| miR167 | va-miR167a | TGAAGCTGCCAGCATGATCT | 20 | 777 | 2281 |
|  | va-miR167b | TGAAGCTGCCAGCATGATCTAA | 22 | 68590 | 105974 |
|  | va-miR167c | TGAAGCTGCCAGCATGATCTC | 21 | 63479 | 78511 |
|  | va-miR167d | TGAAGCTGCCAGCATGATCTA | 21 | 33860 | 35574 |
|  | va-miR167e | TGAAGCTGCCAGCATGATCTAA | 22 | 68590 | 105323 |
| miR168 | va-miR168 | TCGCTTGGTGCAGGTCGGGAA | 21 | 63449 | 65885 |
| miR169 | va-miR169a | CAGCCAAGGATGACTTGCCGG | 21 | 433 | 452 |
|  | va-miR169b | TGAGCCAAGGATGGCTTGCCGT | 22 | 259 | 271 |
|  | va-miR169c | CAGCCAAGGATGACTTGCCGG | 21 | 433 | 458 |
|  | va-miR169d | CAGCCAAGAATGATTTGCCGG | 21 | 7398 | 8184 |
|  | va-miR169e | TAGCCAAGGATGACTTGCCT | 20 | 23 | 47 |
|  | va-miR169f | CAGCCAAGGATGACTTGCCGA | 21 | 80 | 88 |
|  | va-miR169g | CAGCCAAGGATGACTTGCCGA | 21 | 80 | 93 |
|  | va-miR169h | TGAGCCAAGGATGGCTTGCCGT | 22 | 259 | 265 |
|  | va-miR169i | TGAGCCAAGGATGACTGGCCGT | 22 | 11 | 11 |
|  | va-miR169j | CAGCCAAGGATGACTTGCCGG | 21 | 433 | 452 |
|  | va-miR169k | CAGCCAAGGATGACTTGCCGG | 21 | 433 | 452 |
|  | va-miR169l | TGAGCCAAGGATGACTTGCCG | 21 | 666 | 752 |
|  | va-miR169m | TGAGCCAAGGATGACTTGCCG | 21 | 666 | 683 |
|  | va-miR169n | TAGAGCCAAGGATGACTTGCCG | 22 | 311 | 362 |
|  | va-miR169o | TGAGCCAAGGATGACTTGCCG | 21 | 666 | 693 |
|  | va-miR169p | TGAGCCAAGGATGACTTGCCG | 21 | 666 | 700 |
|  | va-miR169q | TAGAGCCAAGGATGACTTGCCG | 22 | 311 | 364 |
|  | va-miR169r | TGAGTCAAGGATGACTTGCCGA | 22 | 437 | 475 |
|  | va-miR169s | CAGCCAAGGATGACTTGCCGG | 21 | 433 | 448 |
|  | va-miR169t | CGAGTCAAGGATGACTTGCCGA | 22 | 13 | 17 |
|  | va-miR169u | TGAGTCAAGGATGACTTGCCG | 21 | 19 | 26 |
|  | va-miR169v | AAGCCAAGGATGAATTGCCGG | 21 | 493 | 508 |
|  | va-miR169w | CAGCCAAGGATGACTTGCCGG | 21 | 433 | 448 |
|  | va-miR169x | TAGCCAAGGATGACTTGCCT | 20 | 23 | 32 |
| miR171 | va-miR171a | TGATTGAGCCGTGCCAATATC | 21 | 2337 | 2438 |
|  | va-miR171b | TTGAGCCGCGTCAATATCTCC | 21 | 410 | 439 |
|  | va-miR171c | TGATTGAGCCGTGCCAATATC | 21 | 2337 | 2548 |
|  | va-miR171d | TGATTGAGCCGTGCCAATATC | 21 | 2337 | 2548 |
|  | va-miR171e | TTGAGCCGCGCCAATATCACT | 21 | 56 | 69 |
|  | va-miR171f | TTGAGCCGCGCCAATATCACT | 21 | 56 | 71 |
|  | va-miR171h | TTGAGCCGCGCCAATATCCCG | 21 | 11 | 11 |
|  | va-miR171i | TGATTGAGCCGTGCCAATATC | 21 | 2337 | 2438 |
| miR172 | va-miR172c | GGAATCTTGATGATGCTGCAG | 21 | 499 | 561 |
|  | va-miR172d | TGAGAATCTTGATGATGCTGC | 21 | 2337 | 4018 |
| miR319 | va-miR319b | TTGGACTGAAGGGAGCTCCCT | 21 | 48 | 78 |
|  | va-miR319c | TGCTTGGACTGAAGGGAG | 18 | 79 | 178 |
|  | va-miR319e | TTTGGACTGAAGGGAGCTCCT | 21 | 37 | 46 |
|  | va-miR319f | TGCTTGGACTGAAGGGAG | 18 | 79 | 178 |
|  | va-miR319g | TTGGACTGAAGGGAGCTCCC | 20 | 14 | 15 |
| miR390 | va-miR390 | AAGCTCAGGAGGGATAGCGCC | 21 | 2644 | 3834 |
| miR393 | va-miR393a | TCCAAAGGGATCGCATTGAT | 20 | 23 | 63 |
|  | va-miR393b | TCCAAAGGGATCGCATTGAT | 20 | 23 | 62 |
| miR394 | va-miR394a | TTGGCATTCTGTCCACCTCC | 20 | 39 | 48 |
|  | va-miR394b | TTGGCATTCTGTCCACCTCC | 20 | 39 | 42 |
|  | va-miR394c | TTGGCATTCTGTCCACCTCC | 20 | 39 | 48 |
| miR395 | va-miR395a | CTGAAGTGTTTGGGGGAACTC | 21 | 26 | 54 |
|  | va-miR395b | CTGAAGTGTTTGGGGGAACTC | 21 | 26 | 54 |
|  | va-miR395c | CTGAAGTGTTTGGGGGAACTC | 21 | 26 | 54 |
|  | va-miR395d | CTGAAGTGTTTGGGGGAACTC | 21 | 26 | 54 |
|  | va-miR395e | CTGAAGTGTTTGGGGGAACTC | 21 | 26 | 54 |
|  | va-miR395f | CTGAAGTGTTTGGGGGAACTC | 21 | 26 | 54 |
|  | va-miR395g | CTGAAGTGTTTGGGGGAACTC | 21 | 26 | 54 |
|  | va-miR395h | CTGAAGTGTTTGGGGGAACTC | 21 | 26 | 54 |
|  | va-miR395i | CTGAAGTGTTTGGGGGAACTC | 21 | 26 | 54 |
|  | va-miR395j | CTGAAGTGTTTGGGGGAACTC | 21 | 26 | 54 |
|  | va-miR395k | CTGAAGTGTTTGGGGGAACTC | 21 | 26 | 54 |
|  | va-miR395l | CTGAAGTGTTTGGGGGAACTC | 21 | 26 | 54 |
|  | va-miR395m | CTGAAGTGTTTGGGGGAACTC | 21 | 26 | 54 |
| miR396 | va-miR396a | TTCCACAGCTTTCTTGAA | 18 | 2823 | 3866 |
|  | va-miR396b | TTCCACAGCTTTCTTGAA | 18 | 2823 | 4061 |
|  | va-miR396c | TTCCACAGCTTTCTTGAA | 18 | 2823 | 4479 |
|  | va-miR396d | TTCCACAGCTTTCTTGAA | 18 | 2823 | 4658 |
| miR397 | va-miR397a | TCATTGAGTGCAGCGTTGATG | 21 | 279 | 345 |
|  | va-miR397b | TCATTGAGTGCAGCGTTGATG | 21 | 279 | 345 |
|  | va-miR398a | TTCTCAGGTCACCCCTTTGGG | 21 | 13 | 14 |
| miR398 | va-miR398b | TGTGTTCTCAGGTCGCCCCTG | 21 | 5 | 8 |
|  | va-miR398c | TGTGTTCTCAGGTCGCCCCTG | 21 | 11 | 11 |
| miR399 | va-miR399a | CAAAGGAGAATTGCCCTGTTA | 21 | 14 | 53 |
|  | va-miR399b | TGCCAAAGGAGAGTTGCCCTG | 21 | 6 | 15 |
|  | va-miR399c | TGCCAAAGGAGAGTTGCCCTG | 21 | 7 | 9 |
|  | va-miR399d | AAAGGAGATTTGCTCGTGAAT | 21 | 5 | 15 |
|  | va-miR399e | TGCCAAAGGAGATTTGCCCGG | 21 | 91 | 103 |
|  | va-miR399g | TGCCAAAGGAGATTTGCCCCT | 21 | 18 | 33 |
|  | va-miR399h | TGCCAAAGGAGAATTGCC | 18 | 13 | 36 |
|  | va-miR399i | CGCCAAAGGAGAGTTGCCCTG | 21 | 154 | 213 |
| miR403 | va-miR403a | TTAGATTCACGCACAAACT | 19 | 3119 | 4676 |
|  | va-miR403b | TTAGATTCACGCACAAACT | 19 | 3119 | 4680 |
|  | va-miR403c | TTAGATTCACGCACAAACT | 19 | 3119 | 4676 |
|  | va-miR403d | TTAGATTCACGCACAAACT | 19 | 3119 | 4680 |
|  | va-miR403e | TTAGATTCACGCACAAACT | 19 | 3119 | 4675 |
|  | va-miR403f | TTAGATTCACGCACAAACT | 19 | 3119 | 4724 |
| miR408 | va-miR408 | ATGCACTGCCTCTTCCCTGGC | 21 | 189 | 216 |
| miR477 | va-miR477 | TCCCTCAAAGGCTTCCAATTT | 21 | 222 | 342 |
| miR479 | va-miR479 | TGTGGTATTGGTTCGGCTCATC | 22 | 20935 | 22613 |
| miR482 | va-miR482 | TCTTTCCTACTCCTCCCATTCC | 22 | 1629 | 1673 |
| miR535 | va-miR535a | TGACAACGAGAGAGAGCACGC | 21 | 6649 | 6980 |
|  | va-miR535b | TGACAACGAGAGAGAGCACGC | 21 | 6649 | 6980 |
|  | va-miR535c | TGACAACGAGAGAGAGCACGC | 21 | 6649 | 6980 |
|  | va-miR535d | TGACAACGAGAGAGAGCACGC | 21 | 6649 | 6980 |
|  | va-miR535e | TGACAACGAGAGAGAGCACGC | 21 | 6649 | 6980 |
| miR828 | va-miR828a | TCTTGCTCAAATGAGTATTCCA | 22 | 6 | 9 |
