# Supplementary material for: Identification of microRNAs from Amur grape (vitis amurensis Rupr.) by deep sequencing and analysis of microRNA variations with bioinformatics
Source: BMC Genomics. 2012 Mar 29;13:122. doi: 10.1186/1471-2164-13-122 (PMC3353164; doi:10.1186/1471-2164-13-122)
Supplement: Additional file 3 — Predicted secondary structure of new potential miRNAs from Amur grape. [file 1471-2164-13-122-S3.DOC]

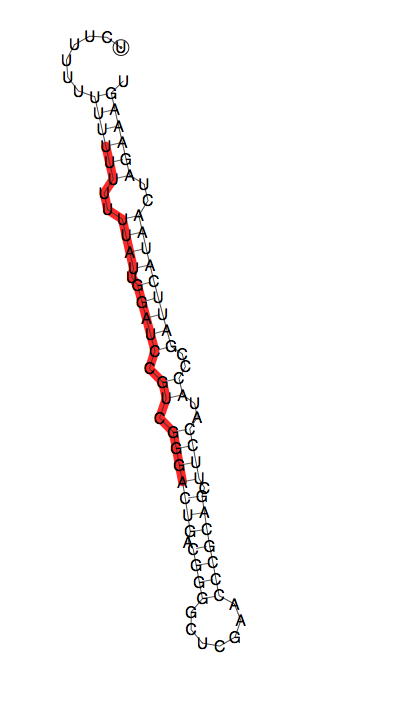


**Secondary structure for ‘va-miR001’**


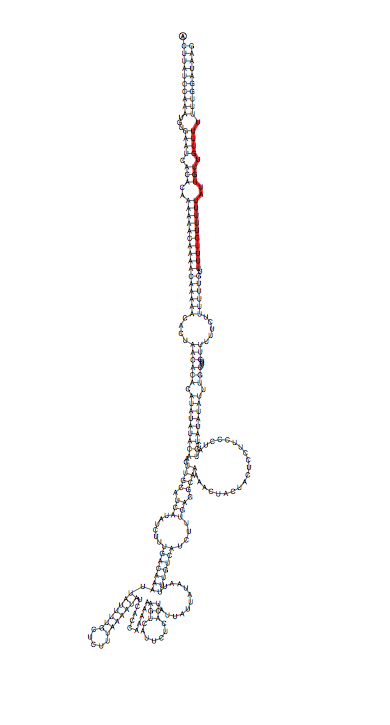


**Secondary structure for ‘va-miR002’**


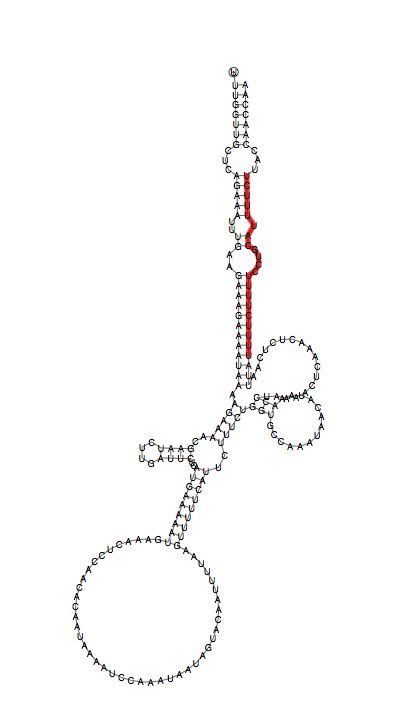


**Secondary structure for ‘va-miR003’**


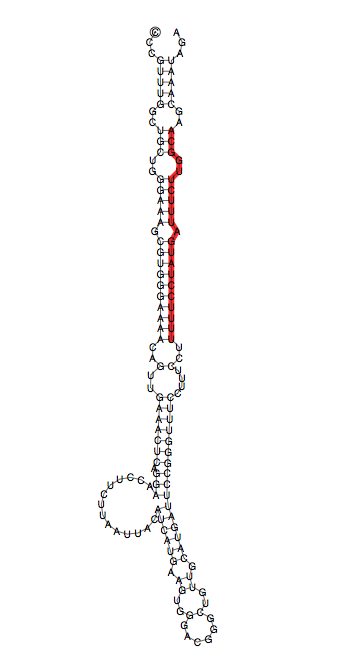


**Secondary structure for ‘va-miR004’**


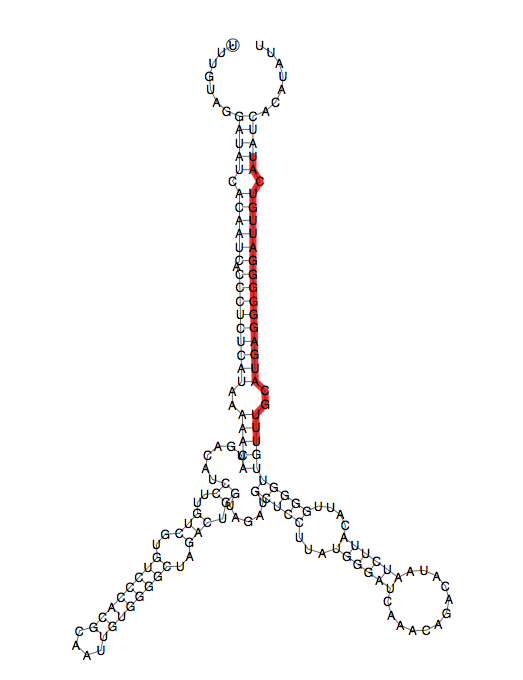


**Secondary structure for ‘va-miR005’**


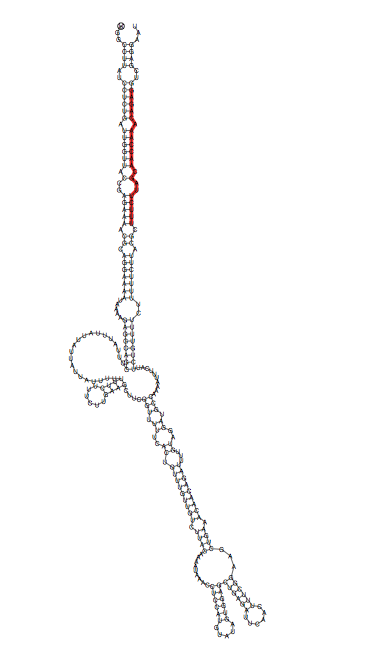


**Secondary structure for ‘va-miR006a’**


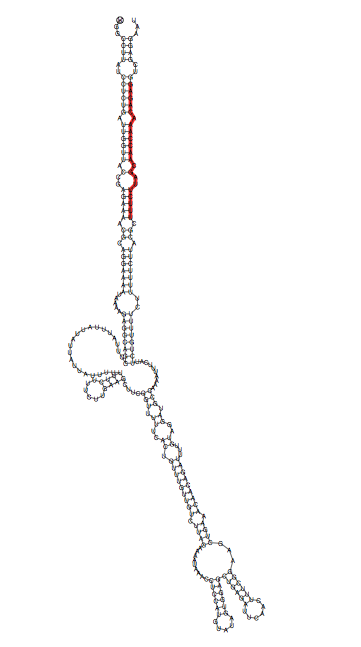


**Secondary structure for ‘va-miR006b’**


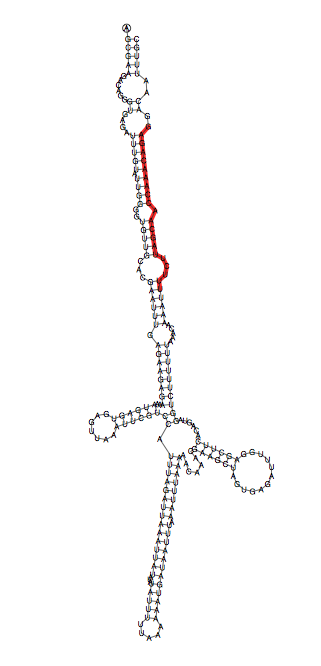


**Secondary structure for ‘va-miR006c’**


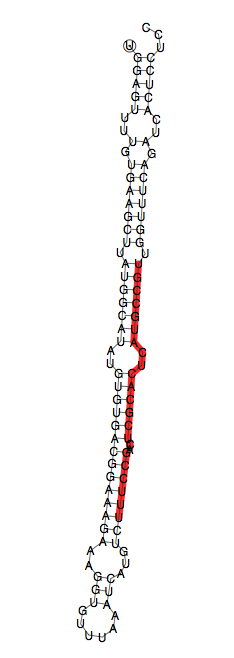


**Secondary structure for ‘va-miR007’**


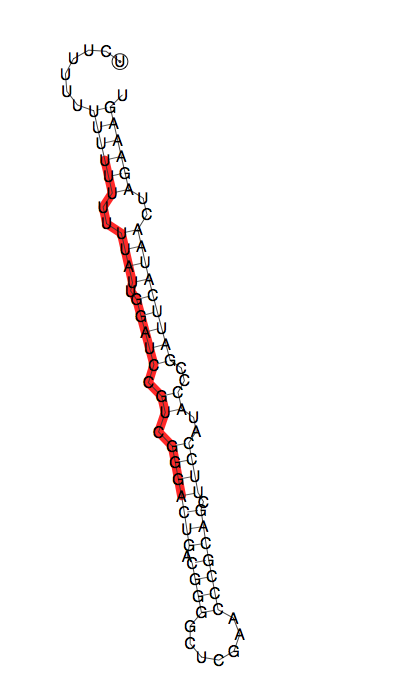


**Secondary structure for ‘va-miR008’**


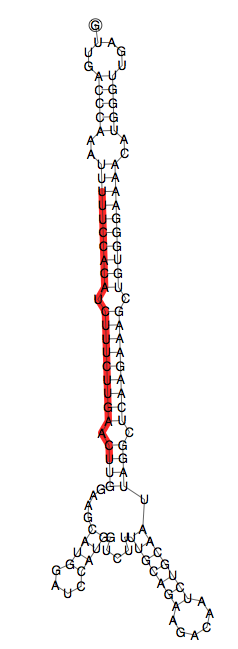


**Secondary structure for ‘va-miR009’**


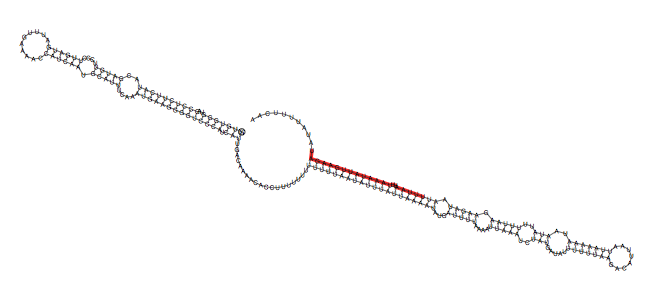


**Secondary structure for ‘va-miR010’**


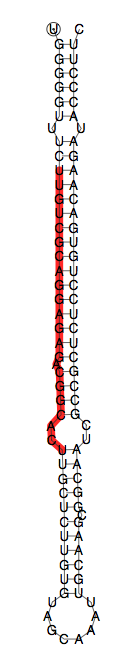


**Secondary structure for ‘va-miR011’**


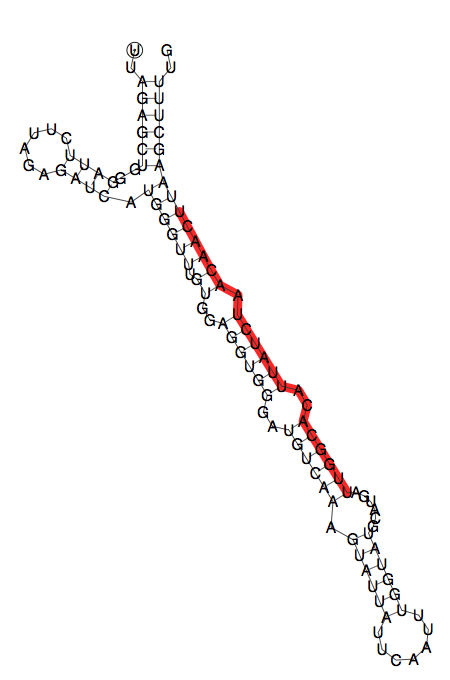


**Secondary structure for ‘va-miR012’**


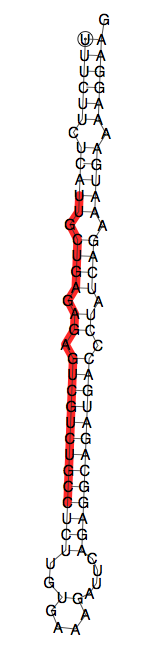


**Secondary structure for ‘va-miR013’**


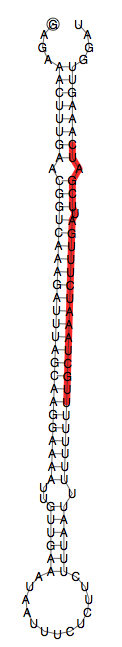


**Secondary structure for ‘va-miR014’**


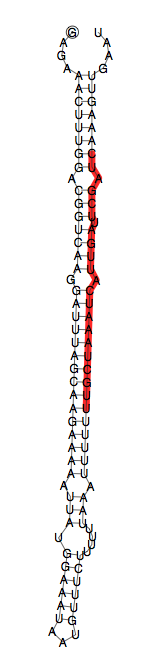


**Secondary structure for ‘va-miR015’**


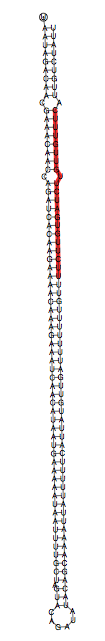


**Secondary structure for ‘va-miR016’**


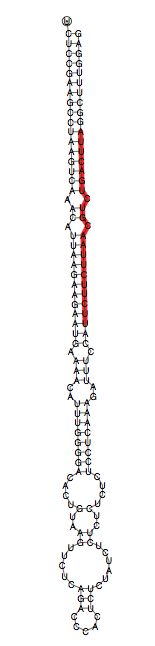


**Secondary structure for ‘va-miR017’**


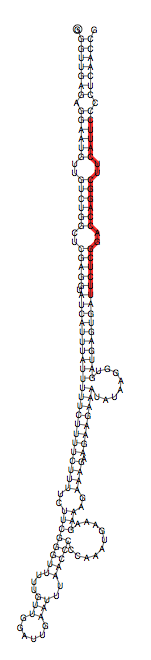


**Secondary structure for ‘va-miR018’**


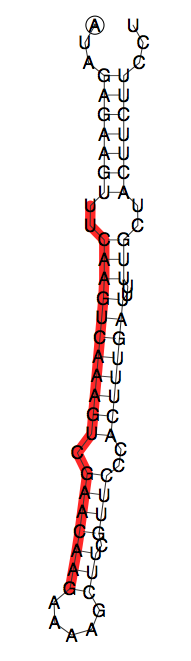


**Secondary structure for ‘va-miR019’**


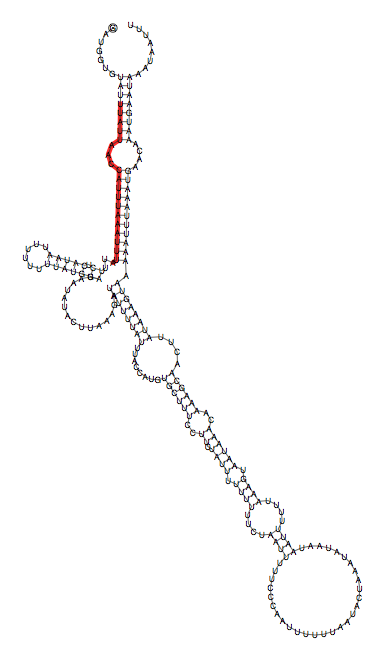


**Secondary structure for ‘va-miR020’**


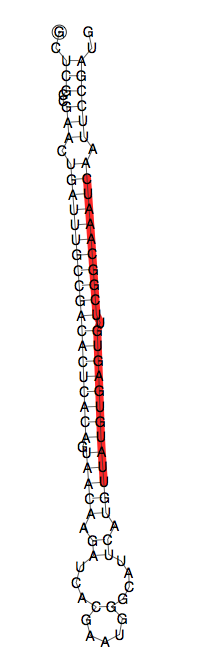


**Secondary structure for ‘va-miR021’**


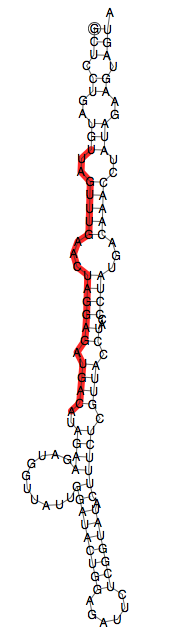


**Secondary structure for ‘va-miR022a’**


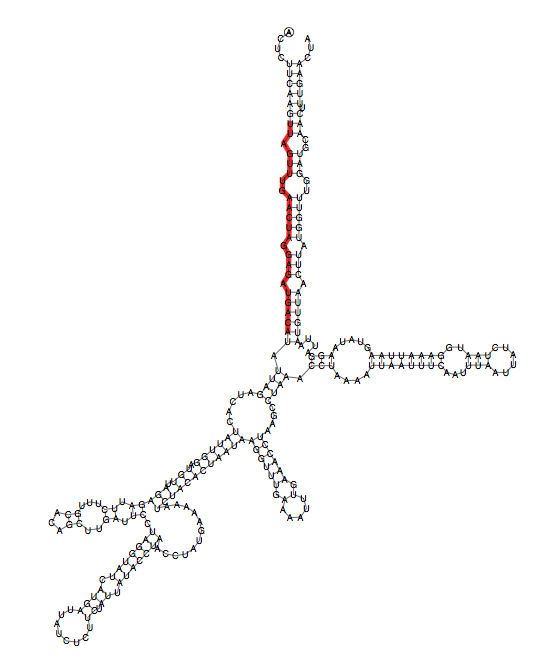


**Secondary structure for ‘va-miR022b’**


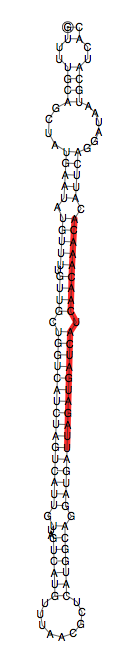


**Secondary structure for ‘va-miR023’**


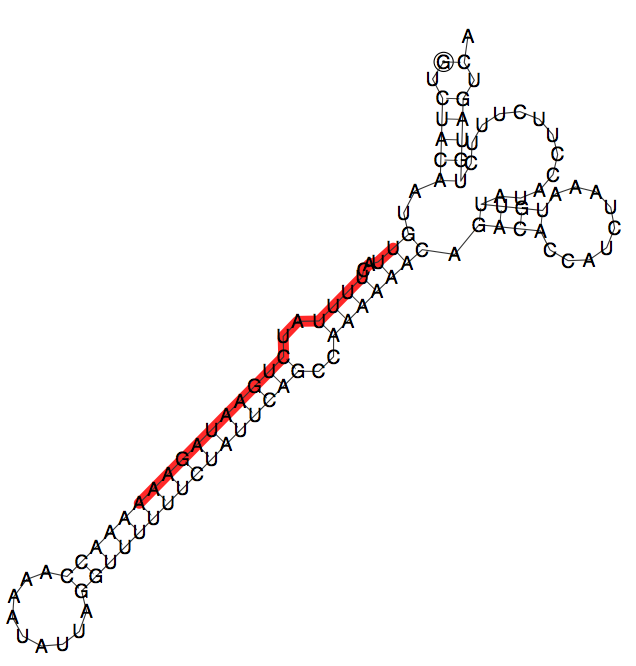


**Secondary structure for ‘va-miR024’**


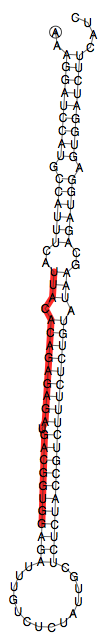


**Secondary structure for ‘va-miR025’**


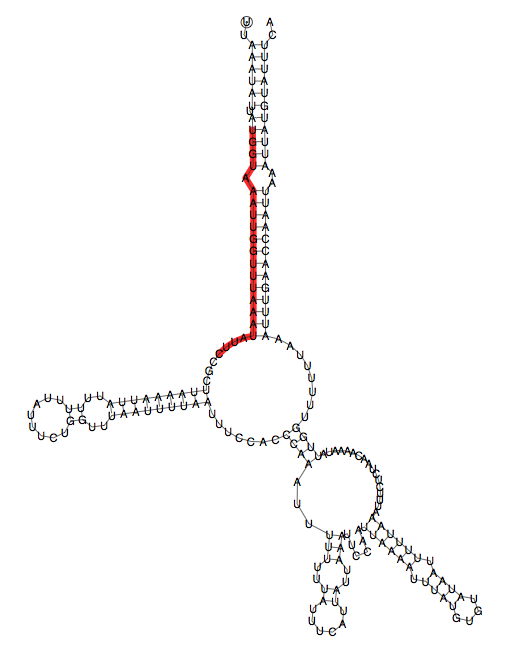


**Secondary structure for ‘va-miR026’**


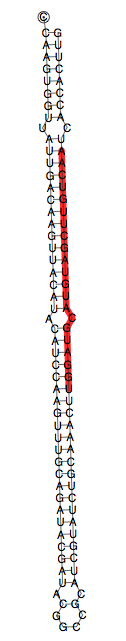


**Secondary structure for ‘va-miR027’**


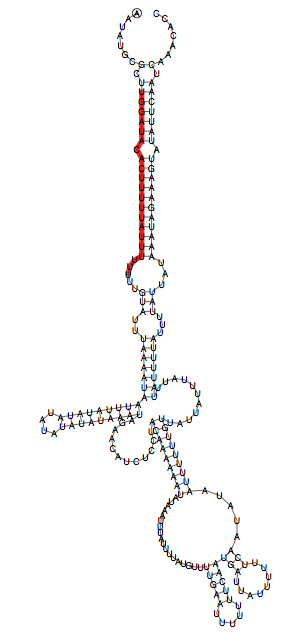


**Secondary structure for ‘va-miR028’**


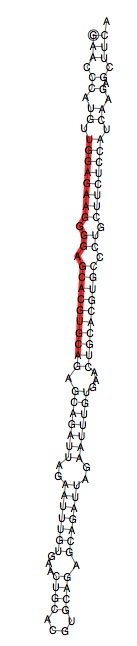


**Secondary structure for ‘va-miR029a’**


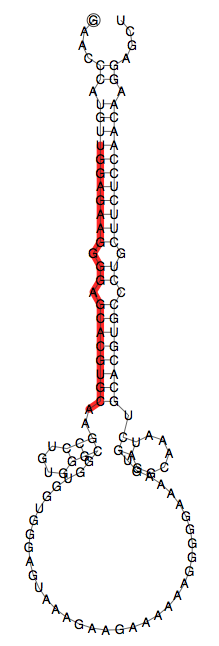


**Secondary structure for ‘va-miR029b’**


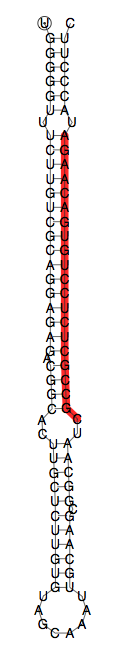


**Secondary structure for ‘va-miR030’**


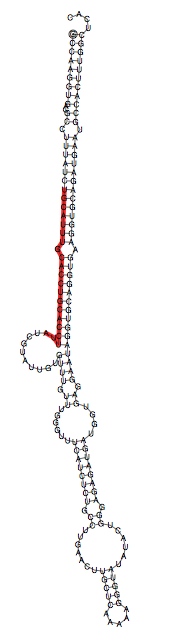


**Secondary structure for ‘va-miR031’**


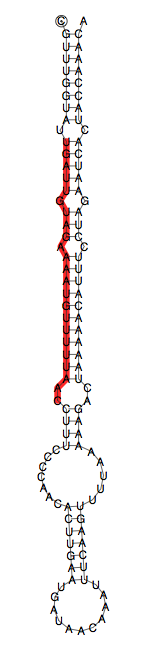


**Secondary structure for ‘va-miR032’**


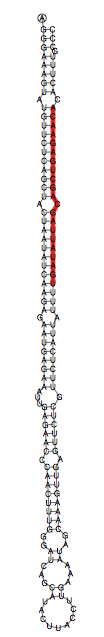


**Secondary structure for ‘va-miR033’**


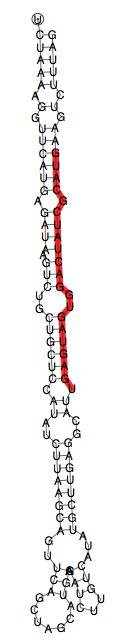


**Secondary structure for ‘va-miR034’**


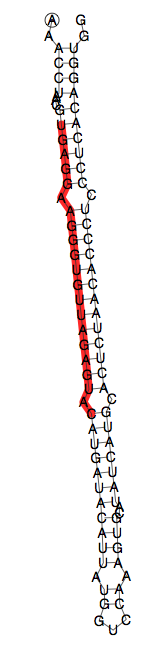


**Secondary structure for ‘va-miR035’**


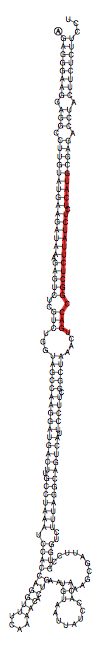


**Secondary structure for ‘va-miR036’**


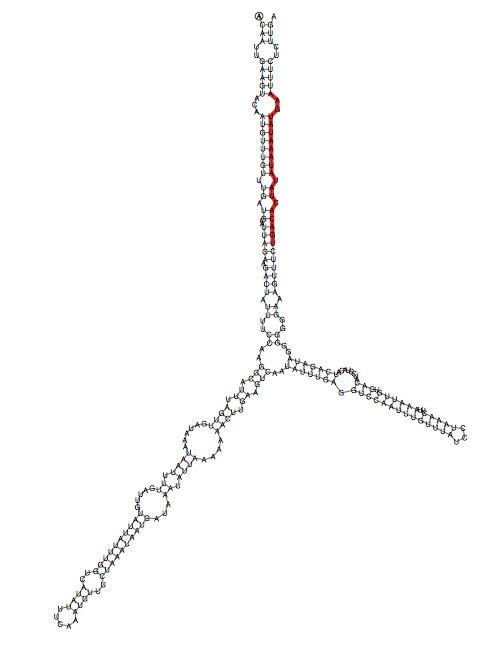


**Secondary structure for ‘va-miR037’**


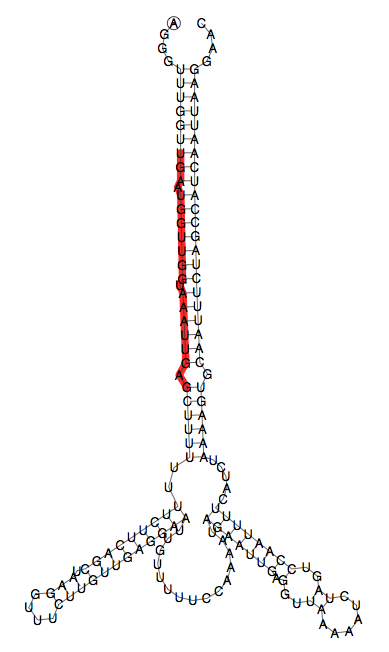


**Secondary structure for ‘va-miR038’**


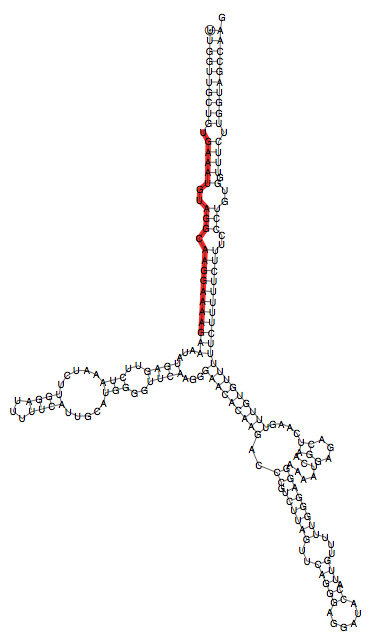


**Secondary structure for ‘va-miR039’**


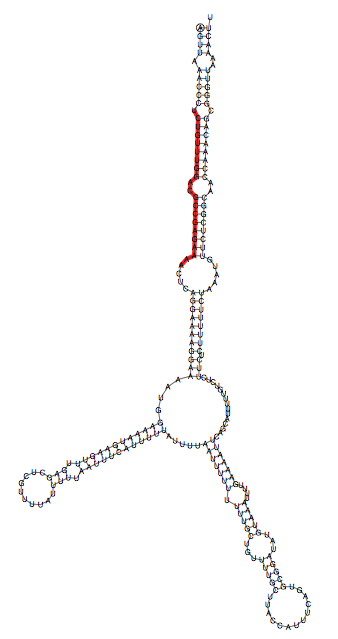


**Secondary structure for ‘va-miR040’**


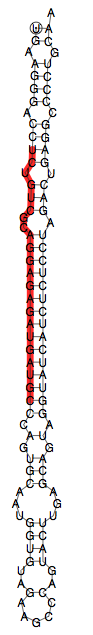


**Secondary structure for ‘va-miR041’**


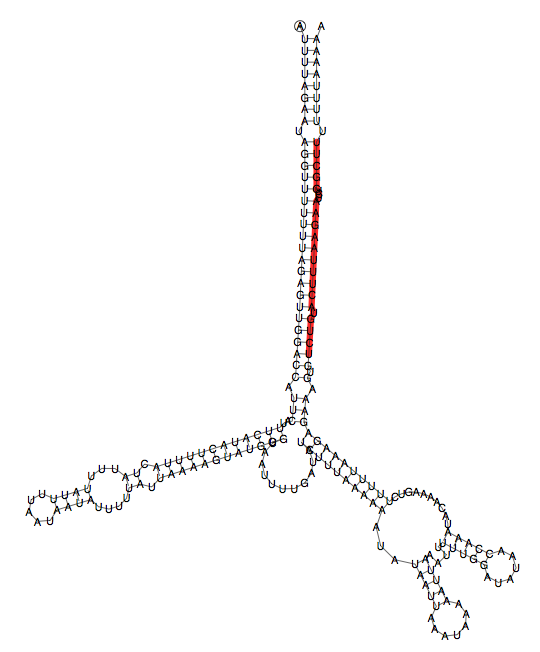


**Secondary structure for ‘va-miR042’**


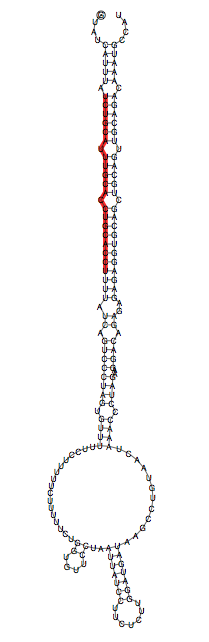


**Secondary structure for ‘va-miR043’**


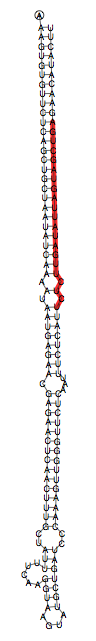


**Secondary structure for ‘va-miR044’**


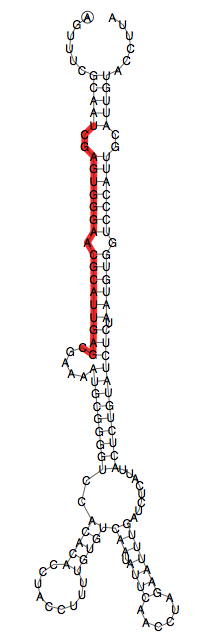


**Secondary structure for ‘va-miR045’**


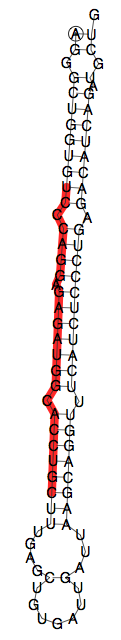


**Secondary structure for ‘va-miR046’**


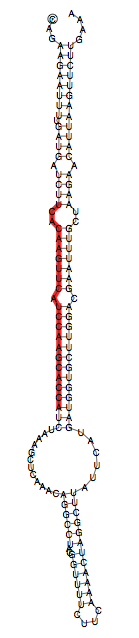


**Secondary structure for ‘va-miR047’**


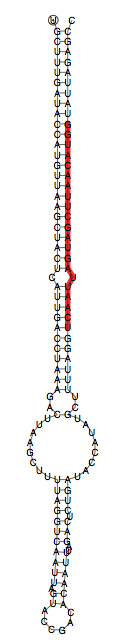


**Secondary structure for ‘va-miR048’**


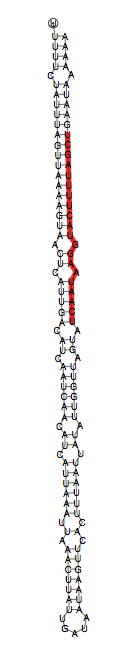


**Secondary structure for ‘va-miR049’**


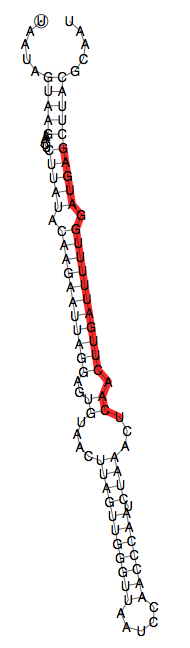


**Secondary structure for ‘va-miR050’**


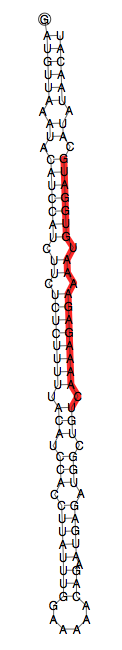


**Secondary structure for ‘va-miR051’**


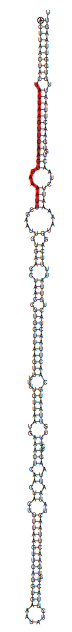


**Secondary structure for ‘va-miR052’**


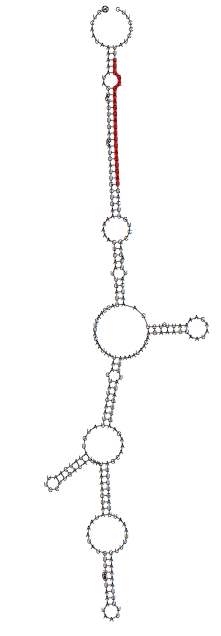


**Secondary structure for ‘va-miR053’**


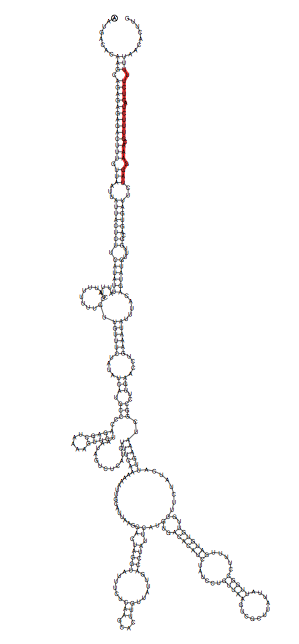


**Secondary structure for ‘va-miR054’**


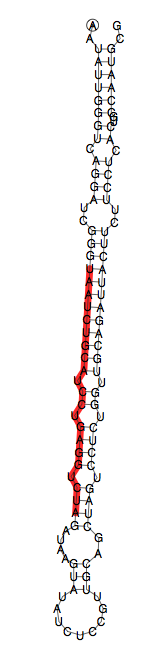


**Secondary structure for ‘va-miR055’**


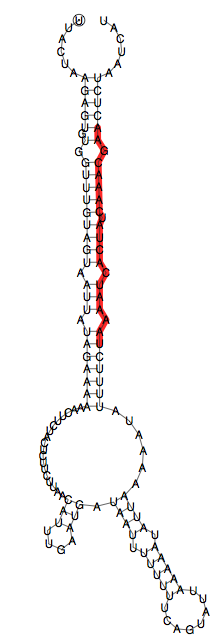


**Secondary structure for ‘va-miR056a’**


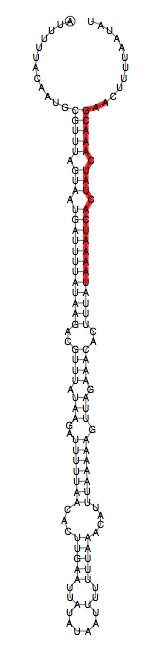


**Secondary structure for ‘va-miR056b’**


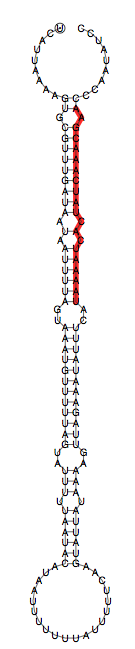


**Secondary structure for ‘va-miR056c’**


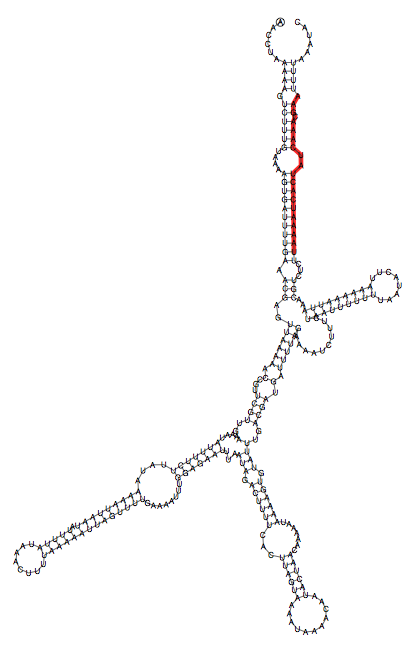


**Secondary structure for ‘va-miR056d’**


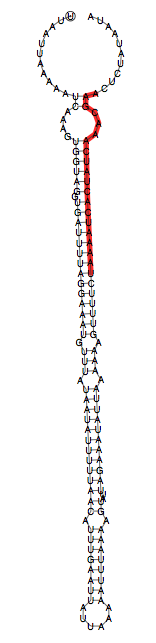


**Secondary structure for ‘va-miR056e’**


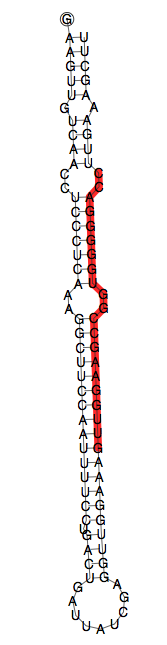


**Secondary structure for ‘va-miR057a’**


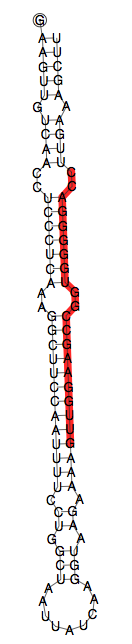


**Secondary structure for ‘va-miR057b’**


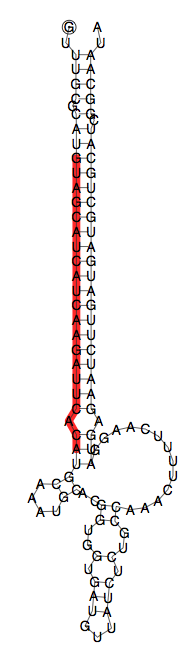


**Secondary structure for ‘va-miR058’**


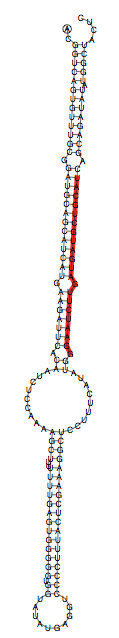


**Secondary structure for ‘va-miR059’**


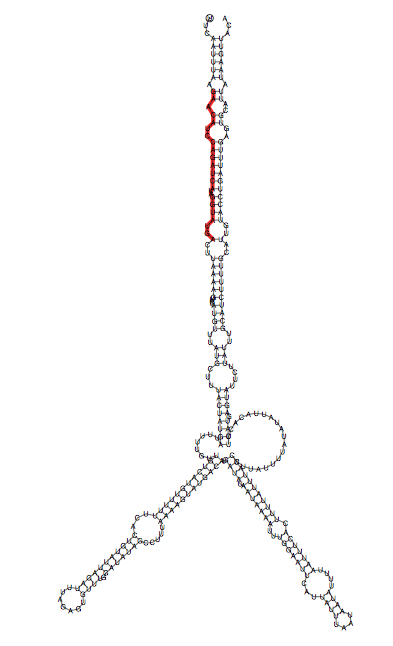


**Secondary structure for ‘va-miR060’**


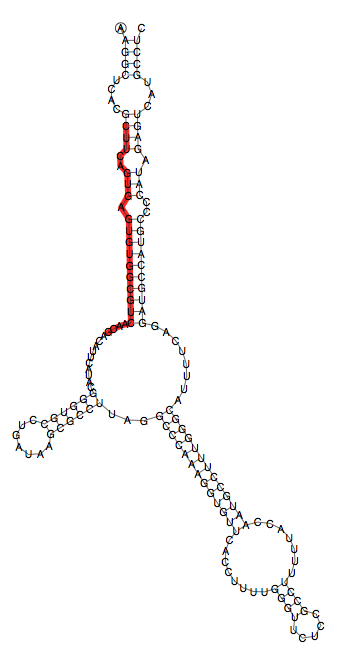


**Secondary structure for ‘va-miR061’**


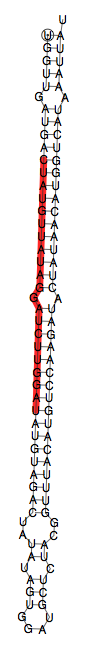


**Secondary structure for ‘va-miR062’**


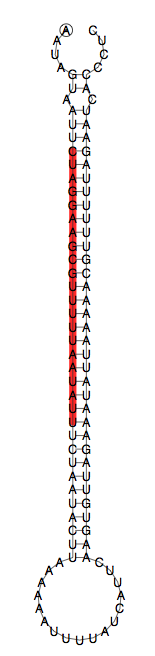


**Secondary structure for ‘va-miR063’**


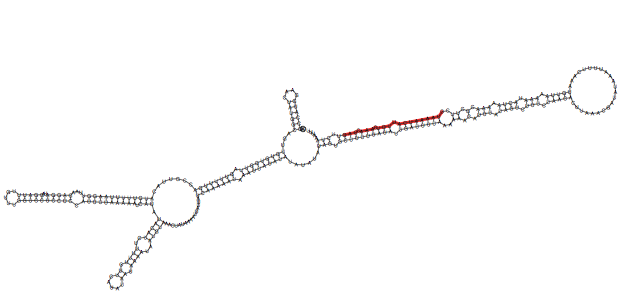


**Secondary structure for ‘va-miR064’**


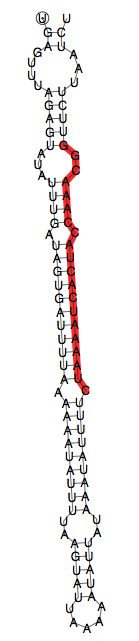


**Secondary structure for ‘va-miR065a’**


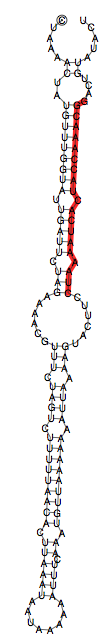


**Secondary structure for ‘va-miR066’**


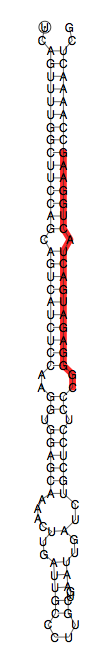


**Secondary structure for ‘va-miR067’**


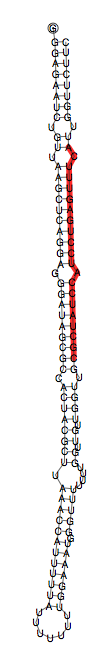


**Secondary structure for ‘va-miR068’**


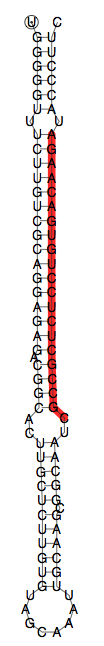


**Secondary structure for ‘va-miR069’**


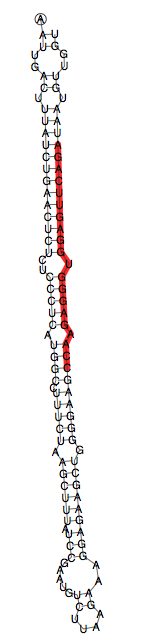


**Secondary structure for ‘va-miR070’**


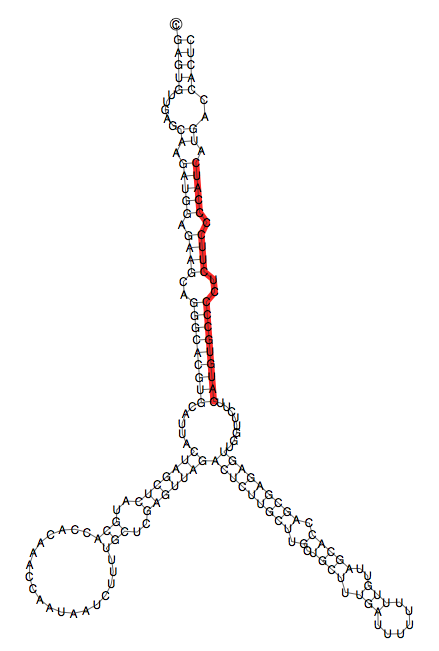


**Secondary structure for ‘va-miR071’**


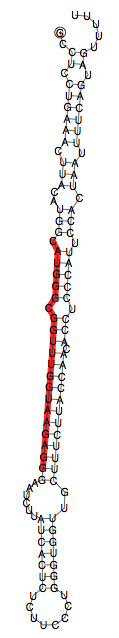


**Secondary structure for ‘va-miR072’**


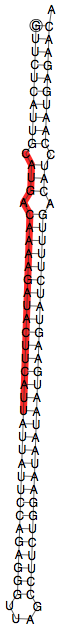


**Secondary structure for ‘va-miR073’**


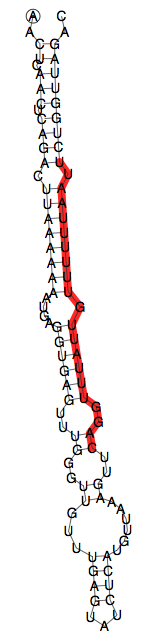


**Secondary structure for ‘va-miR074’**


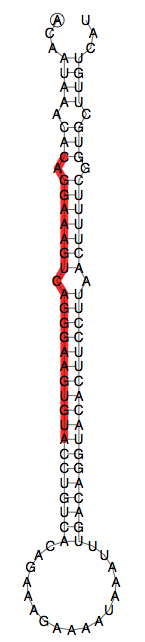


**Secondary structure for ‘va-miR075’**


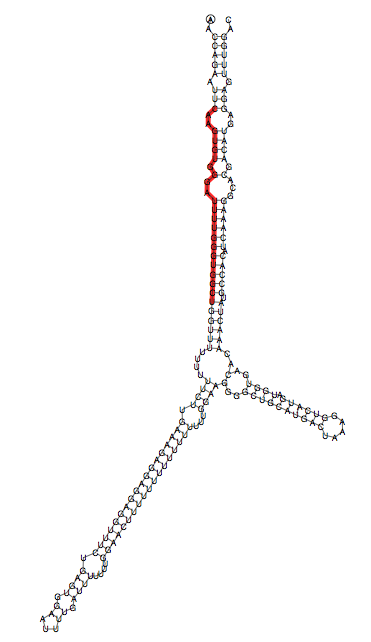


**Secondary structure for ‘va-miR076’**


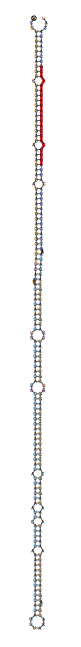


**Secondary structure for ‘va-miR077’**


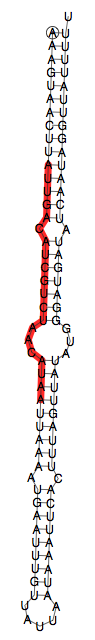


**Secondary structure for ‘va-miR078’**


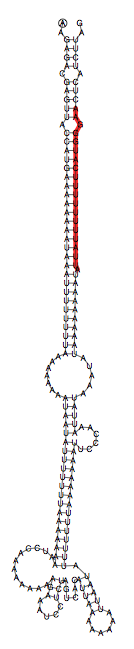


**Secondary structure for ‘va-miR079’**


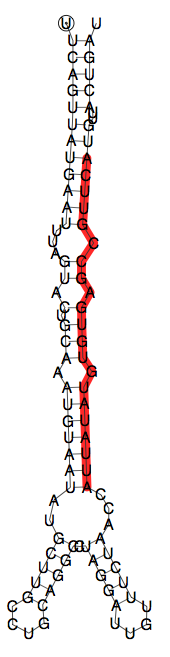


**Secondary structure for ‘va-miR080’**


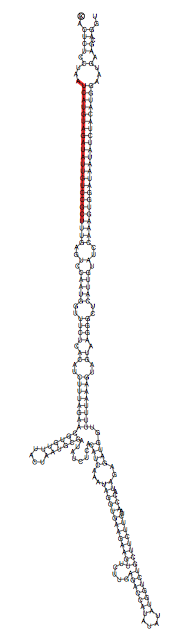


**Secondary structure for ‘va-miR081’**


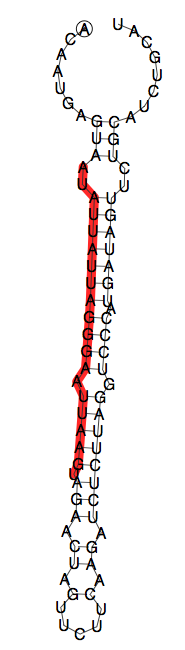


**Secondary structure for ‘va-miR082’**


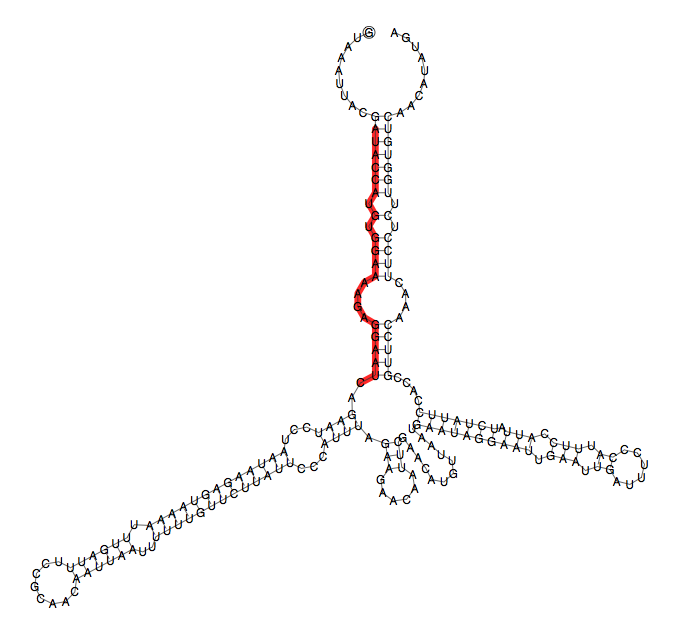


**Secondary structure for ‘va-miR083’**


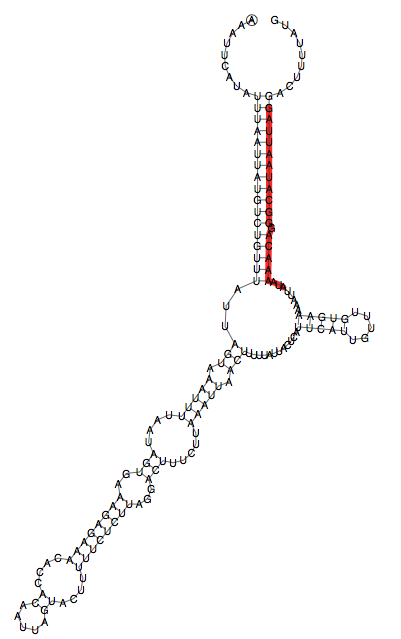


**Secondary structure for ‘va-miR084’**


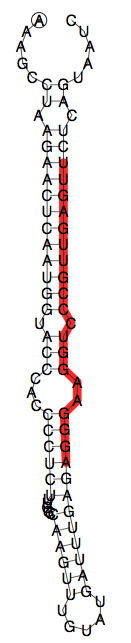


**Secondary structure for ‘va-miR085a’**


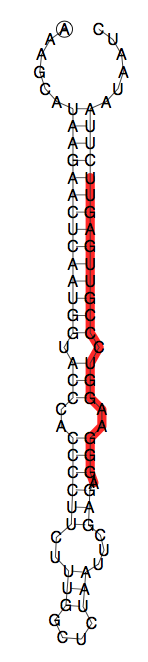


**Secondary structure for ‘va-miR085b’**


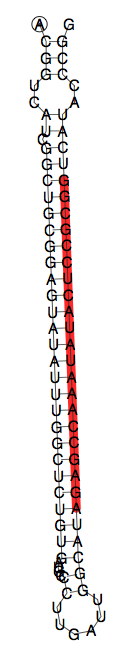


**Secondary structure for ‘va-miR086’**


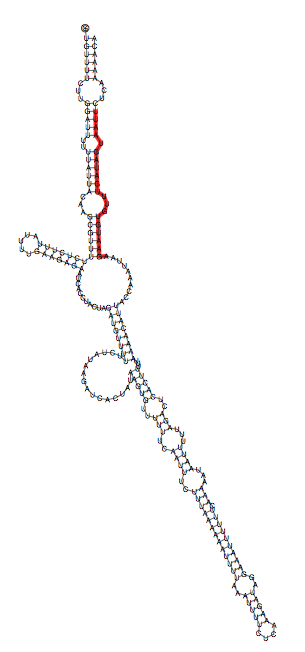


**Secondary structure for ‘va-miR087a’**


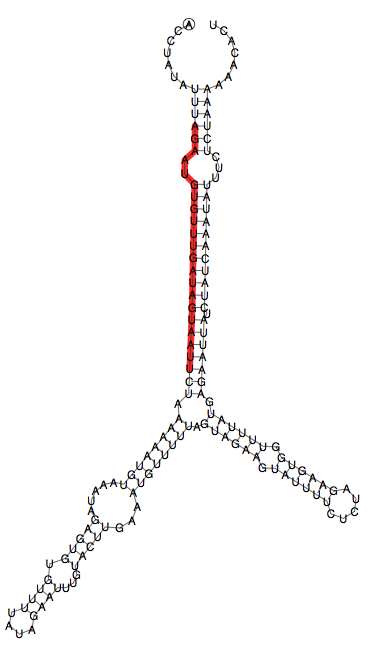


**Secondary structure for ‘va-miR087b’**


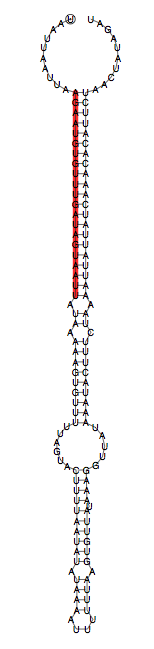


**Secondary structure for ‘va-miR087c’**


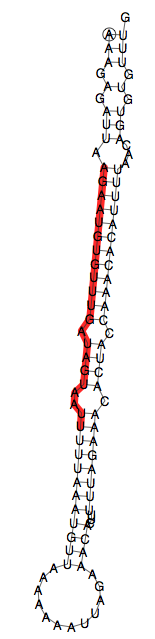


**Secondary structure for ‘va-miR087d’**

**Secondary structure for ‘va-miR088’**

**Secondary structure for ‘va-miR089’**

**Secondary structure for ‘va-miR090’**

**Secondary structure for ‘va-miR091’**

**Secondary structure for ‘va-miR092’**

**Secondary structure for ‘va-miR093’**

**Secondary structure for ‘va-miR094’**

**Secondary structure for ‘va-miR095’**

**Secondary structure for ‘va-miR096’**

**Secondary structure for ‘va-miR097’**

**Secondary structure for ‘va-miR098’**

**Secondary structure for ‘va-miR099a’**

**Secondary structure for ‘va-miR099b’**

**Secondary structure for ‘va-miR100’**

**Secondary structure for ‘va-miR101’**

**Secondary structure for ‘va-miR102’**

**Secondary structure for ‘va-miR103’**

**Secondary structure for ‘va-miR104’**

**Secondary structure for ‘va-miR105’**

**Secondary structure for ‘va-miR106’**
