# Supplementary figures and images for: Identification of microRNAs from Amur grape (vitis amurensis Rupr.) by deep sequencing and analysis of microRNA variations with bioinformatics
Source: BMC Genomics. 2012 Mar 29;13:122. doi: 10.1186/1471-2164-13-122 (PMC3353164; doi:10.1186/1471-2164-13-122)

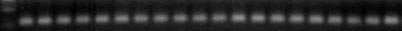

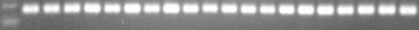


bp M 1 2 3 4 5 6 7 8 9 10 11 12 13 14 15 16 17 18 19 20

**A:** 100

50

**B:** 100

50

**C:** 100

50

**D:** 100

50


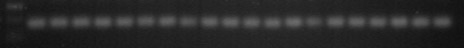

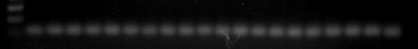


bp M 1’ 2’ 3’ 4’ 5’ 6’ 7’ 8’ 9’ 10’ 11’ 12’ 13’ 14’ 15’ 16’ 17’ 18’ 19’ 20’

Supplement: Additional file 4 — 3'RACE and 5' RACE products of Vv-miRNAs amplified by PCR shown in an ethidium bromide-stained agarose gel. Sizes of the molecular weight markers of the bottom and the second bottom bands are 50 bp and 100 bp on A, B, C and D, respectively. Lanes 1-20 are 3'RACE (A) and 5'RACE (B) products of 20 lower abundance va-miRNAs (va-miR398b, va-miR399c, va-miR828a, va-miR005, va-miR011, va-miR020, va-miR028, va-miR029, va-miR032, va-miR037, va-miR045, va-miR048, va-miR052, va-miR063, va-miR066, va-miR074, va-miR077, va-miR082, va-miR095 and va-miR098, respectively). Lanes 1'-20' are 3'RACE (C) and 5'RACE (D) products of 20 higher abundance va-miRNAs (va-miR156e, va-miR160c, va-miR162, va-miR164c, va-miR166c, va-miR169m, va-miR171c, va-miR172c, va-miR408, va-miR535a, va-miR001, va-miR007, va-miR016, va-miR018, va-miR023, va-miR046, va-miR047, va-miR049, va-miR057 and va-miR062, respectively). The sizes of 3'RACE products are about 83 bp while the size of 5'RACE products are about 57 bp. [file 1471-2164-13-122-S4.DOC]
