# Supplementary material for: Identification of microRNAs from Amur grape (vitis amurensis Rupr.) by deep sequencing and analysis of microRNA variations with bioinformatics
Source: BMC Genomics. 2012 Mar 29;13:122. doi: 10.1186/1471-2164-13-122 (PMC3353164; doi:10.1186/1471-2164-13-122)
Supplement: Additional file 5 — List of SNPs of conserved miRNAs in Amur grape. [file 1471-2164-13-122-S5.DOC]

| Table S2 | | | | |  |
| --- | --- | --- | --- | --- | --- |
| MiRNA ID | Base transition or transversion | Position of varied base | SNP count | Normal count | Rate （S/N） |
| va-miR156a | G->T | 15 | 38297 | 40946 | 93.53% |
| va-miR156b | A->G | 8 | 735 | 41204 | 1.78% |
| va-miR156b | G->T | 13 | 66 | 41204 | 0.16% |
| va-miR156b | T->G | 14 | 90 | 41204 | 0.22% |
| va-miR156c | A->G | 8 | 739 | 39298 | 1.88% |
| va-miR156c | G->T | 13 | 65 | 39298 | 0.17% |
| va-miR156c | T->G | 14 | 84 | 39298 | 0.21% |
| va-miR156d | A->G | 8 | 735 | 41907 | 1.75% |
| va-miR156d | G->T | 13 | 66 | 41907 | 0.16% |
| va-miR156d | T->G | 14 | 94 | 41907 | 0.22% |
| va-miR156e | G->A | 8 | 33604 | 34341 | 97.85% |
| va-miR156f | A->G | 17 | 37 | 26816 | 0.14% |
| va-miR156f | A->G | 6 | 1571 | 26816 | 5.86% |
| va-miR156f | A->T | 15 | 35 | 26816 | 0.13% |
| va-miR156g | A->G | 17 | 37 | 26751 | 0.14% |
| va-miR156g | A->G | 6 | 1571 | 26751 | 5.87% |
| va-miR156g | A->T | 15 | 35 | 26751 | 0.13% |
| va-miR156i | A->G | 17 | 37 | 26751 | 0.14% |
| va-miR156i | A->G | 6 | 1571 | 26751 | 5.87% |
| va-miR156i | A->T | 15 | 35 | 26751 | 0.13% |
| va-miR159c | C->T | 17 | 53 | 3567 | 1.49% |
| va-miR159c | G->T | 14 | 22 | 3567 | 0.62% |
| va-miR160a | A->T | 15 | 289 | 318 | 90.88% |
| va-miR160b | A->T | 15 | 289 | 318 | 90.88% |
| va-miR160c | T->A | 15 | 29 | 312 | 9.29% |
| va-miR160d | T->A | 15 | 28 | 318 | 8.81% |
| va-miR160e | A->T | 15 | 289 | 318 | 90.88% |
| va-miR160f | T->A | 15 | 28 | 318 | 8.81% |
| va-miR164a | C->T | 9 | 35 | 7031 | 0.50% |
| va-miR164a | G->T | 8 | 32 | 7031 | 0.46% |
| va-miR164c | C->T | 9 | 35 | 7071 | 0.50% |
| va-miR164c | G->T | 8 | 32 | 7071 | 0.45% |
| va-miR164d | C->T | 9 | 35 | 7031 | 0.50% |
| va-miR164d | G->T | 8 | 32 | 7031 | 0.46% |
| va-miR166a | A->C | 15 | 57 | 39758 | 0.14% |
| va-miR166a | A->C | 5 | 39 | 39758 | 0.10% |
| va-miR166a | C->T | 14 | 48 | 39758 | 0.12% |
| va-miR166a | C->T | 7 | 43 | 39758 | 0.11% |
| va-miR166b | A->C | 15 | 1870 | 216483 | 0.86% |
| va-miR166b | C->T | 14 | 384 | 216483 | 0.18% |
| va-miR166c | A->C | 15 | 5789 | 361920 | 1.60% |
| va-miR166c | C->A | 14 | 1028 | 361920 | 0.28% |
| va-miR166c | C->T | 6 | 348 | 361920 | 0.10% |
| va-miR166c | C->T | 7 | 371 | 361920 | 0.10% |
| va-miR166c | T->C | 17 | 439 | 361920 | 0.12% |
| va-miR166d | A->C | 15 | 5791 | 362609 | 1.60% |
| va-miR166d | C->A | 14 | 1028 | 362609 | 0.28% |
| va-miR166d | C->T | 6 | 348 | 362609 | 0.10% |
| va-miR166d | T->C | 17 | 439 | 362609 | 0.12% |
| va-miR166d | C->T | 7 | 372 | 362609 | 0.10% |
| va-miR166e | A->C | 15 | 5789 | 361920 | 1.60% |
| va-miR166e | C->A | 14 | 1028 | 361920 | 0.28% |
| va-miR166e | C->T | 6 | 348 | 361920 | 0.10% |
| va-miR166e | C->T | 7 | 371 | 361920 | 0.10% |
| va-miR166e | T->C | 17 | 439 | 361920 | 0.12% |
| va-miR166f | A->C | 15 | 5832 | 368092 | 1.58% |
| va-miR166f | C->A | 14 | 1034 | 368092 | 0.28% |
| va-miR166f | C->T | 6 | 357 | 368092 | 0.10% |
| va-miR166f | C->T | 7 | 377 | 368092 | 0.10% |
| va-miR166f | T->C | 17 | 441 | 368092 | 0.12% |
| va-miR166g | A->C | 15 | 5835 | 369314 | 1.58% |
| va-miR166g | C->A | 14 | 1035 | 369314 | 0.28% |
| va-miR166g | C->T | 6 | 359 | 369314 | 0.10% |
| va-miR166g | C->T | 7 | 377 | 369314 | 0.10% |
| va-miR166g | T->C | 17 | 441 | 369314 | 0.12% |
| va-miR166h | A->C | 15 | 5829 | 384867 | 1.51% |
| va-miR166h | C->A | 14 | 1037 | 384867 | 0.27% |
| va-miR166h | C->T | 6 | 365 | 384867 | 0.09% |
| va-miR166h | C->T | 7 | 398 | 384867 | 0.10% |
| va-miR166h | T->C | 17 | 439 | 384867 | 0.11% |
| va-miR167b | C->T | 10 | 331 | 107749 | 0.31% |
| va-miR167b | C->T | 9 | 215 | 107749 | 0.20% |
| va-miR167b | G->T | 5 | 135 | 107749 | 0.13% |
| va-miR167b | G->T | 8 | 118 | 107749 | 0.11% |
| va-miR167c | C->T | 10 | 193 | 79831 | 0.24% |
| va-miR167c | C->T | 6 | 79 | 79831 | 0.10% |
| va-miR167c | C->T | 9 | 193 | 79831 | 0.24% |
| va-miR167c | G->T | 5 | 112 | 79831 | 0.14% |
| va-miR167c | G->T | 8 | 87 | 79831 | 0.11% |
| va-miR167d | C->T | 10 | 112 | 36135 | 0.31% |
| va-miR167d | C->T | 9 | 73 | 36135 | 0.20% |
| va-miR167d | G->T | 5 | 50 | 36135 | 0.14% |
| va-miR167d | G->T | 8 | 41 | 36135 | 0.11% |
| va-miR167e | C->T | 10 | 327 | 107101 | 0.31% |
| va-miR167e | C->T | 9 | 216 | 107101 | 0.20% |
| va-miR167e | G->T | 5 | 135 | 107101 | 0.13% |
| va-miR167e | G->T | 8 | 118 | 107101 | 0.11% |
| va-miR168 | G->T | 8 | 81 | 66687 | 0.12% |
| va-miR168 | T->G | 15 | 79 | 66687 | 0.12% |
| va-miR168 | T->G | 9 | 73 | 66687 | 0.11% |
| va-miR169b | G->A | 14 | 752 | 1023 | 73.51% |
| va-miR169h | G->A | 14 | 752 | 1017 | 73.94% |
| va-miR169i | G->T | 16 | 750 | 753 | 99.60% |
| va-miR169l | A->G | 13 | 265 | 1043 | 25.41% |
| va-miR169l | C->T | 4 | 26 | 1043 | 2.49% |
| va-miR169m | C->T | 4 | 20 | 703 | 2.85% |
| va-miR169o | C->T | 4 | 20 | 713 | 2.81% |
| va-miR169p | C->T | 4 | 20 | 720 | 2.78% |
| va-miR169r | T->C | 5 | 685 | 1160 | 59.05% |
| va-miR169t | G->A | 13 | 6 | 23 | 26.09% |
| va-miR169u | T->C | 5 | 746 | 772 | 96.63% |
| va-miR171e | C->T | 12 | 2443 | 2512 | 97.25% |
| va-miR171f | C->T | 9 | 21 | 92 | 22.83% |
| va-miR319b | C->T | 17 | 9 | 87 | 10.34% |
| va-miR319e | C->T | 7 | 812 | 858 | 94.64% |
| va-miR396a | A->G | 7 | 15648 | 19524 | 80.15% |
| va-miR396b | A->G | 7 | 16116 | 20191 | 79.82% |
| va-miR396c | A->G | 7 | 15334 | 19824 | 77.35% |
| va-miR396d | A->G | 7 | 15334 | 20003 | 76.66% |
| va-miR399a | A->G | 13 | 13 | 77 | 16.88% |
| va-miR399b | G->A | 13 | 36 | 62 | 58.06% |
| va-miR399c | G->A | 13 | 38 | 63 | 60.32% |
| va-miR399e | T->A | 13 | 26 | 135 | 19.26% |
| va-miR399g | T->A | 13 | 25 | 64 | 39.06% |
| va-miR399h | A->G | 13 | 14 | 61 | 22.95% |
| va-miR479 | G->T | 10 | 24 | 22782 | 0.11% |
| va-miR479 | G->T | 11 | 36 | 22782 | 0.16% |
