# Supplementary material for: Identification of microRNAs from Amur grape (vitis amurensis Rupr.) by deep sequencing and analysis of microRNA variations with bioinformatics
Source: BMC Genomics. 2012 Mar 29;13:122. doi: 10.1186/1471-2164-13-122 (PMC3353164; doi:10.1186/1471-2164-13-122)
Supplement: Additional file 6 — List of miR-LDs of conserved miRNAs in Amur grape. [file 1471-2164-13-122-S6.DOC]

| Table S3 | | |  |
| --- | --- | --- | --- |
| MiRNAs ID | Edit type | Sequences (5'-3') | Length |
| va-miR156a | wild type | TGACAGAAGAGAGGGAGCAC | 20 |
|  | miR-LD | GACAGAAGAGAGGGAGCAC | 19 |
| va-miR156i | wild type | TGACAGAAGATAGAGAGCAC | 20 |
|  | miR-LD | GACAGAAGATAGAGAGCAC | 19 |
| va-miR166a | wild type | TCGGACCAGGCTTCATTCCTG | 21 |
|  | miR-LD | GGACCAGGCTTCATTCCTG | 19 |
| va-miE166c/e/h | wild type | TCGGACCAGGCTTCATTCCCCC | 21 |
|  | miR-LD | TCGGACCAGGCTTCATTCCCCCC | 22 |
| va-miR166d/f/g | wild type | TCGGACCAGGCTTCATTCCCCT | 21 |
|  | miR-LD | TCGGACCAGGCTTCATTCCCCTT | 22 |
| va-miR167a | wild type | TGAAGCTGCCAGCATGATCTG | 21 |
|  | miR-LD | TGAAGCTGCCAGCATGATCTGG | 22 |
| va-miR167b/e | wild type | TGAAGCTGCCAGCATGATCTA | 21 |
|  | miR-LD | TGAAGCTGCCAGCATGATCT | 20 |
| va-miR169b/h | wild type | TGAGCCAAGGATGGCTTGCCGT | 22 |
|  | miR-LD | TGAGCCAAGGATGGCTTGCCG | 21 |
| va-miR169i | wild type | TGAGCCAAGGATGACTGGCCGT | 22 |
|  | miR-LD | GAGCCAAGGATGACTGGCCGT | 21 |
| va-miR169l | wild type | TGAGCCAAGGATGACTTGCCG | 21 |
|  | miR-LD | GAGCCAAGGATGACTTGCCGT | 21 |
| va-miR169r | wild type | TGAGTCAAGGATGACTTGCCGA | 22 |
|  | miR-LD | TGAGTCAAGGATGACTTGCCG | 21 |
| va-miR169t | wild type | CGAGTCAAGGATGACTTGCCGA | 22 |
|  | miR-LD | CGAGTCAAGGATGACTTGCCG | 21 |
| va-miR171a | wild type | TGATTGAGCCGTGCCAATATC | 21 |
|  | miR-LD | TTGAGCCGTGCCAATATCATG | 21 |
| va-miR171c/d | wild type | TGATTGAGCCGTGCCAATATC | 21 |
|  | miR-LD | TTGAGCCGTGCCAATATCACG | 21 |
| va-miR171e | wild type | TTGAGCCGCGCCAATATCACT | 21 |
|  | miR-LD | TGATTGAGCCGCGCCAATATC | 21 |
| va-miR171i | wild type | TGATTGAGCCGTGCCAATATC | 21 |
|  | miR-LD | TTGAGCCGTGCCAATATCATG | 21 |
| va-miR319b | wild type | TTGGACTGAAGGGAGCTCCCT | 21 |
|  | miR-LD | CTTGGACTGAAGGGAGCTCCC | 21 |
| va-miR319c/f | wild type | TGCTTGGACTGAAGGGAG | 18 |
|  | miR-LD | CTTGGACTGAAGGGAGCTCCC | 21 |
| va-miR396a | wild type | TTCCACAGCTTTCTTGAA | 18 |
|  | miR-LD | TTCCACAGCTTTCTTGAACTA | 21 |
| va-miR396b | wild type | TTCCACAGCTTTCTTGAA | 19 |
|  | miR-LD | TTCCACAGCTTTCTTGAACT | 21 |
| va-miR396c/d | wild type | TTCCACAGCTTTCTTGAA | 21 |
|  | miR-LD | TTCCACAGCTTTCTTGAACTG | 21 |
| va-miR399a | wild type | CAAAGGAGAATTGCCCTGTTA | 21 |
|  | miR-LD | TGCCAAAGGAGAATTGCCCTG | 21 |
| va-miR399h | wild type | TGCCAAAGGAGAATTGCC | 18 |
|  | miR-LD | TGCCAAAGGAGAATTGCCCTG | 21 |
| va-miR403a/b/c/d/e/f | wild type | TTAGATTCACGCACAAACT | 19 |
|  | miR-LD | TTAGATTCACGCACAAACTCG | 21 |
| va-miR535a/b/c/d/e | wild type | TGACAACGAGAGAGAGCACGC | 21 |
|  | miR-LD | TGACAACGAGAGAGAGCACGCT | 22 |
