# Supplementary material for: Identification of microRNAs from Amur grape (vitis amurensis Rupr.) by deep sequencing and analysis of microRNA variations with bioinformatics
Source: BMC Genomics. 2012 Mar 29;13:122. doi: 10.1186/1471-2164-13-122 (PMC3353164; doi:10.1186/1471-2164-13-122)
Supplement: Additional file 7 — List of predicted target genes of non-conserved miRNAs identified in Amur grape. [file 1471-2164-13-122-S7.DOC]

| Table S4 | | | | |
| --- | --- | --- | --- | --- |
| MiRNA ID | Predicted target | Target genes annotion | Function | |
| miR001^a^ | GSVIVT00034955001 | Conserved gene of unknown function | Unknown | |
| miR002 | GSVIVT00029583001 | Conserved gene of unknown function | Unknown | |
|  | GSVIVT00030874001 | Conserved gene of unknown function | Unknown | |
|  | GSVIVT00015406001 | Two-component response regulator ARR8, expressed | Signal Transduction (ST) | |
|  | GSVIVT00027738001 | Fiber protein Fb2 | Growth and development(GD) | |
|  | GSVIVT00028656001 | NB-ARC domain containing protein | Stress response (SR) | |
| miR003 | GSVIVT00028338001 | Cell division control protein 50 | GD | |
|  | GSVIVT00000981001 | Peptidyl-prolyl cis-trans isomerase | Metablism (M) | |
|  | GSVIVT00027108001 | Rhomboid family protein | ST | |
|  | GSVIVT00003943001 | 2-oxoglutarate dehydrogenase E2 subunit | M | |
|  | GSVIVT00019344001 | Polypyrimidine track-binding protein homologue | (PTB) Splicing factor | |
|  | GSVIVT00015533001 | Rop guanine nucleotide exchange factor 1 | Transmembrane transport(TT) | |
|  | GSVIVT00018297001 | LIM domain protein WLIM2a | Transcription factor (TF) | |
|  | GSVIVT00036809001 | heterogeneous nuclear ribonucleoprotein | M | |
|  | GSVIVT00028139001 | FCA gamma | Flowering time | |
|  | GSVIVT00032536001 | Syntaxin-32 | Transport protein | |
|  | GSVIVT00024044001 | Rab3 GTPase-activating protein catalytic subuni | TT | |
|  | GSVIVT00030048001 | Phosphate translocator | M | |
|  | GSVIVT00020550001 | 50S ribosomal protein L13 | GD | |
|  | GSVIVT00001713001 | RAN binding protein | Unknown | |
|  | GSVIVT00015488001 | 26S proteasome non-ATPase regulatory subunit 3 | GD | |
|  | GSVIVT00036354001 | Protein RER1B |  | |
|  | GSVIVT00014575001 | CYP722A1 (cytochrome P450, family 722, subfamily A, polypeptide 1); oxygen binding | Unknown | |
|  | GSVIVT00038478001 | microtubule-associated protein | GD | |
|  | GSVIVT00006339001 | ankyrin repeat family protein | ST | |
|  | GSVIVT00015641001 | Phosphomevalonate kinase | M | |
|  | GSVIVT00006984001 | Peptidyl-prolyl cis-trans isomerase | M | |
|  | GSVIVT00021558001 | LETM1 domain containing protein | ST | |
|  | GSVIVT00027050001 | DNA-binding protein RHL1 | Transcript regulator (TR) | |
|  | GSVIVT00002814001 | RW1 | Unknown | |
|  | GSVIVT00025354001 | Rhodanese | M | |
|  | GSVIVT00037689001 | Methyltransferase 1 | GD | |
|  | GSVIVT00014410001 | DUF668 domain containing protein | Putative TAP | |
|  | GSVIVT00015692001 | GARP-like putative transcription factor KANADI1 (KANADI protein) | TF | |
|  | GSVIVT00021322001 | Phantastica | Unknown | |
|  | GSVIVT00030977001 | WD40 | TF; up-regulate anthocyanins biosynthesis | |
|  | GSVIVT00010969001 | SNF2 domain-containing protein / helicase domain-containing protein / RING finger domain-containing protein | TR | |
|  | GSVIVT00038542001 | Proline iminopeptidase | M | |
|  | GSVIVT00025546001 | Putative WD-repeat protein (WD-repeat protein) | TF | |
| miR004 | GSVIVT00037075001 | Conserved gene of unknown function | Unknown | |
|  | GSVIVT00001420001 | CM0216.350.nc protein | Unknown | |
|  | GSVIVT00006686001 | YALI0A20108p | Unknown | |
|  | GSVIVT00015488001 | 26S proteasome non-ATPase regulatory subunit 3 | GD | |
|  | GSVIVT00023250001 | Dual specificity protein phosphatase | M | |
|  | GSVIVT00034556001 | Cytochrome c oxidase subunit Vb | M | |
|  | GSVIVT00033078001 | Bet_v_I domain containing protein | SR (disease stress) | |
| miR005 | no |  |  | |
| miR006 ^a^ -1,-2,-3 | GSVIVT00027233001 | EDM2; transcription factor | SR; transcription factor | |
|  | GSVIVT00025927001 | CM0545.540.nc protein | Unknown | |
|  | GSVIVT00015048001 | Aux/IAA protein | TR | |
|  | GSVIVT00004903001 | Conserved gene of unknown function | Unknown | |
|  | GSVIVT00018490001 | Actin-1 | GD | |
|  | GSVIVT00003943001 | 2-oxoglutarate dehydrogenase E2 subunit | M | |
|  | GSVIVT00014437001 | HVA22 I | SR | |
|  | GSVIVT00034208001 | RWD domain-containing protein | Tryptophan metabolism | |
|  | GSVIVT00024774001 | Acyl-activating enzyme 18 | M | |
|  | GSVIVT00018612001 | ADP, ATP carrier | Transport protein | |
|  | GSVIVT00011815001 | Isoform 2 of AP2-like ethylene-responsive transcription factor AIL6 | TF | |
|  | GSVIVT00027891001 | TOM (Target of myb1) | TR | |
|  | GSVIVT00003764001 | serine/threonine protein kinase | SR | |
|  | GSVIVT00034137001 | Glycylpeptide N-tetradecanoyltransferase | Unknown | |
|  | GSVIVT00031148001 | UPF0424 protein | Unknown | |
|  | GSVIVT00014578001 | 50S ribosomal protein L31 | GD | |
|  | GSVIVT00001941001 | FAR1; Polynucleotidyl transferase, Ribonuclease H fold | M | |
|  | GSVIVT00024290001 | Catalytic/ hydrolase | M | |
|  | GSVIVT00027978001 | Conserved gene of unknown function | Unknown | |
|  | GSVIVT00000554001 | CG1676-PA | Unknown | |
|  | GSVIVT00001420001 | CM0216.350.nc protein | Unknown | |
|  | GSVIVT00034392001 | Ribosomal protein L37 | M | |
|  | GSVIVT00005430001 | Cleavage stimulation factor | GD | |
|  | GSVIVT00027847001 | integral membrane protein | ST | |
|  | GSVIVT00016078001 | Carbonic anhydrase | M | |
|  | GSVIVT00005657001 | S-adenosylmethionine-dependent methyltransferase | GD | |
|  | GSVIVT00032132001 | Derlin-1 | Unknown | |
|  | GSVIVT00037742001 | Heavy metal transport/detoxification protein | Transport protein | |
|  | GSVIVT00021217001 | Brassinosteroid biosynthetic protein LKB | GD | |
|  | GSVIVT00014593001 | Protein RER1B | SR (drought stress, salt stress) | |
|  | GSVIVT00028334001 | endoplasmic reticulum oxidoreductin | M | |
|  | GSVIVT00025980001 | 3-oxoacyl-[acyl-carrier-protein] synthase, mitochondrial | M | |
|  | GSVIVT00007438001 | GRF1-interacting factor 1 (GIF1) | TR | |
|  | GSVIVT00027646001 | GTP-binding protein signal |  | |
|  | GSVIVT00037944001 | Aspartokinase | M | |
|  | GSVIVT00038245001 | Chloroplastic group IIB intron splicing facilitator CRS2-B, chloroplastic | Splicing function | |
|  | GSVIVT00028178001 | Serine/threonine protein kinase | SR | |
|  | GSVIVT00024475001 | Phosphatidylinositol transfer protein | ST | |
|  | GSVIVT00024429001 | Kinesin, motor region | Unknown | |
|  | GSVIVT00029907001 | Vacuolar invertase 1, GIN1 | Hexose metablism | |
|  | GSVIVT00032286001 | MTD1 | TT | |
|  | GSVIVT00029302001 | Transcription factor | TF | |
|  | GSVIVT00021374001 | Prf interactor 30137 | SR (disease stress) | |
|  | GSVIVT00007695001 | Triacylglycerol lipase 1 | M | |
|  | GSVIVT00012951001 | Conserved gene of unknown function | Unknown | |
|  | GSVIVT00015295001 | TGA-type basic leucine zipper protein TGA1.1 | TF | |
|  | GSVIVT00027038001 | Conserved gene of unknown function | Unknown | |
|  | GSVIVT00010318001 | Conserved gene of unknown function | Unknown | |
|  | GSVIVT00003816001 | Protein kinase | M | |
|  | GSVIVT00025109001 | CBS domain containing protein | SR (defense response) | |
|  | GSVIVT00015488001 | 26S proteasome non-ATPase regulatory subunit 3 | GD | |
|  | GSVIVT00035484001 | cleavage and polyadenylation specificity factor; 72745-70039 | Splicing function | |
|  | GSVIVT00036354001 | Protein RER1B | SR (drought stress, salt stress) | |
|  | GSVIVT00028138001 | DNA-binding WRKY domain-containing protein | TF | |
|  | GSVIVT00038478001 | microtubule-associated protein | GD | |
|  | GSVIVT00029599001 | pyrophosphate-energized membrane proton pump 3 | Energy metablism | |
|  | GSVIVT00015093001 | Basic salivary proline-rich protein 2 metablism | Unknown | |
|  | GSVIVT00007092001 | Conserved gene of unknown function | Unknown | |
|  | GSVIVT00030293001 | Methionine aminopeptidase | GD | |
|  | GSVIVT00015612001 | Conserved gene of unknown function | Unknown | |
|  | GSVIVT00015641001 | Phosphomevalonate kinase | M | |
|  | GSVIVT00010223001 | Ribosomal protein L3 | Unknown | |
|  | GSVIVT00035505001 | cleavage and polyadenylation specificity factor; 72745-70039 | Splicing function | |
|  | GSVIVT00037686001 | Transporter | Transport function | |
|  | GSVIVT00034378001 | Dynein light chain | GD | |
|  | GSVIVT00031118001 | Cytidine/deoxycytidylate deaminase family protein | M | |
|  | GSVIVT00020713001 | Serine/threonine protein kinase | SR | |
|  | GSVIVT00005279001 | integral membrane protein | ST | |
|  | GSVIVT00027921001 | rhamnose biosynthetic enzyme 1 | M | |
|  | GSVIVT00016737001 | Subtilisin | Unknown | |
|  | GSVIVT00020931001 | Conserved gene of unknown function | Unknown | |
|  | GSVIVT00031097001 | MAK16 RBM13 | RNA binding motif protein 13 | |
|  | GSVIVT00037663001 | Signal peptide peptidase-like 3 | ST | |
|  | GSVIVT00014028001 | TOM (Target of myb1) | TR | |
|  | GSVIVT00019655001 | endo-1,3-beta-glucosidase | M | |
|  | GSVIVT00026114001 | Rab geranylgeranyl transferase type II beta subunit | M | |
|  | GSVIVT00024556001 | T3B23.2/T3B23.2 | Unknown | |
|  | GSVIVT00006512001 | Spermine synthase | GD | |
|  | GSVIVT00033847001 | STY-L protein | TF | |
|  | GSVIVT00007875001 | Conserved gene of unknown function | Unknown | |
|  | GSVIVT00025615001 | NADP-thioredoxin reductase C | GD | |
|  | GSVIVT00031250001 | Conserved gene of unknown function | Unknown | |
|  | GSVIVT00014052001 | Conserved gene of unknown function | Unknown | |
|  | GSVIVT00024903001 | RER1A protein | SR | |
|  | GSVIVT00019822001 | Homology of unknown gene | Unknown | |
|  | GSVIVT00032370001 | Conserved gene of unknown function | Unknown | |
|  | GSVIVT00025621001 | Nonsense-mediated mRNA decary protein 3 | Unknown | |
|  | GSVIVT00008891001 | VAP27-2 (VAMP/SYNAPTOBREVIN-ASSOCIATED PROTEIN 27-2); structural molecule | Unknown | |
|  | GSVIVT00011306001 | Acidic ribosomal protein P1a | Unknown | |
|  | GSVIVT00018790001 | FtsH Pftf | ST | |
|  | GSVIVT00013119001 | replication protein A1 (RPA1) stress response | SR (disease stress) | |
|  | GSVIVT00003059001 | Fiber protein Fb2 | GD | |
|  | GSVIVT00001852001 | Eukaryotic peptide chain release factor subunit 1-1 | M | |
|  | GSVIVT00012226001 | integral membrane protein | ST | |
|  | GSVIVT00022840001 | ATP/GTP/Ca++ binding protein | ST | |
|  | GSVIVT00023664001 | GTPase | GD | |
|  | GSVIVT00013110001 | Conserved gene of unknown function | Unknown | |
|  | GSVIVT00030977001 | WD40 | TF (anthocyanins biosynthesis) | |
|  | GSVIVT00028392001 | Multidrug resistance-associated protein 13 | SR | |
|  | GSVIVT00024773001 | Acyl-activating enzyme 18 | M | |
|  | GSVIVT00016333001 | Protein binding protein | Unknown | |
|  | GSVIVT00011637001 | Elongator component | Unknown | |
|  | GSVIVT00016021001 | Spindle_Spc25 domain containing protein | Unknown | |
| miR007^b^ | no |  |  | |
| miR008 ^a^ | GSVIVT00024729001* | E3 ubiquitin-protein ligase SINAT2 | M | |
|  | GSVIVT00023809001* | Type IIB calcium ATPase | ST | |
| miR009 | GSVIVT00034662001 | GRAM domain containing protein | TF (Disease resistence) | |
|  | GSVIVT00015541001 | ATP-binding domain-containing protein 3 | Unknown | |
| miR010 ^a^ | GSVIVT00014287001 | Vacuolar protein sorting-associated protein 45 homolog | ST | |
|  | GSVIVT00029503001 | SAC3/GANP family protein | GD | |
| miR011^b^ | no |  |  | |
| miR012 | no |  |  | |
| miR013 | no |  |  | |
| miR014 | no |  |  | |
| miR015 | GSVIVT00022215001 | NB-ARC domain containing protein | SR (disease stress) | |
| miR016 | no |  |  | |
| miR017 | no |  |  | |
| miR018 ^a^ | no |  |  | |
| miR019 | GSVIVT00006200001 | Dev_Cell_Death domain containing protein | Cell death | |
| miR020 | no |  |  | |
| miR021^b^ | no |  |  | |
| mir022-1,-2 | no |  |  | |
| miR023^b^ | GSVIVT00001081001* | SPX (SYG1/Pho81/XPR1) domain-containing protein | TT | |
| miR024 | GSVIVT00019134001 | Enolase_N domain containing protein | M | |
| miR025^b^ | no |  |  | |
| miR026 | no |  |  | |
| miR027 ^a^ | no |  |  | |
| miR028 | GSVIVT00017553001 | PHD-finger family protein | TR | |
| miR029^b^-1,  -2 | GSVIVT00026778001 | (+)-neomenthol dehydrogenase | M | |
| miR031^b^ | GSVIVT00020140001 | Plus-3 domain containing protein | SR | |
| miR032 | no |  |  | |
| miR033 | no |  |  | |
| miR034^b^ | no |  |  | |
| miR035 | no |  |  | |
| miR036^b^ | no |  |  | |
| miR037 | GSVIVT00000182001 | Conserved gene of unknown function | Unknown | |
|  | GSVIVT00018085001 | Conserved gene of unknown function | Unknown | |
|  | GSVIVT00038166001 | Conserved gene of unknown function | Unknown | |
| miR038 | GSVIVT00014751001 | Conserved gene of unknown function | Unknown | |
|  | GSVIVT00006016001 | Copine III | ST | |
| miR039^b^ | no |  |  | |
| miR040 | GSVIVT00007720001 | Conserved gene of unknown function | Unknown | |
|  | GSVIVT00003434001 | 3-beta hydroxysteroid dehydrogenase/isomerase | M | |
|  | GSVIVT00022802001 | F-box protein | TF (disease resistance) | |
| miR041^b^ | no |  |  | |
| miR042 | no |  |  | |
| miR043 | GSVIVT00011508001 | Subtilisin-like serine protease | GD | |
|  | GSVIVT00035102001 | Exonuclease | Unknown | |
|  | GSVIVT00020140001 | Plus-3 domain containing protein | Unknown | |
| miR044 | no |  |  | |
| miR045 | GSVIVT00019010001 | Conserved gene of unknown function | Unknown | |
|  | GSVIVT00010371001 | Conserved gene of unknown function | Unknown | |
| miR046 ^b^ | no |  |  | |
| miR047 ^a^ | no |  |  | |
| mir048 | no |  |  | |
| miR049 ^b^ | GSVIVT00015910001* | TPR repeat-containing protein | SR (salt stress, ABA) | |
| miR050 | GSVIVT00025692001 | NB-ARC domain containing protein | SR (disease resistence) | |
|  | GSVIVT00035762001 | NB-ARC domain containing protein | SR (disease resistence) | |
|  | GSVIVT00025670001 | NB-ARC domain containing protein | SR (disease resistence) | |
|  | GSVIVT00035785001 | NB-ARC domain containing protein | SR (disease resistence) | |
|  | GSVIVT00003433001 | Mini-chromosome maintenance protein MCM3 | ST | |
|  | GSVIVT00012138001 | NB-ARC domain containing protein | SR (disease resistence) | |
|  | GSVIVT00035780001 | NB-ARC domain containing protein | SR (disease resistence) | |
|  | GSVIVT00034023001 | EDA25 (embryo sac development arrest 25); binding | GD | |
| miR051 | no |  |  | |
| miR052 | GSVIVT00038611001 | Pyruvate kinase | M | |
| miR053 | GSVIVT00027623001 | Conserved gene of unknown function | Unknown | |
|  | GSVIVT00025865001 | EDM2; transcription factor | TF (defense response) | |
|  | GSVIVT00026247001 | Splicing factor | Splicing function | |
|  | GSVIVT00014931001 | Cytochrome b5 DIF-F (Cytochrome B5) | M (anthocyanins sysnthesis) | |
|  | GSVIVT00034043001 | Nuclear pore protein 84/107 containing protein | Unknown | |
|  | GSVIVT00008983001 | Conserved gene of unknown function | Unknown | |
|  | GSVIVT00015828001 | Peroxidase 20 | SR(starvation stress) | |
|  | GSVIVT00023147001 | gene of unknown function | Unknown | |
| miR054 ^b^ | GSVIVT00032397001* | GF24975 | Unknown | |
|  | GSVIVT00014342001* | DNAJ heat shock N-terminal domain-containing protein | Unknown | |
|  | GSVIVT00023147001* | Gene of unknown function | Unknown | |
| miR055 ^b^ | no |  |  | |
| miR056-1,-2,-3,-4,-5 | GSVIVT00028380001 | Beta-1,3-glucanase | M | |
|  | GSVIVT00021349001 | WD-repeat membrane protein | ST,TR | |
|  | GSVIVT00029308001 | Tomosyn | Regulator | |
|  | GSVIVT00028801001 | unknown |  | |
|  | GSVIVT00023147001 | unknown |  | |
|  | GSVIVT00010406001 | U3 small nucleolar RNA-associated protein 18 homolog | GD | |
| miR057 ^b^ -1,-2 | no |  |  | |
| miR058 | GSVIVT00009152001 | RNA polymerase III subunit | Unknown | |
| miR059 ^a^ | GSVIVT00022897001 | Conserved gene of unknown function | Unknown | |
|  | GSVIVT00017835001 | Conserved gene of unknown function | Unknown | |
|  | GSVIVT00024453001 | Transcription factor AP2D23 | TF | |
|  | GSVIVT00023070001 | PHAP2B protein TF | TF | |
|  | GSVIVT00009709001 | Conserved gene of unknown function | Unknown | |
|  | GSVIVT00019538001 | PHAP2A protein TF | TF | |
|  | GSVIVT00018277001 | PHAP2A protein TF | TF | |
| miR060 | no |  |  | |
| miR061 | no |  |  | |
| mir062 | no |  |  | |
| miR063 ^b^ | GSVIVT00026247001 | Splicing factor | Splicing function | |
| miR064 | no |  |  | |
| miR065-1,-2 | GSVIVT00028380001 | Beta-1,3-glucanase | M | |
|  | GSVIVT00025865001 | EDM2; transcription factor | TF (defense response) | |
|  | GSVIVT00021349001 | WD-repeat membrane protein | TF (stress response) | |
|  | GSVIVT00032174001 | MYB transcription factor MYB127 TF | TF (stress response) | |
|  | GSVIVT00026343001 | NAD-dependent epimerase/dehydratase metablism | M | |
|  | GSVIVT00034043001 | Nuclear pore protein 84/107 containing protein | Unknown | |
|  | GSVIVT00010406001 | Nuclear pore protein 84/107 containing protein | Unknown | |
| miR066 | GSVIVT00033825001 | NBS-LRR type disease resistance protein | SR (disease resistence) | |
|  | GSVIVT00033831001 | NBS-LRR type disease resistance protein | SR (disease resistence) | |
| miR067 | no |  |  | |
| miR068 ^a^ | no |  |  | |
| miR069 | no |  |  | |
| miR070 | GSVIVT00034084001 | Conserved gene of unknown function | Unknown | |
|  | GSVIVT00034082001 | Conserved gene of unknown function | Unknown | |
|  | GSVIVT00034839001 | Pectin methylesterase 2 | GD | |
| miR071 | GSVIVT00014155001 | Pentatricopeptide repeat-containing protein, mitochondrial | SR( disease resistence) | |
|  | GSVIVT00030708001 | ABRH23 stress response | SR (ABA-regulated early genes) | |
|  | GSVIVT00022486001 | Phytochrome A | GD | |
| miR072 | no |  |  | |
| miR073 | no |  |  | |
| miR074 | GSVIVT00028533001 | Fragment | Unknown | |
| mir075 | no |  |  | |
| miR076 | no |  |  | |
| miR077 | no |  |  | |
| miR078 | GSVIVT00028940001 | ATP binding / protein binding / transmembrane receptor | ST | |
| miR079 | GSVIVT00029886001 | Pectin methlyesterase inhibitor protein 1 growth development | GD | |
|  | GSVIVT00020113001 | Conserved gene of unknown function | Unknown | |
|  | GSVIVT00015695001 | Radical-induced cell death 1-1 | ST (cell death sigal) | |
| miR080 | no |  |  | |
| miR081 | GSVIVT00011463001 | Conserved gene of unknown function | Unknown | |
|  | GSVIVT00011463001 | Conserved gene of unknown function | Unknown | |
|  | GSVIVT00016222001 | VERNALIZATION INDEPENDENCE 4 | Regulates flowering-time genes | |
|  | GSVIVT00015006001 | Conserved gene of unknown function | Unknown | |
|  | GSVIVT00011440001 | Conserved gene of unknown function | Unknown | |
|  | GSVIVT00017667001 | Conserved gene of unknown function | Unknown | |
|  | GSVIVT00021640001 | Conserved gene of unknown function | Unknown | |
|  | GSVIVT00002348001 | Conserved gene of unknown function | Unknown | |
|  | GSVIVT00038353001 | Conserved gene of unknown function | Unknown | |
|  | GSVIVT00002138001 | Conserved gene of unknown function | Unknown | |
|  | GSVIVT00009749001 | Conserved gene of unknown function | Unknown | |
|  | GSVIVT00026476001 | UDP-glucose:glycoprotein glucosyltransferase | M | |
|  | GSVIVT00018097001 | Conserved gene of unknown function | Unknown | |
|  | GSVIVT00015525001 | U11/U12 small nuclear ribonucleoprotein 25 kDa protein | Unknown | |
|  | GSVIVT00018255001 | LOB domain protein 1 | GD | |
|  | GSVIVT00003241001 | Conserved gene of unknown function | Unknown | |
|  | GSVIVT00034933001 | Conserved gene of unknown function | Unknown | |
|  | GSVIVT00005401001 | DUF1475 domain containing protein | Unknown | |
|  | GSVIVT00027137001 | Conserved gene of unknown function | Unknown | |
|  | GSVIVT00008674001 | Conserved gene of unknown function | Unknown | |
|  | GSVIVT00034039001 | Glycolate oxidase | M | |
|  | GSVIVT00032754001 | Beta transducin | Unknown | |
|  | GSVIVT00027956001 | Conserved gene of unknown function | Unknown | |
|  | GSVIVT00032799001 | Conserved gene of unknown function | Unknown | |
|  | GSVIVT00011613001 | Conserved gene of unknown function | Unknown | |
|  | GSVIVT00009314001 | Conserved gene of unknown function | Unknown | |
|  | GSVIVT00023881001 | Conserved gene of unknown function | Unknown | |
|  | GSVIVT00012568001 | ORF58d | Unknown | |
|  | GSVIVT00010544001 | Conserved gene of unknown function | Unknown | |
|  | GSVIVT00029918001 | Conserved gene of unknown function | Unknown | |
|  | GSVIVT00014398001 | Conserved gene of unknown function | Unknown | |
|  | GSVIVT00001730001 | Conserved gene of unknown function | Unknown | |
|  | GSVIVT00002026001 | gene of unknown function | Unknown | |
|  | GSVIVT00021446001 | Conserved gene of unknown function | Unknown | |
|  | GSVIVT00018644001 | Conserved gene of unknown function | Unknown | |
|  | GSVIVT00038342001 | Conserved gene of unknown function | Unknown | |
|  | GSVIVT00030617001 | Conserved gene of unknown function | Unknown | |
|  | GSVIVT00037943001 | Conserved gene of unknown function | Unknown | |
|  | GSVIVT00038027001 | Conserved gene of unknown function | Unknown | |
|  | GSVIVT00024296001 | scarecrow gene regulator | GD | |
|  | GSVIVT00021741001 | Conserved gene of unknown function | Unknown | |
|  | GSVIVT00032512001 | Conserved gene of unknown function | Unknown | |
|  | GSVIVT00036766001 | Conserved gene of unknown function | Unknown | |
|  | GSVIVT00023917001 | KH domain-containing protein | ST | |
|  | GSVIVT00017285001 | Conserved gene of unknown function | Unknown | |
|  | GSVIVT00015985001 | Conserved gene of unknown function | Unknown | |
|  | GSVIVT00010334001 | Conserved gene of unknown function | Unknown | |
|  | GSVIVT00004888001 | Conserved gene of unknown function | Unknown | |
|  | GSVIVT00017443001 | Zinc knuckle family protein | GD | |
|  | GSVIVT00019315001 | Cell division cycle protein 23 homolog | GD | |
|  | GSVIVT00006412001 | Translocase of chloroplast 90, chloroplastic | M | |
|  | GSVIVT00037676001 | Conserved gene of unknown function | Unknown | |
|  | GSVIVT00018941001 | cinnamoyl-CoA reductase | M | |
|  | GSVIVT00037542001 | Conserved gene of unknown function | Unknown | |
|  | GSVIVT00005036001 | Conserved gene of unknown function | Unknown | |
|  | GSVIVT00028270001 | Conserved gene of unknown function | Unknown | |
|  | GSVIVT00027304001 | lactoylglutathione lyase | SR (salt stress response) | |
|  | GSVIVT00010250001 | Conserved gene of unknown function | Unknown | |
|  | GSVIVT00004579001 | Conserved gene of unknown function | Unknown | |
|  | GSVIVT00036742001 | Conserved gene of unknown function | Unknown | |
| miR082 ^b^ | GSVIVT00038807001 | LRRNT_2 domain containing protein | SR | |
|  | GSVIVT00038792001 | Conserved gene of unknown function | Unknown | |
|  | GSVIVT00028247001* | RADIALIS | Unknown | |
| miR083 | no |  |  | |
| miR084 | GSVIVT00030247001* | Carboxylesterase | M | |
|  | GSVIVT00025764001* | binding / protein binding | Unknown | |
|  | GSVIVT00037675001* | U-box domain-containing protein 32 | M | |
| miR085-1,-2 | GSVIVT00016287001 | IBS1 (IMPAIRED IN BABA-INDUCED STERILITY 1) | SR (salt stress) | |
| miR086 | GSVIVT00026212001 | similar to CG7927 CG7927-PA | Unknown | |
| miR087 ^b^ -1,-2,-3 | GSVIVT00035427001* | Ubiquitin-like domain-containing CTD phosphatase | M | |
|  | GSVIVT00021349001* | WD-repeat membrane protein | ST | |
|  | GSVIVT00008983001* | Conserved gene of unknown function | Unknown | |
|  | GSVIVT00009606001* | VAMP-associated protein | Unknown | |
| miR088 ^a^ | GSVIVT00025360001* | Squamosa promoter binding protein-homologue 5 | TF | |
|  | GSVIVT00036375001* | 3-methyladenine DNA glycosylase | M | |
|  | GSVIVT00014029001* | Cellulose synthase D4 | GD | |
|  | GSVIVT00009036001 | Conserved gene of unknown function | Unknown | |
|  | GSVIVT00023643001 | oxidoreductase | M | |
| mir089 | no |  |  | |
| mir090 | GSVIVT00025184001 | Protein phosphatase 2C | M | |
|  | GSVIVT00029539001 | DUF674 domain containing protein | Unknown | |
|  | GSVIVT00021568001 | TTG1 | M (anthocyanins biosynthesis) | |
|  | GSVIVT00033126001 | BZIP transcription factor | TF (Disease resistence) | |
|  | GSVIVT00017904001 | Conserved gene of unknown function | Unknown | |
|  | GSVIVT00034256001 | Light repressible receptor protein kinase | SR | |
| miR091 | no |  |  | |
| miR092 | GSVIVT00003201001 | TIR-NBS-LRR type disease resistance protein disease stress | SR (disease resistence) | |
| miR093 ^b^ | no |  |  | |
| miR094 ^b^ | GSVIVT00033535001 | gene of unknown function | Unknown | |
| miR095 | no |  |  | |
| miR096 | GSVIVT00018149001 | Vacuolar protein sorting-associated protein 33 Unknown  homolog | | |
|  | GSVIVT00027930001 | Ubiquitin carboxyl-terminal hydrolase | | M |
|  | GSVIVT00030327001 | F24J5.19 protein (Peptide transporter like) | | Unknown |
| miR097 | GSVIVT00034031001 | Conserved gene of unknown function | | Unknown |
|  | GSVIVT00016764001 | Pattern formation protein EMB30 | | GD |
| miR098 | no |  | |  |
| miR099-1,-2 | GSVIVT00032536001 | Syntaxin-32 | | Unknown |
| miR100 | GSVIVT00030480001 | At1g70950 protein | | Unknown |
|  | GSVIVT00034043001 | Nuclear pore protein 84/107 containing protein | | Unknown |
|  | GSVIVT00002764001 | Conserved gene of unknown function | | Unknown |
| miR101 | no |  | |  |
| miR102 ^b^ | GSVIVT00030051001* | 14-3-3 GF14 iota | | Unknown |
|  | GSVIVT00030372001* | E3 ubiquitin ligase | | M |
|  | GSVIVT00036625001* | myosin | | Unknown |
|  | GSVIVT00005740001* | DNA polymerase V | | Unknown |
|  | GSVIVT00035427001 | Ubiquitin-like domain-containing CTD phosphatase | | M |
| miR103 | GSVIVT00033443001 | transporter | | Transport function |
|  | GSVIVT00031829001 | CG6511-PA | | Unknown |
|  | GSVIVT00028701001 | T12J13.2 protein (F20H23.27 protein) | | Unknown |
|  | GSVIVT00003508001 | EFR (EF-TU RECEPTOR) | | SR (disease resistence) |
|  | GSVIVT00005322001 | Aluminum-activated malate transporter | | SR |
|  | GSVIVT00014216001 | Conserved gene of unknown function | | Unknown |
|  | GSVIVT00001947001 | Peptidase_C12 domain containing protein | | Unknown |
|  | GSVIVT00020456001 | Receptor kinase 2 | | M |
|  | GSVIVT00016011001 | alliin lyase | | Defense response |
| miR104 ^b^ | GSVIVT00033078001 | Bet_v_I domain containing protein | | SR (disease resistence) |
|  | GSVIVT00027051001 | tetratricopeptide repeat (TPR)-containing protein stress response | | Putative TAP (ABA,salt,drought stress response) |
|  | GSVIVT00020458001 | Conserved gene of unknown function | | Unknown |
|  | GSVIVT00037989001 | Disease resistance gene disease stress | | SR (disease resistence) |
|  | GSVIVT00011364001 | binding | | Unknown |
|  | GSVIVT00034992001 | similar to CG2614-PA, partial | | Unknown |
| miR105 | no |  | |  |
| miR106 | GSVIVT00014595001 | Conserved gene of unknown function | | Unknown |

Notes: miRNAs with ^a^ and ^b^ represent non-conserved miRNAs, of which ones with ^a^ were a few plants specific miRNAs, while ones with ^b^ were vitis plants specific miRNAs; the target genes with * were predicted by wang et al..
