# Supplementary material for: Identification of microRNAs from Amur grape (vitis amurensis Rupr.) by deep sequencing and analysis of microRNA variations with bioinformatics
Source: BMC Genomics. 2012 Mar 29;13:122. doi: 10.1186/1471-2164-13-122 (PMC3353164; doi:10.1186/1471-2164-13-122)
Supplement: Additional file 8 — List of primers of miRNAs used for miR-5'RACE and miR-3'RACE. [file 1471-2164-13-122-S8.DOC]

| Table S5 | | | | |
| --- | --- | --- | --- | --- |
| Abundance | Conservation | miRNA ID | 5' -primer (5’→3’) | 3'-primer (5’→3’) |
| Low abundance | Conserved | va-miR398b | TTTTTTTTTTTTCTCAGGTCGCCCCTG | GGAGTAGAAATGTGTTCTCAGGTCGCC |
|  |  | va-miR399c | TTTTTTTTTTAAAGGAGAGTTGCCCTG | GGAGTAGAAATGCCAAAGGAGAGTTGC |
|  |  | va-miR828a | TTTTTTTTTTCTCAAATGAGTATTCCA | GGAGTAGAAATCTTGCTCAAATGAGTA |
|  | Non-conserved | va-miR005 | TTTTTTTTTTTGAGGGGGGATTGTCAT | GGAGTAGAAATTTGCATGAGGGGGGAT |
|  |  | va-miR011 | TTTTTTTTTTGCAGGAGAGACGGCACT | GGAGTAGAAATTGTCGCAGGAGAGACG |
|  |  | va-miR020 | TTTTTTTTTTTTAACCATTTAAATTTA | GGAGTAGAAATTATTAACCATTTAAAT |
|  |  | va-miR028 | TTTTTTTTTTTACACTTTTTATTTTTT | GGAGTAGAAATGGATACACTTTTTATT |
|  |  | va-miR029 | TTTTTTTTTTGAAGGGGAGCACGTGCA | GGAGTAGAAATGGAGAAGGGGAGCACG |
|  |  | va-miR032 | TTTTTTTTTTTAGAAAATGTTTTTAAC | GGAGTAGAAATGATTGTAGAAAATGTT |
|  |  | va-miR037 | TTTTTTTTTTCACTATATAAATATGAA | GGAGTAGAAATGACACTATATAAATAT |
|  |  | va-miR045 | TTTTTTTTTTGTGGGAACGCATTGAGC | GGAGTAGAAATCGAGTGGGAACGCATT |
|  |  | va-miR048 | TTTTTTTTTTTTAGTAGCTTAACATGG | GGAGTAGAAATCAATTAGTAGCTTAAC |
|  |  | va-miR052 | TTTTTTTTTTAAGTTCAAGTGAAAATT | GGAGTAGAAATATTAAGTTCAAGTGAA |
|  |  | va-miR063 | TTTTTTTTTTGAAGCGTTTTTAATATT | GGAGTAGAAACTAGGAAGCGTTTTTAA |
|  |  | va-miR066 | TTTTTTTTTTTTTTAAATTTTTTTATC | GGAGTAGAAACGTCTTTTAAATTTTTT |
|  |  | va-miR074 | TTTTTTTTTTTTTATTGTTTTTTAATT | GGAGTAGAAACAGGTTTATTGTTTTTT |
|  |  | va-miR077 | TTTTTTTTTTCATCATTATATAAAACG | GGAGTAGAAAATTTCATCATTATATAA |
|  |  | va-miR082 | TTTTTTTTTTTTATTAGGGAATTAAGT | GGAGTAGAAAATATTATTAGGGAATTA |
|  |  | va-miR095 | TTTTTTTTTTAAATGCTTGATTAAATG | GGAGTAGAAAAACAAATGCTTGATTAA |
|  |  | va-miR098 | TTTTTTTTTTTGACCTATTTAATAACT | GGAGTAGAAAAAATTGACCTATTTAAT |
| High abundance | Conserved | va-miR156e | TTTTTTTTTTCAGAGGAGAGTGAGCAC | GGAGTAGAAATGACAGAGGAGAGTGAG |
|  |  | va-miR160c | TTTTTTTTTTTGGCTCCCTGTATGCCA | GGAGTAGAAATGCCTGGCTCCCTGTAT |
|  |  | va-miR162 | TTTTTTTTTTTCGATAAACCTCTGCAT | GGAGTAGAAATCGATAAACCTCTGCAT |
|  |  |  |  |  |
|  |  | va-miR164c | TTTTTTTTTTGAAGCAGGGCACGTGCA | GGAGTAGAAATGGAGAAGCAGGGCACG |
|  |  | va-miR166c | TTTTTTTTTTACCAGGCTTCATTCCTC | GGAGTAGAAATCGGACCAGGCTTCATT |
|  |  | va-miR169m | TTTTTTTTTTCCAAGGATGACTTGCCG | GGAGTAGAAATGAGCCAAGGATGACTT |
|  |  | va-miR171c | TTTTTTTTTTTGAGCCGTGCCAATATC | GGAGTAGAAATGATTGAGCCGTGCCAA |
|  |  | va-miR172c | TTTTTTTTTTTCTTGATGATGCTGCAG | GGAGTAGAAAGGAATCTTGATGATGCT |
|  |  | va-miR408 | TTTTTTTTTTACTGCCTCTTCCCTGGC | GGAGTAGAAAATGCACTGCCTCTTCCC |
|  |  | va-miR535a | TTTTTTTTTTAACGAGAGAGAGCACGC | GGAGTAGAAATGACAACGAGAGAGAGC |
|  | Non-conserved | va-miR001 | TTTTTTTTTTTATTGGATCCGTCGGGA | GGAGTAGAAATTTTTTTATTGGATCCG |
|  |  | va-miR007 | TTTTTTTTTTACTCGCACTCATGCCGT | GGAGTAGAAATTTCCGACTCGCACTCA |
|  |  | va-miR016 | TTTTTTTTTTTGTGATCTTGTTGTTTC | GGAGTAGAAATTCTTGTGATCTTGTTG |
|  |  | va-miR018 | TTTTTTTTTTCGGACCAGGCTTCATTC | GGAGTAGAAATTCTCGGACCAGGCTTC |
|  |  | va-miR023 | TTTTTTTTTTATGATCATCAACAAACA | GGAGTAGAAATTAGATGATCATCAACA |
|  |  | va-miR046 | TTTTTTTTTTGGAGAGATGGCACCTGC | GGAGTAGAAATCCCAGGAGAGATGGCA |
|  |  | va-miR047 | TTTTTTTTTTAGTTCATCCAAGCACCA | GGAGTAGAAATCACAAGTTCATCCAAG |
|  |  | va-miR049 | TTTTTTTTTTATAAGGTACTTTTAGCT | GGAGTAGAAATCAATAAGGTACTTTTA |
|  |  | va-miR057 | TTTTTTTTTTGAAGCCGGTGGGGGACC | GGAGTAGAAAGTTGGAAGCCGGTGGGG |
|  |  | va-miR062 | TTTTTTTTTTGTTATAGGATCTTGGAT | GGAGTAGAAACTATGTTATAGGATCTT |
