# Supplementary material for: Identification of microRNAs from Amur grape (vitis amurensis Rupr.) by deep sequencing and analysis of microRNA variations with bioinformatics
Source: BMC Genomics. 2012 Mar 29;13:122. doi: 10.1186/1471-2164-13-122 (PMC3353164; doi:10.1186/1471-2164-13-122)
Supplement: Additional file 9 — List of primers of va-miR-LDs used for miR-RACE. [file 1471-2164-13-122-S9.DOC]

| Table S6 | | |
| --- | --- | --- |
| miRNA ID | 5' -primer (5’→3’) | 3'-primer (5’→3’) |
| miR156a | TTTTTTTTTTCAGAAGAGAGGGAGCAC | GGAGTAGAAATGACAGAAGAGAGGGAG |
| miR166a | TTTTTTTTTTACCAGGCTTCATTCCTG | GGAGTAGAAATCGGACCAGGCTTCATT |
| miR166h | TTTTTTTTTTCCAGGCTTCATTCCCCC | GGAGTAGAAATCGGACCAGGCTTCATT |
| miR169b | TTTTTTTTTTCCAAGGATGGCTTGCCG | GGAGTAGAAATGAGCCAAGGATGGCTT |
| miR169l | TTTTTTTTTTCAAGGATGACTTGCCGT | GGAGTAGAAAGAGCCAAGGATGACTTG |
| miR169o | TTTTTTTTTTCAAGGATGACTTGCCGC | GGAGTAGAAAGAGCCAAGGATGACTTG |
| miR171c/d | TTTTTTTTTTGCCGTGCCAATATCACG | GGAGTAGAAATTGAGCCGTGCCAATAT |
