# Supplementary material for: Identification of microRNAs from Amur grape (vitis amurensis Rupr.) by deep sequencing and analysis of microRNA variations with bioinformatics
Source: BMC Genomics. 2012 Mar 29;13:122. doi: 10.1186/1471-2164-13-122 (PMC3353164; doi:10.1186/1471-2164-13-122)
Supplement: Supplementary file 9 [file 1471-2164-13-122-S1.DOC]

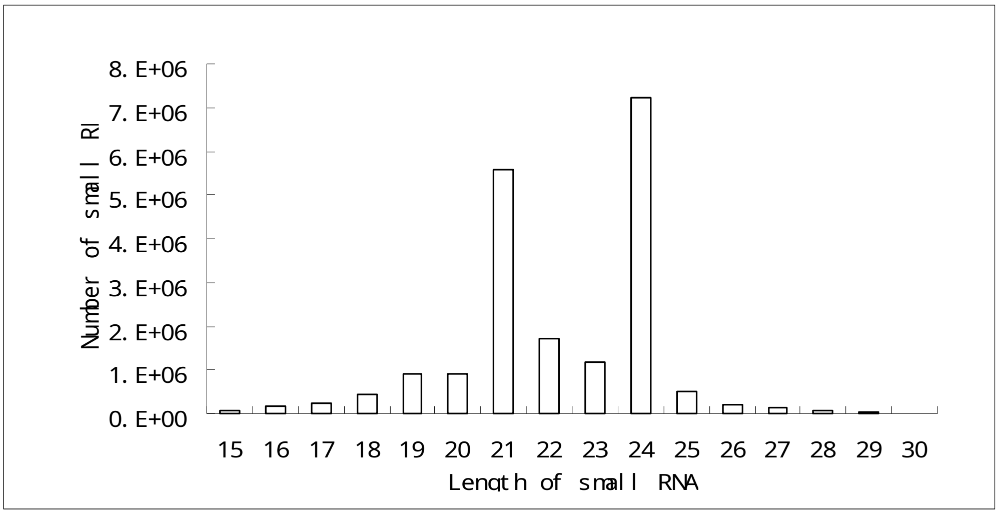
8. E+06
7. E+06
6. E+06
5. E+06
4. E+06
3. E+06
2. E+06
1. E+06
0. E+00
Number of small R
15
16
17
18
19
20
21
22
23
24
25
26
27
28
29
30
Length of small RNA
